# Supplementary material for: Analysing linear multivariate pattern transformations in neuroimaging data
Source: PLoS One. 2019 Oct 15;14(10):e0223660. doi: 10.1371/journal.pone.0223660 (PMC6793861; doi:10.1371/journal.pone.0223660)
Supplement: S1 Fig — (PDF) [file pone.0223660.s001.pdf]

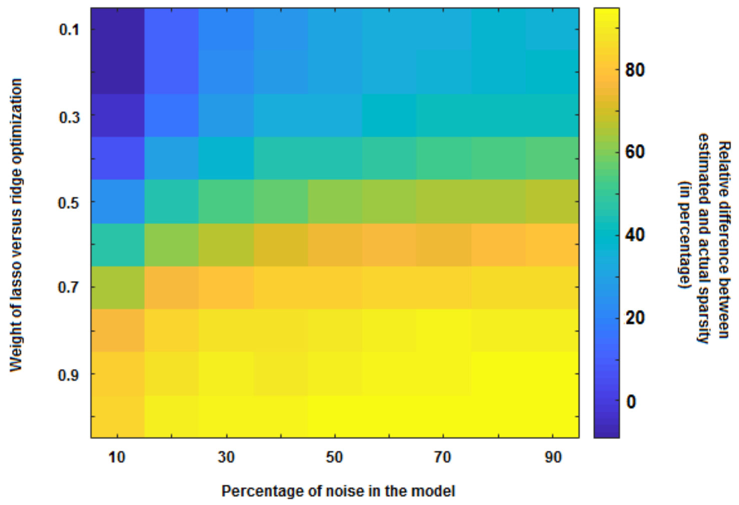

Fig S1. Percentage increment in estimating the simulated percentage of sparsity using different elastic net approaches. We simulated MV-interactions (with transformations between the two ROIs having a sparsity of 50%) and we estimated the mapping using different types of elastic net approaches, each of which consisted of a specific weighted combination of LASSO and ridge regression. The dimension of the simulated MV-pattern matrices was 224 (voxels) x 96 (stimuli) for the input ROI and 256 (voxels) x 96 (stimuli) for the output ROI, resembling the dimension of the actual early visual cortex (EVC) and fusiform face area (FFA) MV-pattern matrices. Regardless of the regularisation method, the estimated percentage of sparsity increases with increasing levels of noise. Furthermore, at low noise levels, the sparsity estimates improve slightly when the weight for ridge regression is higher than the weight for LASSO.
