# Supplementary material for: Analysing linear multivariate pattern transformations in neuroimaging data
Source: PLoS One. 2019 Oct 15;14(10):e0223660. doi: 10.1371/journal.pone.0223660 (PMC6793861; doi:10.1371/journal.pone.0223660)
Supplement: S2 Fig — (PDF) [file pone.0223660.s002.pdf]

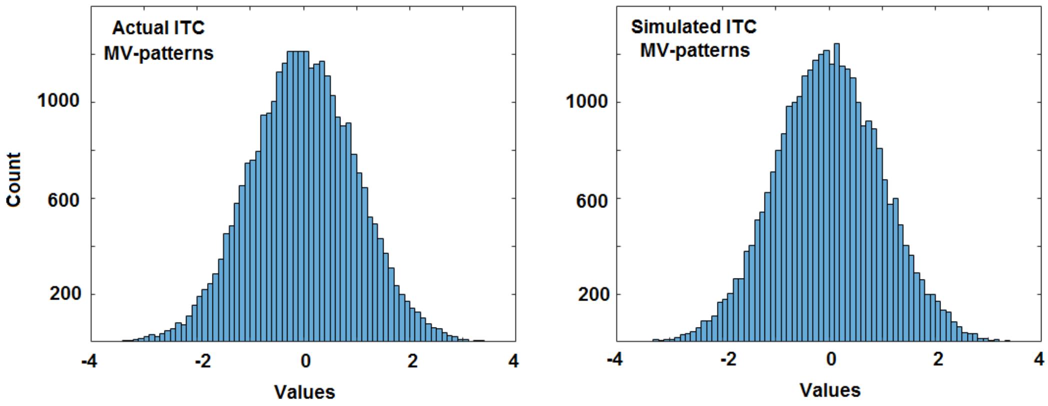

Fig S2. Histogram associated with the values of the actual (left panel) and simulated (right panel) MV-patterns of the inferior temporal cortex (ITC) for one subject. As for the Monte Carlo approach, the simulated ITC patterns have been generated as $Y = (1-\gamma)TX/\|TX\|_F + \gamma N/\|N\|_F$ where X denotes the EVC MV-pattern matrix for the subject, γ is a parameter representing the weight of the noise in the model (set as equal to 0.6 in this realisation), N is random noise and T is a simulated transformation (whose values follow a standard normal distribution).
