# Supplementary material for: Analysing linear multivariate pattern transformations in neuroimaging data
Source: PLoS One. 2019 Oct 15;14(10):e0223660. doi: 10.1371/journal.pone.0223660 (PMC6793861; doi:10.1371/journal.pone.0223660)
Supplement: S3 Fig — (PDF) [file pone.0223660.s003.pdf]

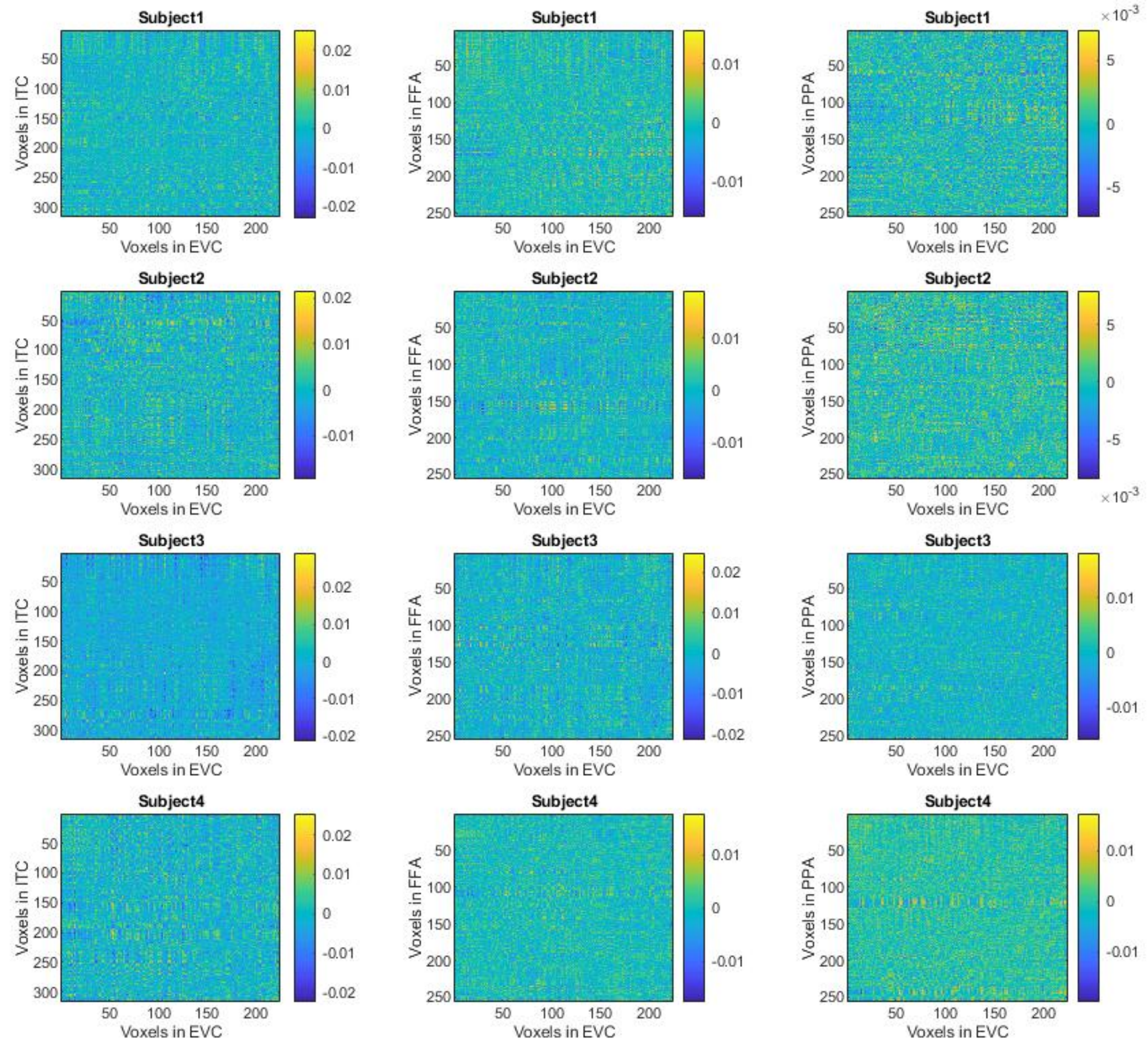

Fig S3. Estimated transformation for each subject and pair of ROIs. Each row shows the transformations between the MV-patterns of early visual cortex-EVC and the patterns of the other ROIs (inferior temporal cortex-ITC, fusiform face area-FFA and parahippocampal place area-PPA) for one subject. The columns refer to the specific pairs of ROIs (from left to right: EVC->ITC, EVC->FFA, EVC->PPA).
