# Supplementary material for: Analysing linear multivariate pattern transformations in neuroimaging data
Source: PLoS One. 2019 Oct 15;14(10):e0223660. doi: 10.1371/journal.pone.0223660 (PMC6793861; doi:10.1371/journal.pone.0223660)
Supplement: S4 Fig — (PDF) [file pone.0223660.s004.pdf]

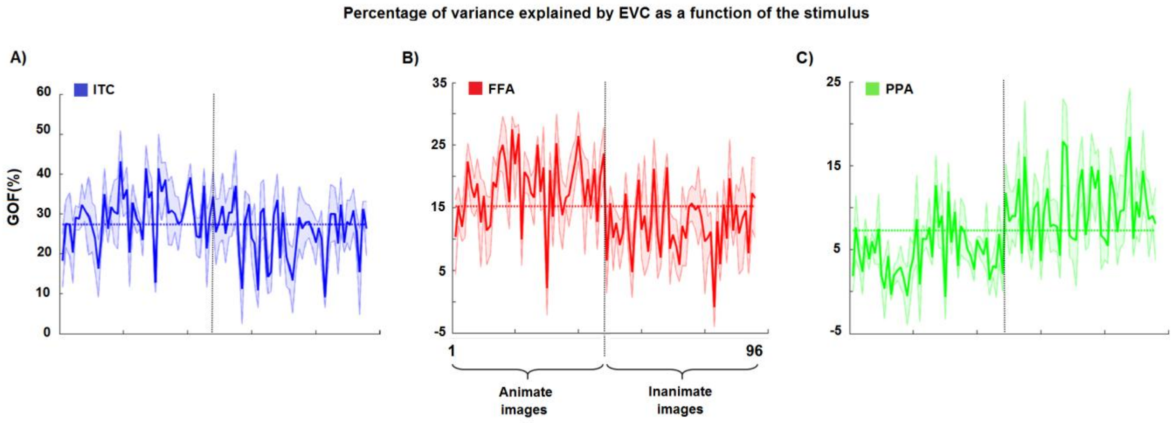

Fig S4. Goodness-of-fit (GOF) as a function of the stimuli. In order to compute the GOF, we here considered the squared Frobenius norm of the difference between the actual and estimated MV-pattern in the output ROI for each specific stimulus separately. **A)**, **B)** and **C)** The solid lines (and the shaded areas) denote the average percentages of GOF across the four subjects (and standard error), using the linear pattern transformations from EVC to the three output ROIs, as functions of the stimuli. The first 48 stimuli are animate images while the last 48 stimuli are inanimate images. A higher GOF (with respect to the mean) is evident for the animate stimuli for ITC and FFA, and for the inanimate stimuli for PPA.
