# Supplementary material for: Analysing linear multivariate pattern transformations in neuroimaging data
Source: PLoS One. 2019 Oct 15;14(10):e0223660. doi: 10.1371/journal.pone.0223660 (PMC6793861; doi:10.1371/journal.pone.0223660)
Supplement: S5 Fig — (PDF) [file pone.0223660.s005.pdf]

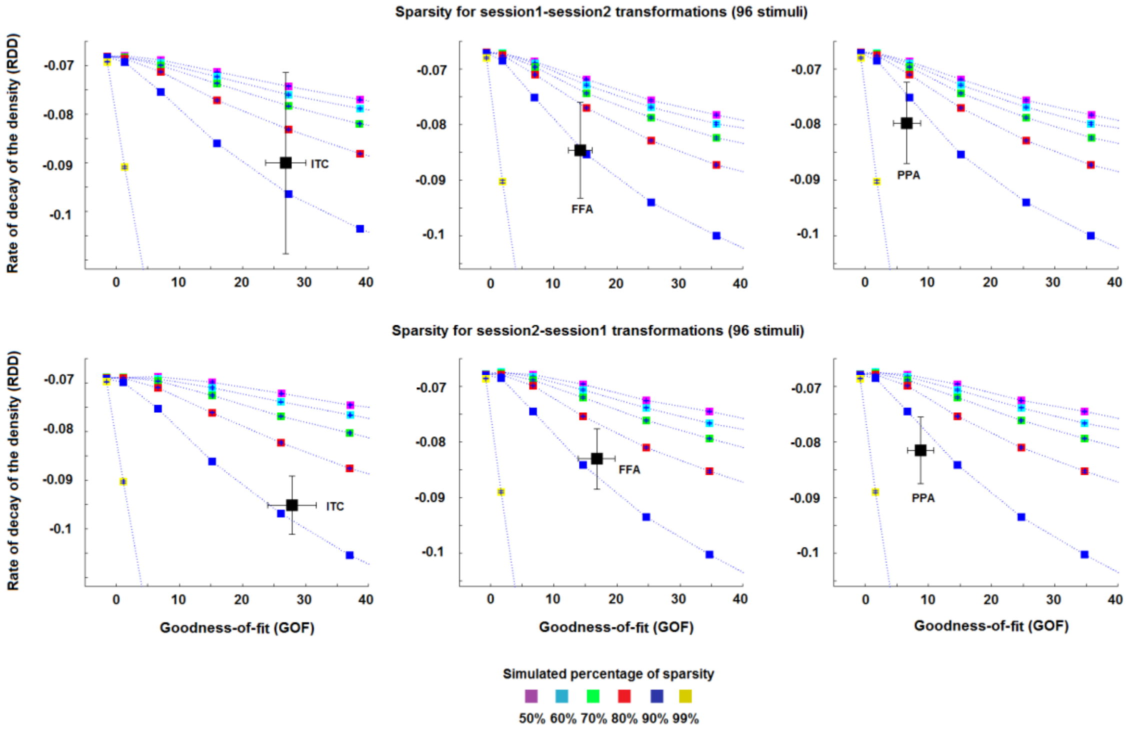

Fig S5. Estimated sparsity for the session1-session2 transformations and the session2-session1 transformations with 96 stimuli. The coloured squares (and their error bars) denote the mean (and the standard error of the mean) estimate of RDD and GOF across the 1000 simulation-realisations for each of the simulated percentages of sparsity, i.e. from 50% to 99%. The black squares (and their error bars) denote the mean (and the standard error of the mean) estimate of RDD and GOF across the four subjects. The results related to the session1-session2 transformations are in accordance with those obtained for the session2-session1 transformations.
