# Supplementary material for: Analysing linear multivariate pattern transformations in neuroimaging data
Source: PLoS One. 2019 Oct 15;14(10):e0223660. doi: 10.1371/journal.pone.0223660 (PMC6793861; doi:10.1371/journal.pone.0223660)
Supplement: S6 Fig — (PDF) [file pone.0223660.s006.pdf]

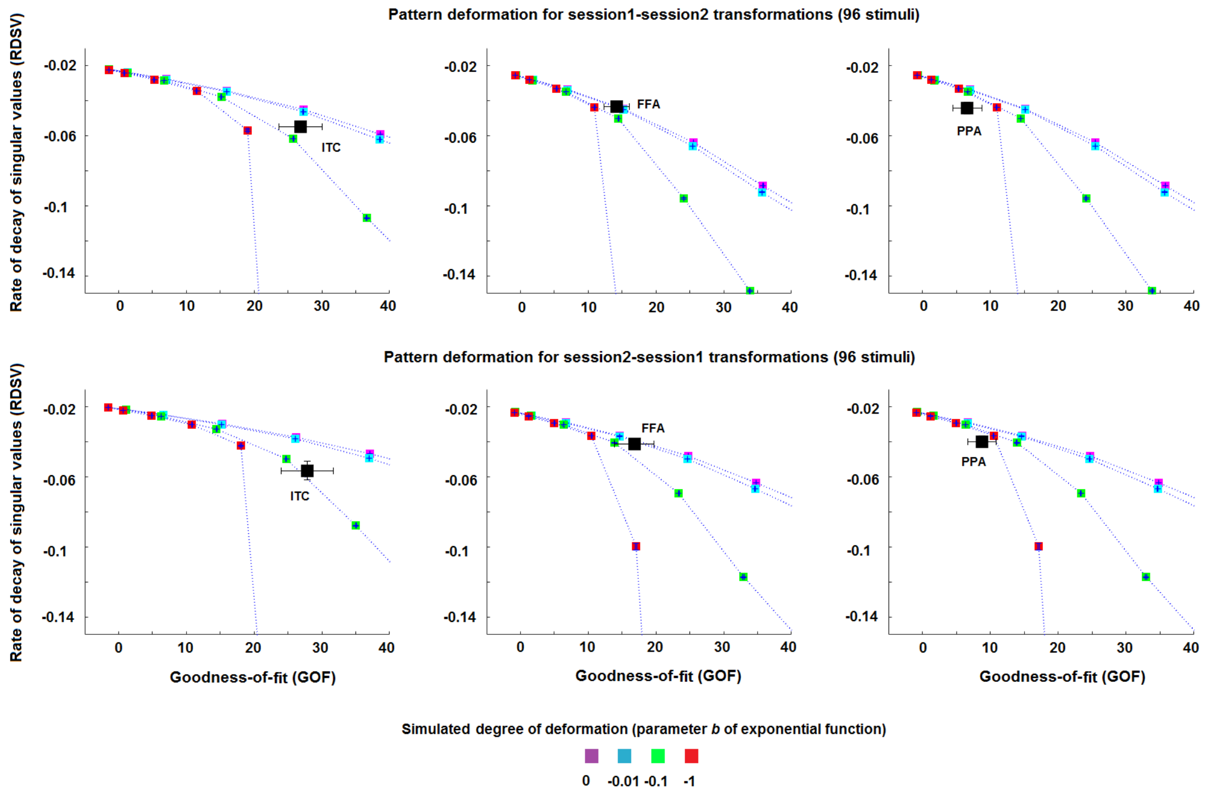

Fig S6. Estimated pattern deformation for the session1-session2 transformations and the session2-session1 transformations with 96 stimuli. The coloured squares (and their error bars) denote the mean (and the standard error of the mean) estimate of $RDSV$ and GOF across the 1000 simulation-realisations for each of the simulated degree of deformation (parameter b of an exponential function). The black squares (and their error bars) denote the mean (and the standard error of the mean) estimate of $RDSV$ and GOF across the four subjects. The results related to the session1-session2 transformations are in accordance with those obtained for the session2-session1 transformations.
