# Supplementary material for: Analysing linear multivariate pattern transformations in neuroimaging data
Source: PLoS One. 2019 Oct 15;14(10):e0223660. doi: 10.1371/journal.pone.0223660 (PMC6793861; doi:10.1371/journal.pone.0223660)
Supplement: S7 Fig — (PDF) [file pone.0223660.s007.pdf]

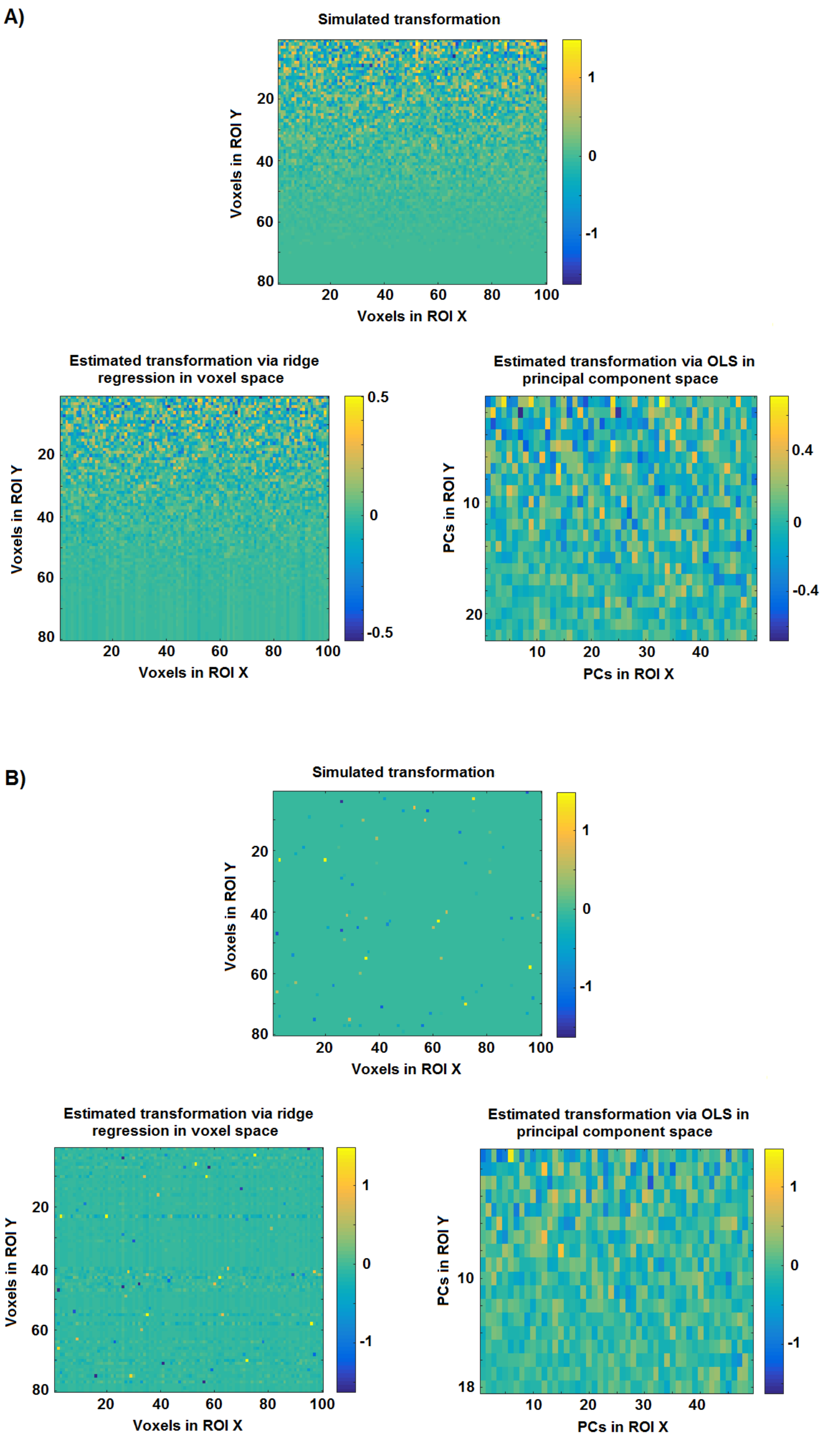

Fig S7. Simulated transformations between voxel spaces and between principal component spaces. The first row of panel A shows a simulated transformation with a specific spatial pattern in the voxel space. The second row of the same panel shows the voxel-by-voxel transformation estimated via ridge regression and the transformation obtained by applying an ordinary least square (OLS) approach on the principal components (PCs; here we selected the directions explaining 90% of the total variance for each ROI separately). Instead, the first row of panel B shows a simulated sparse matrix and the second row shows its estimate via ridge regression with the voxel spaces and OLS with the PC space. The simulated spatial pattern in panel A and the sparse matrix in panel B are only visible when using the original voxel spaces. This shows that changing the coordinate system (from voxel space to PC space, as well as to other coordinates) may result in loss of functionally relevant information, given that the reference to the voxel space is hidden by the change of coordinates.
