# Supplementary material for: Analysing linear multivariate pattern transformations in neuroimaging data
Source: PLoS One. 2019 Oct 15;14(10):e0223660. doi: 10.1371/journal.pone.0223660 (PMC6793861; doi:10.1371/journal.pone.0223660)

1. Masks used for region-of-interest definition

subject 1
session 1

manually defined cortex mask

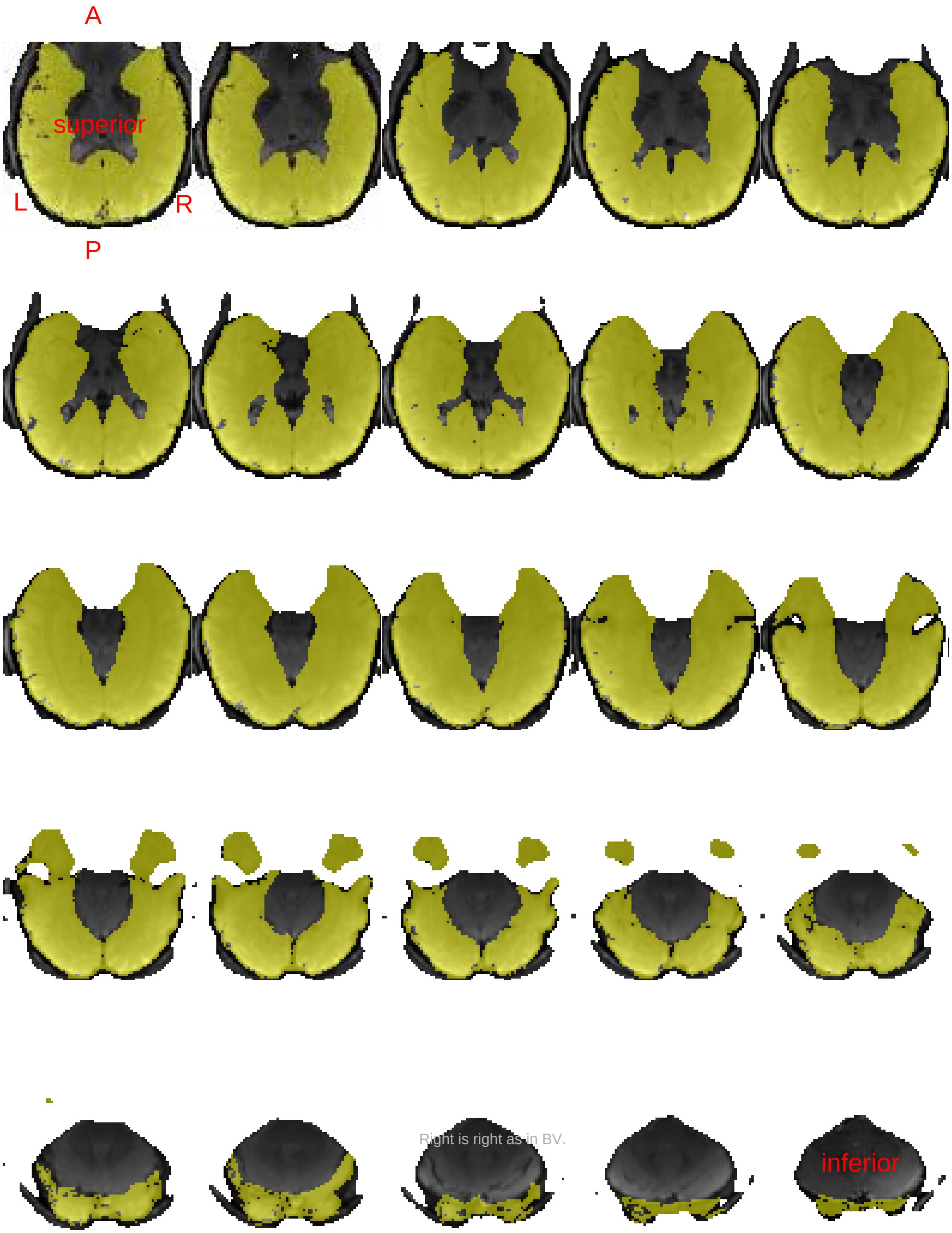

subject 1
session 1

global segmentation: aIT (red), pIT (green), EVA (blue), subcortical (white)

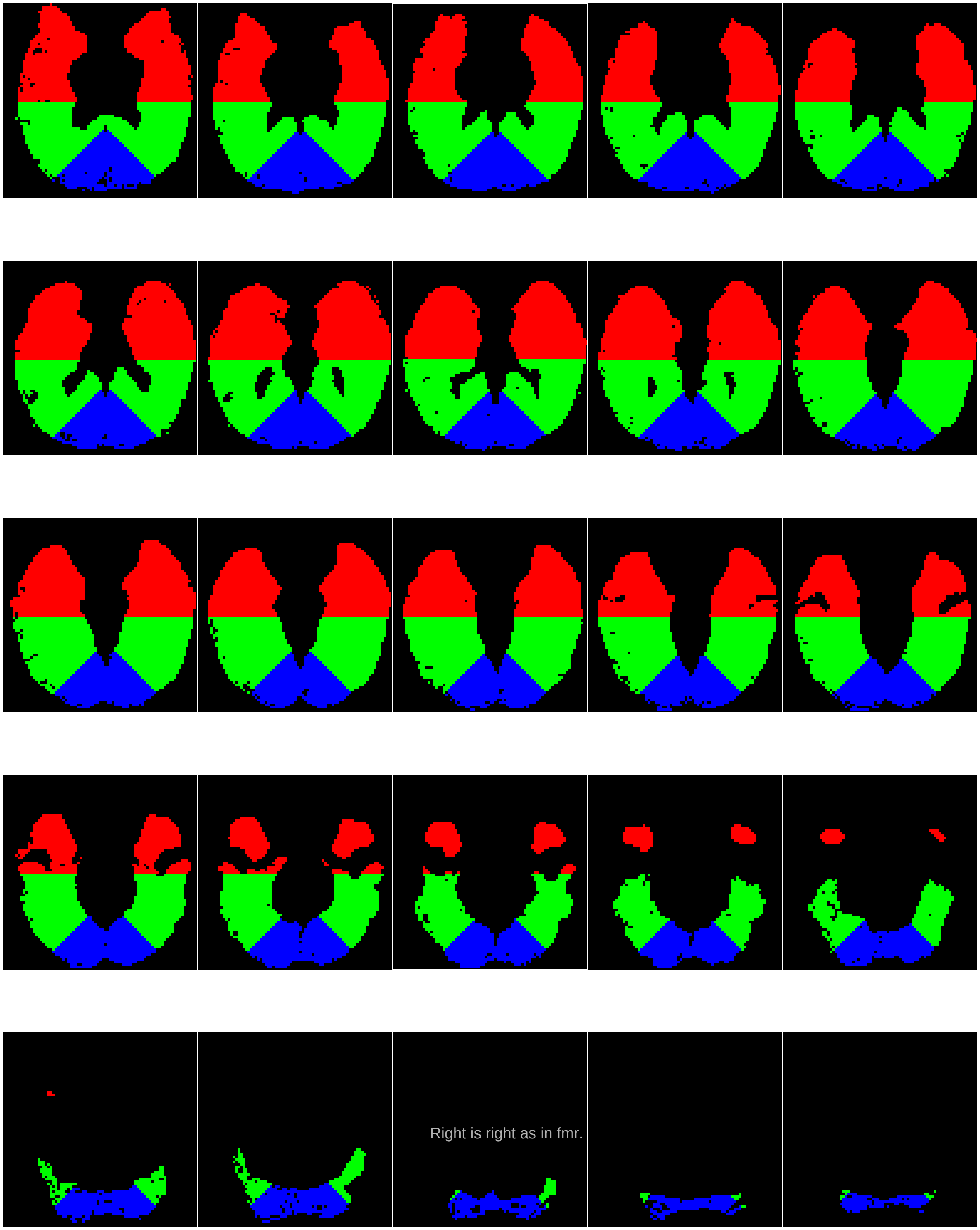

subject 1
session 2

manually defined cortex mask

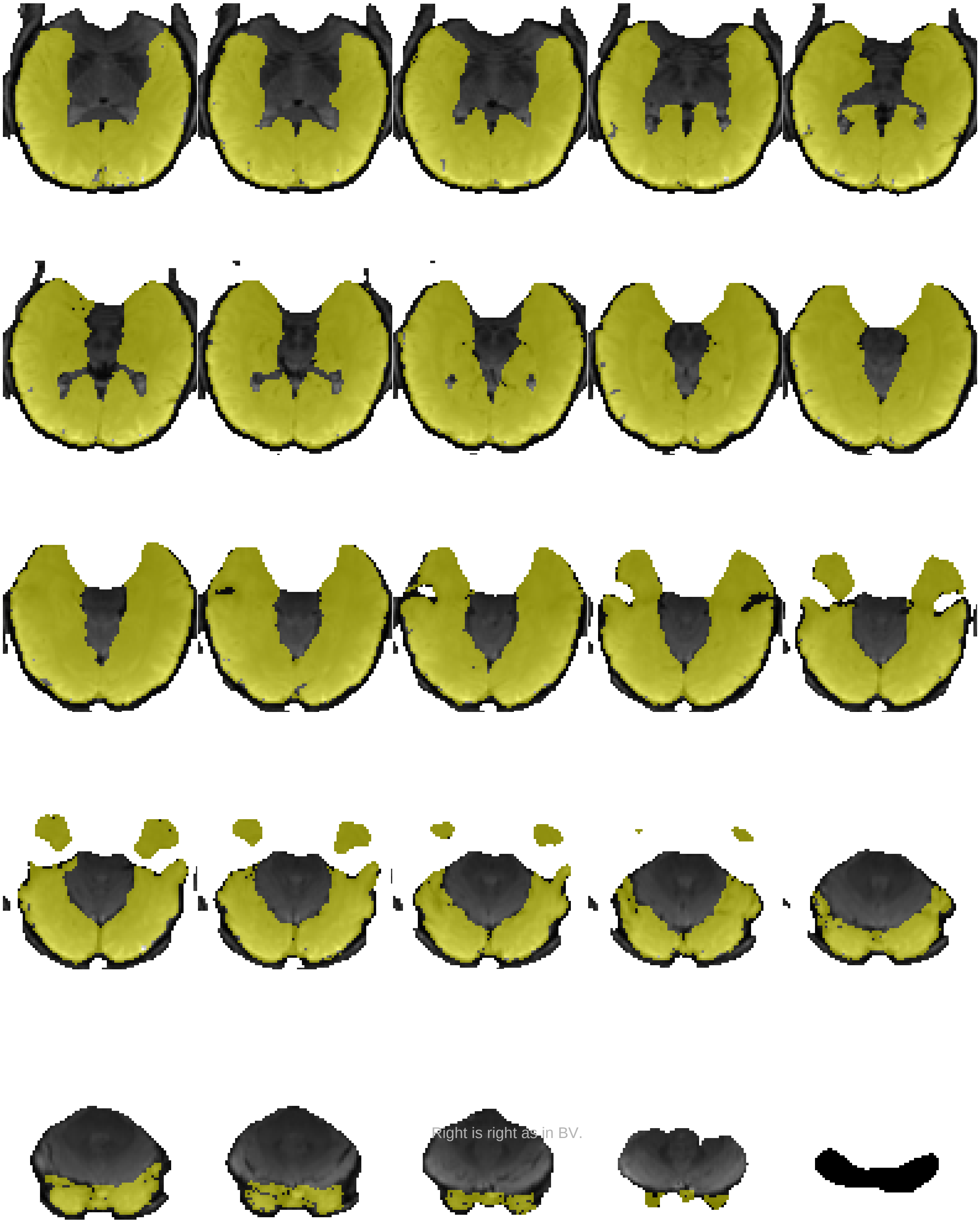

subject 1
session 2

global segmentation: aIT (red), pIT (green), EVA (blue), subcortical (white)

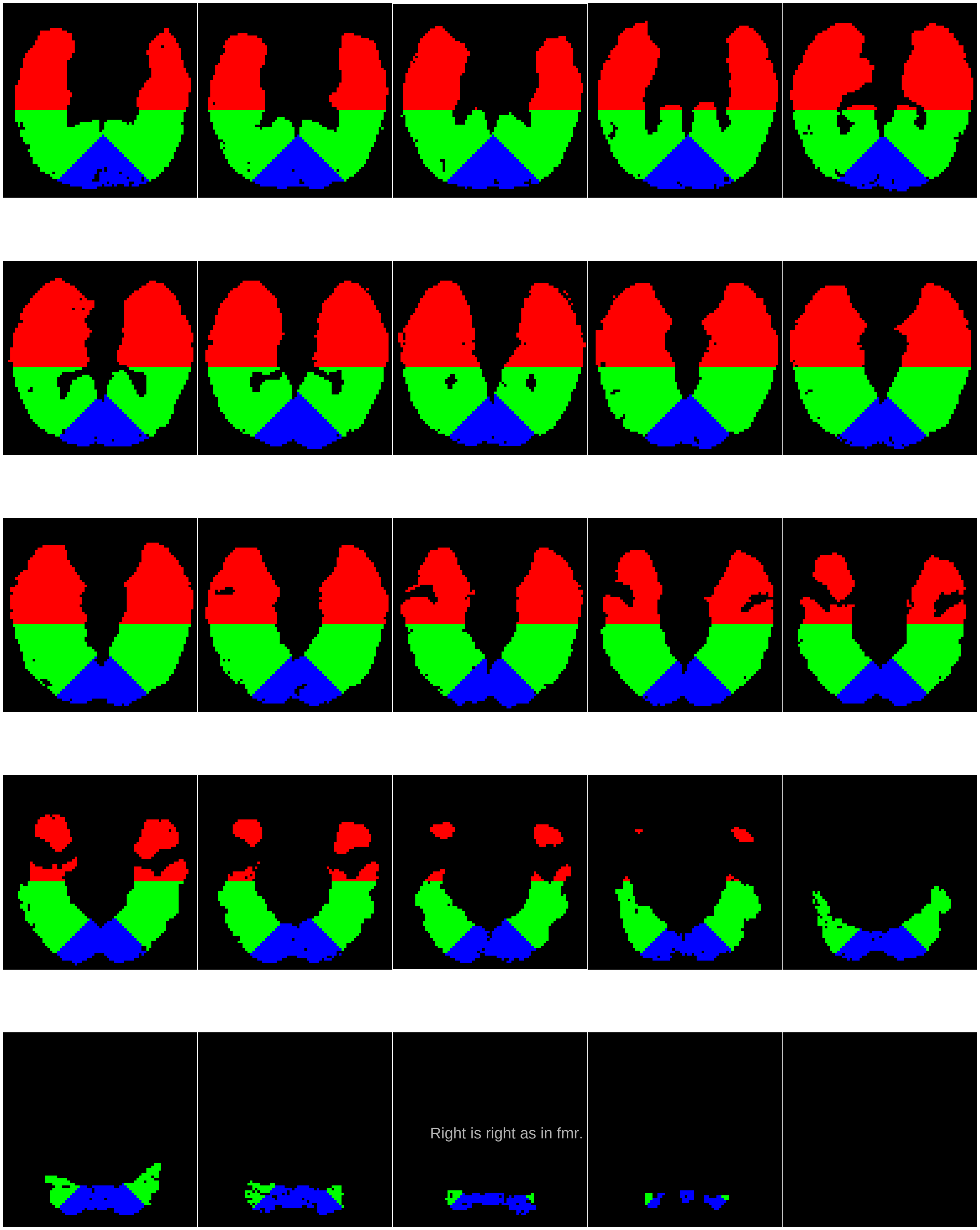

subject 2
session 1

manually defined cortex mask

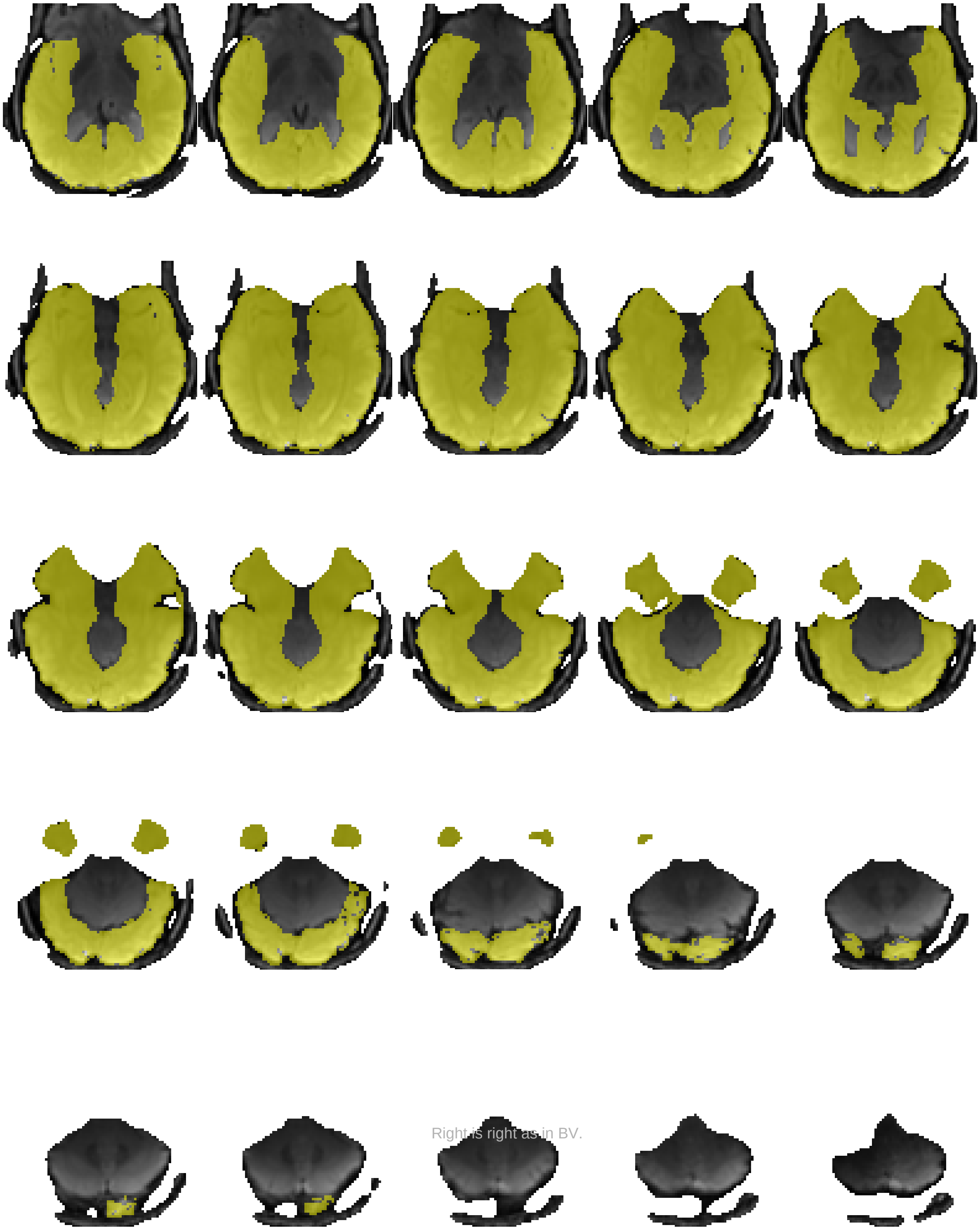

subject 2
session 1

global segmentation: aIT (red), pIT (green), EVA (blue), subcortical (white)

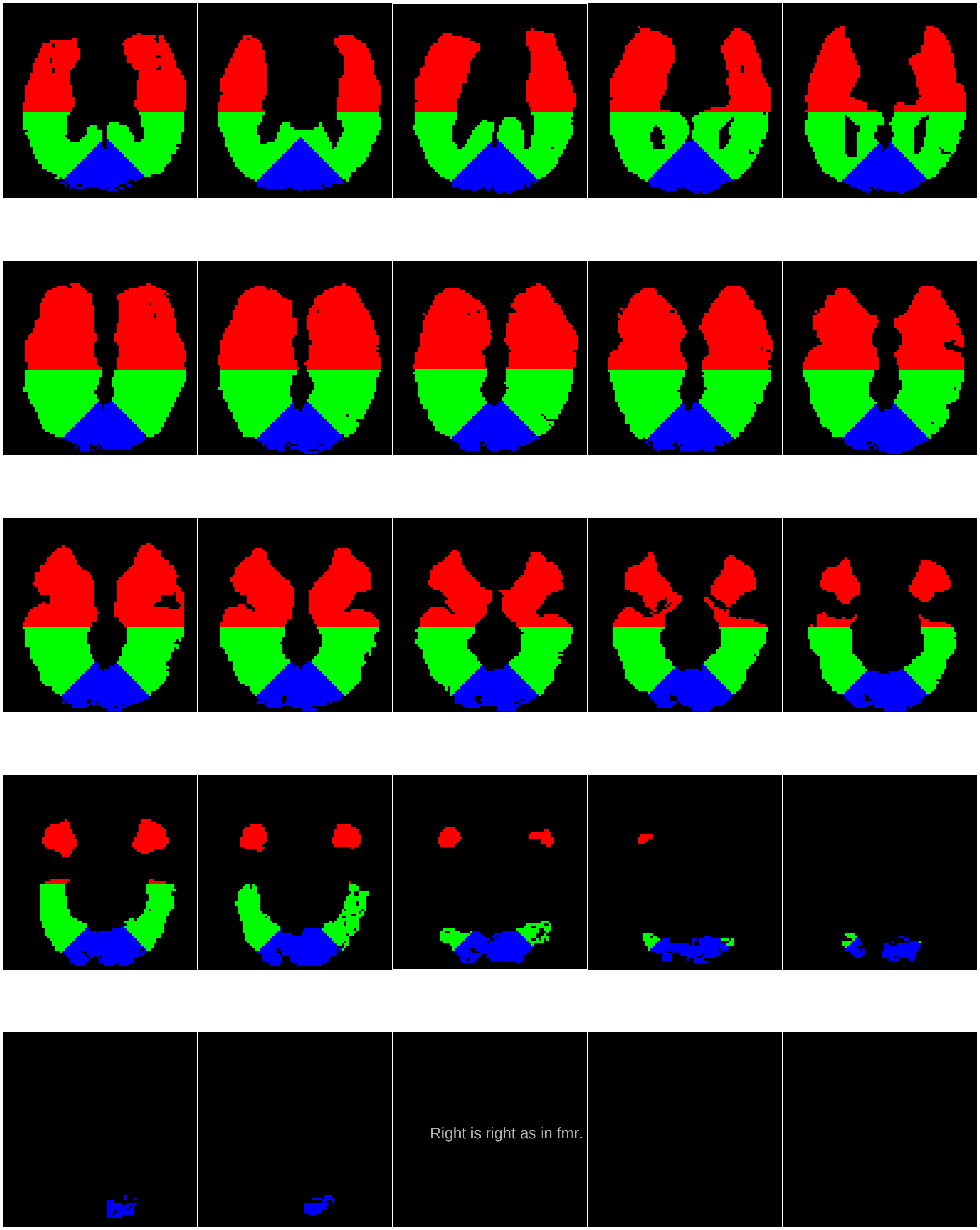

subject 2
session 3

manually defined cortex mask

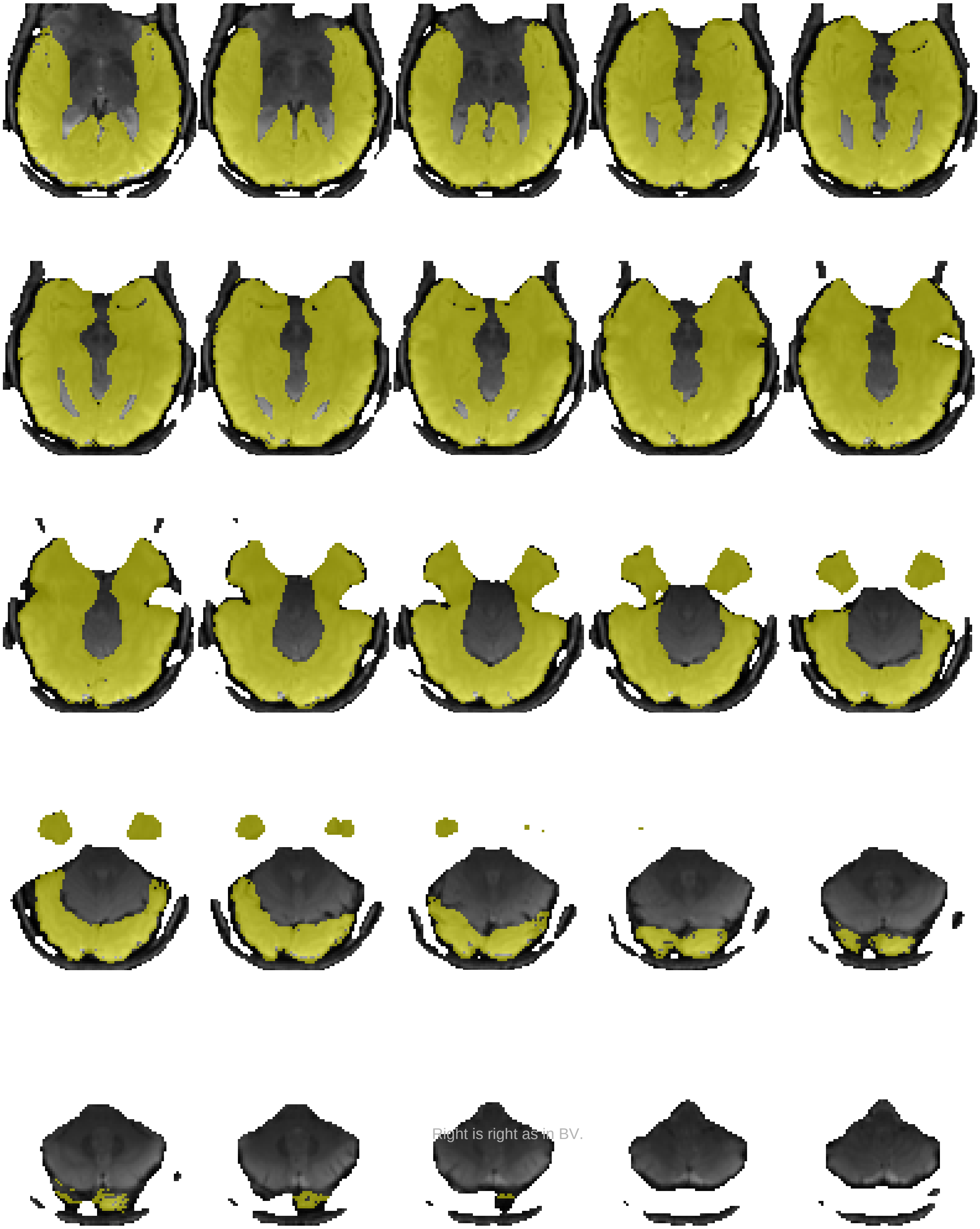

subject 2
session 3

global segmentation: aIT (red), pIT (green), EVA (blue), subcortical (white)

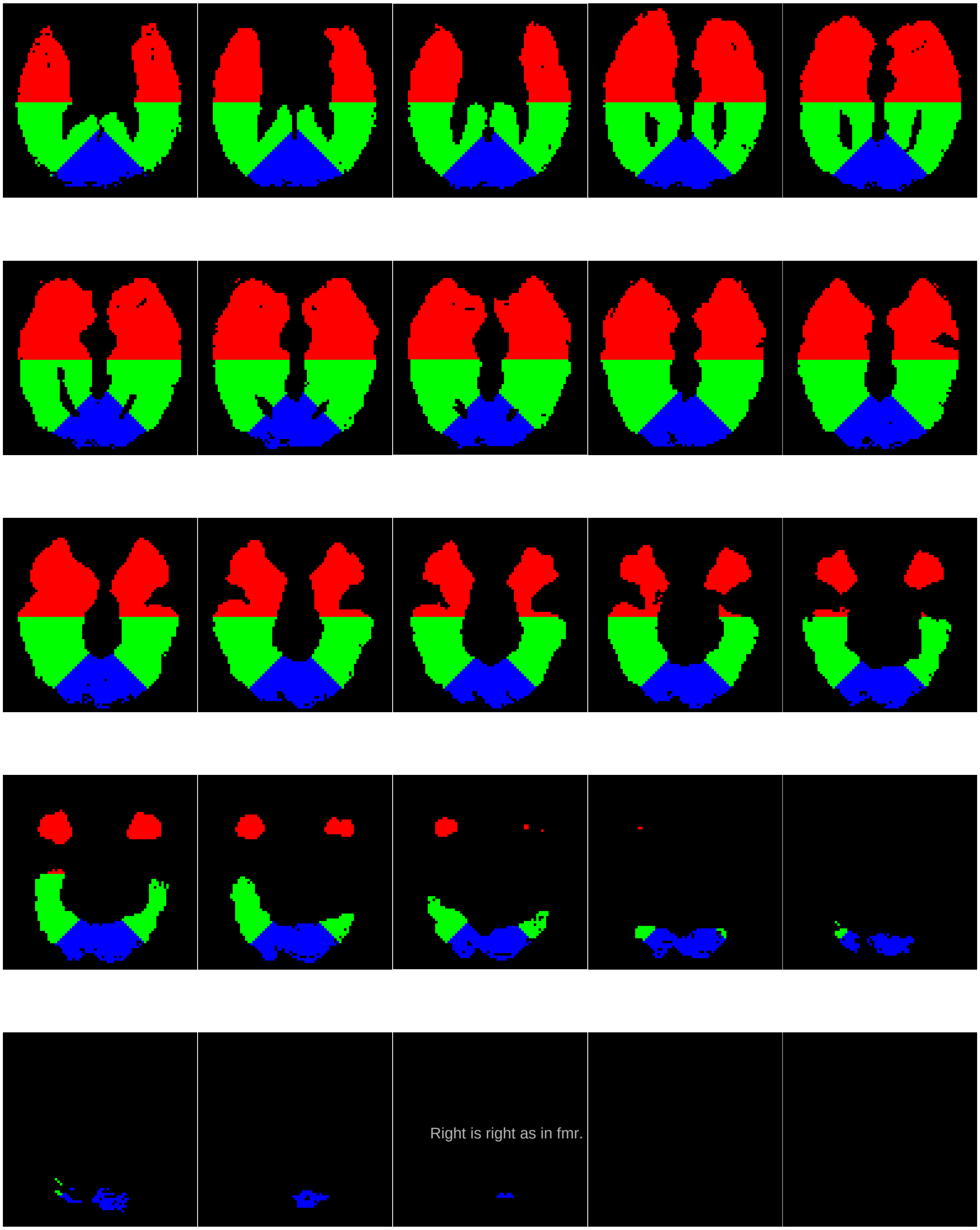

subject 3
session 1

manually defined cortex mask

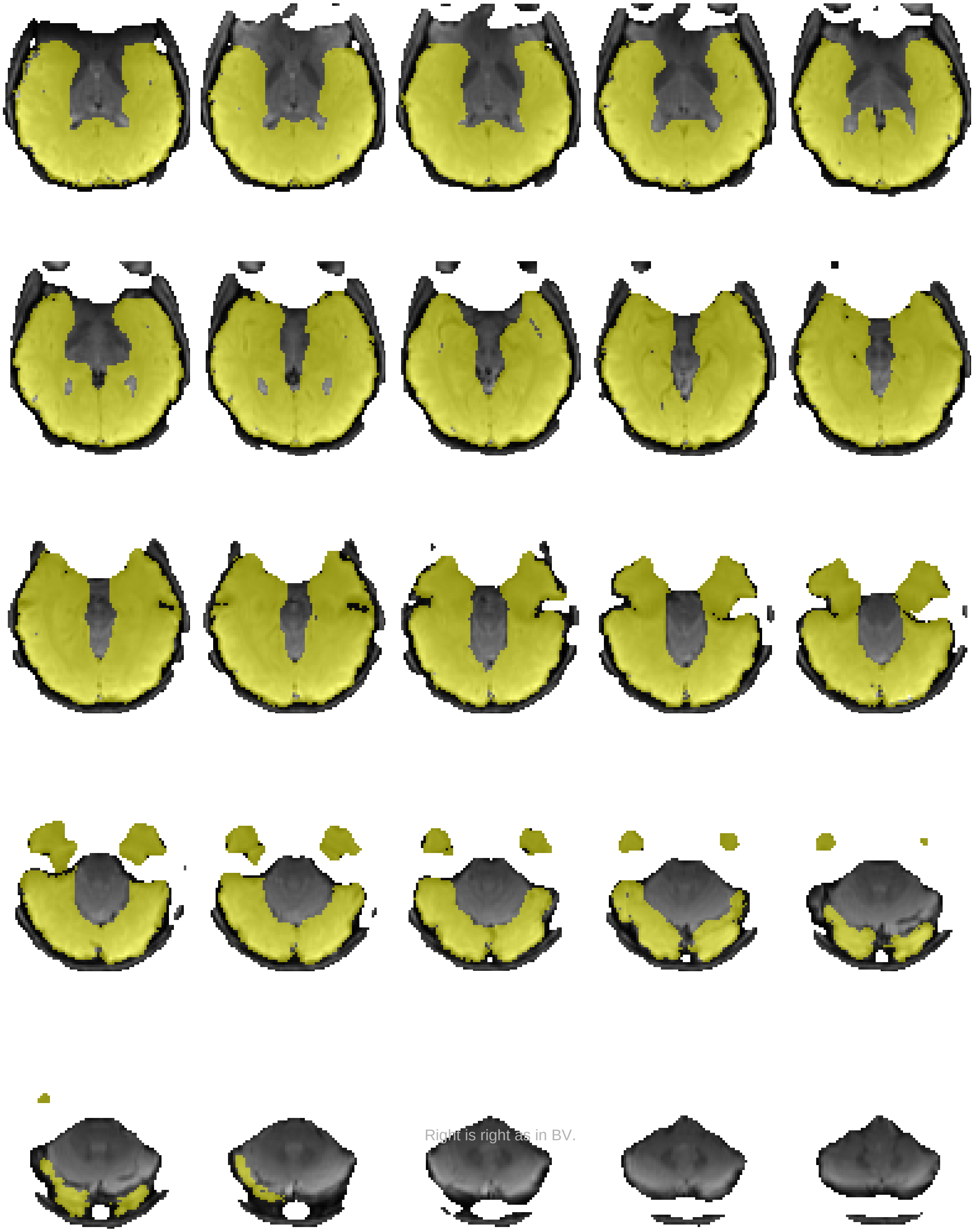

subject 3
session 1

global segmentation: aIT (red), pIT (green), EVA (blue), subcortical (white)

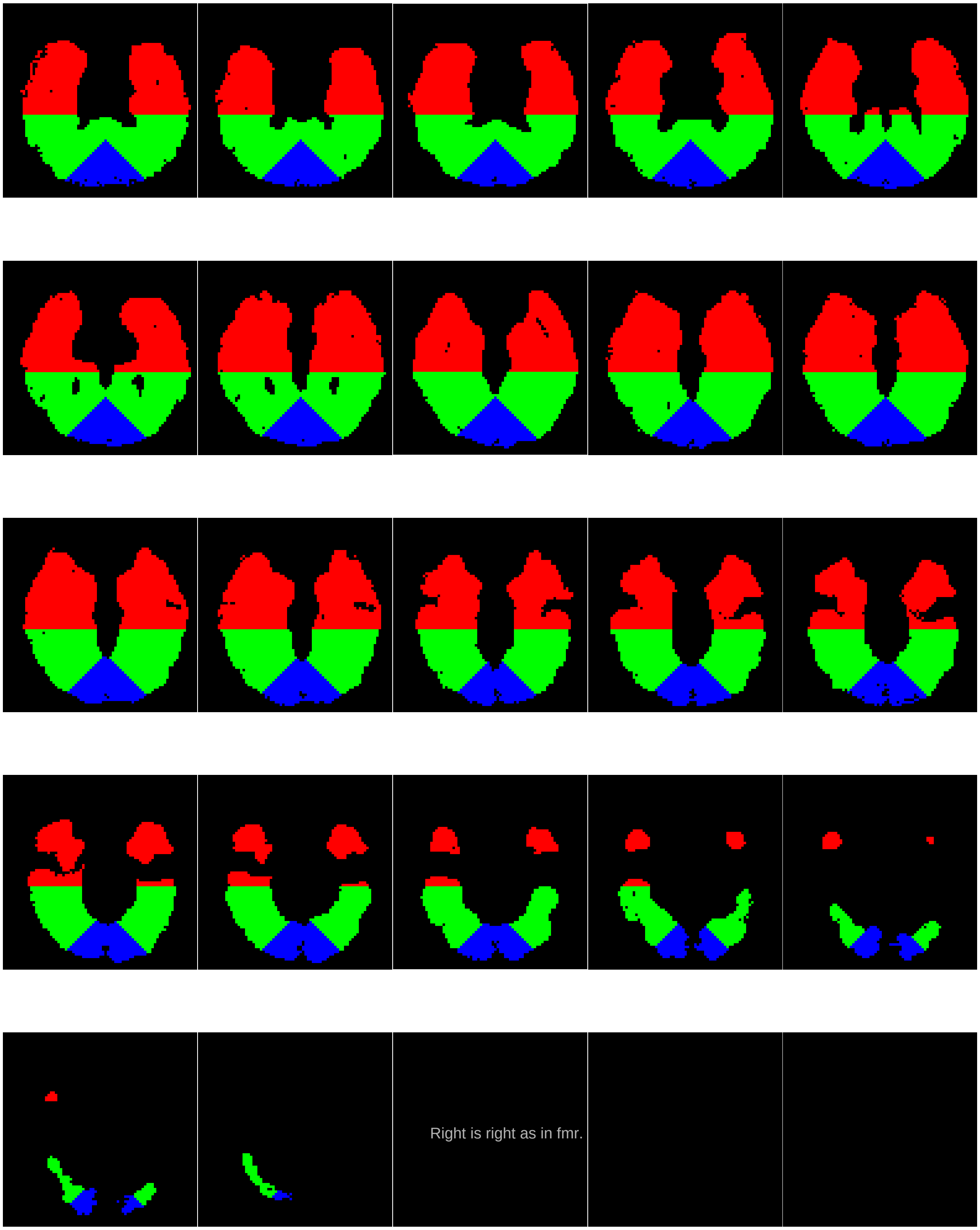

subject 3
session 2

manually defined cortex mask

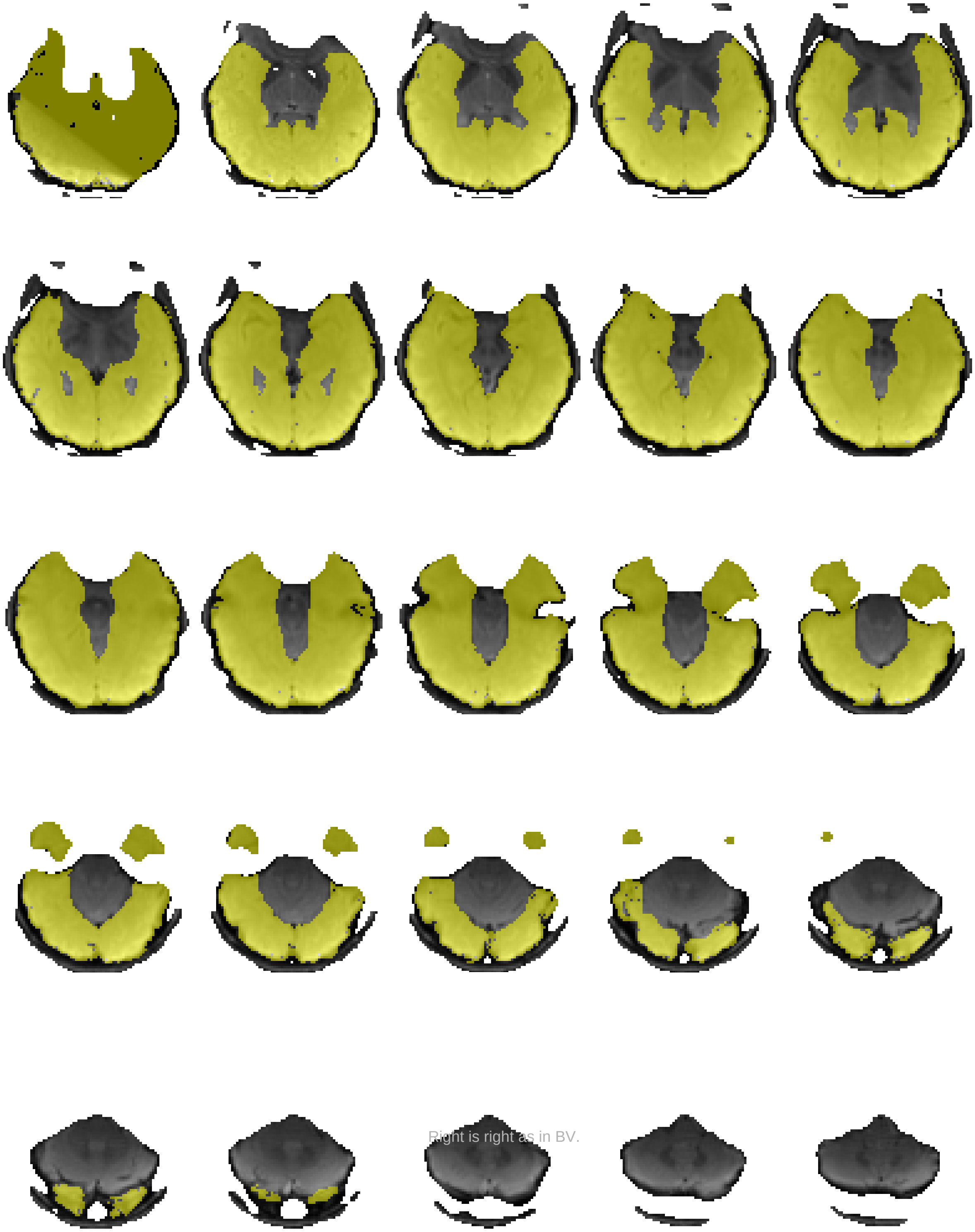

subject 3
session 2

global segmentation: aIT (red), pIT (green), EVA (blue), subcortical (white)

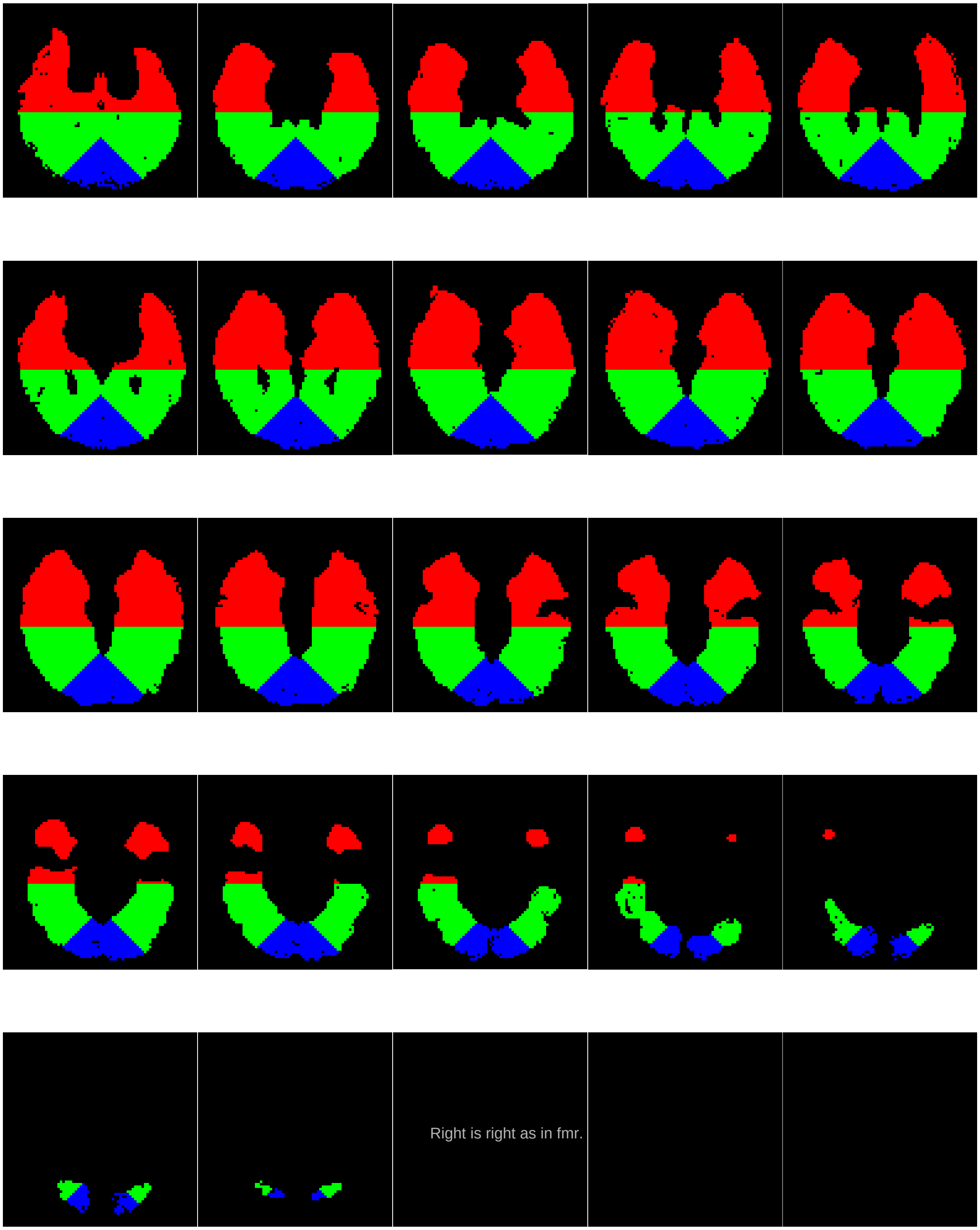

subject 4
session 1

manually defined cortex mask

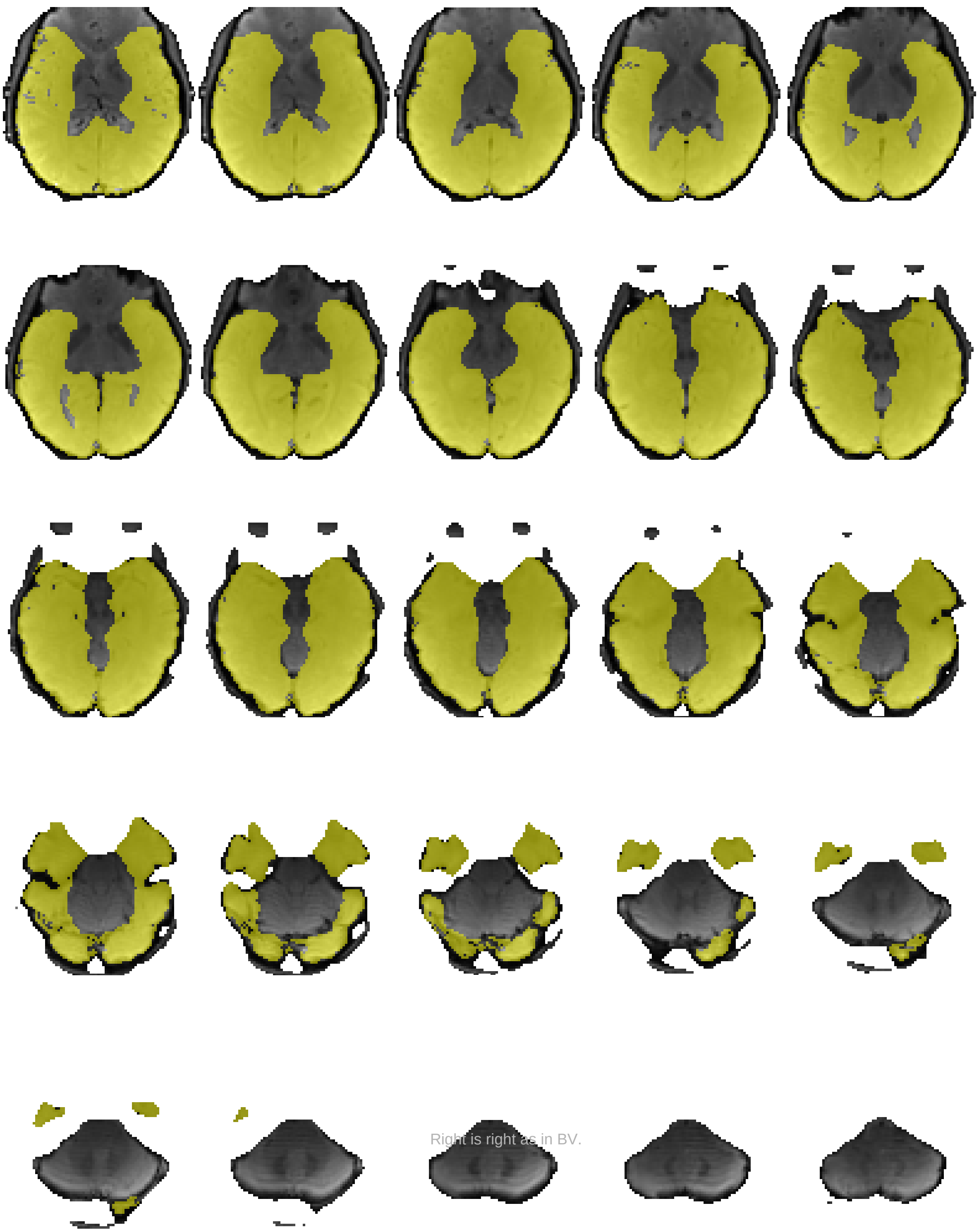

subject 4
session 1

global segmentation: aIT (red), pIT (green), EVA (blue), subcortical (white)

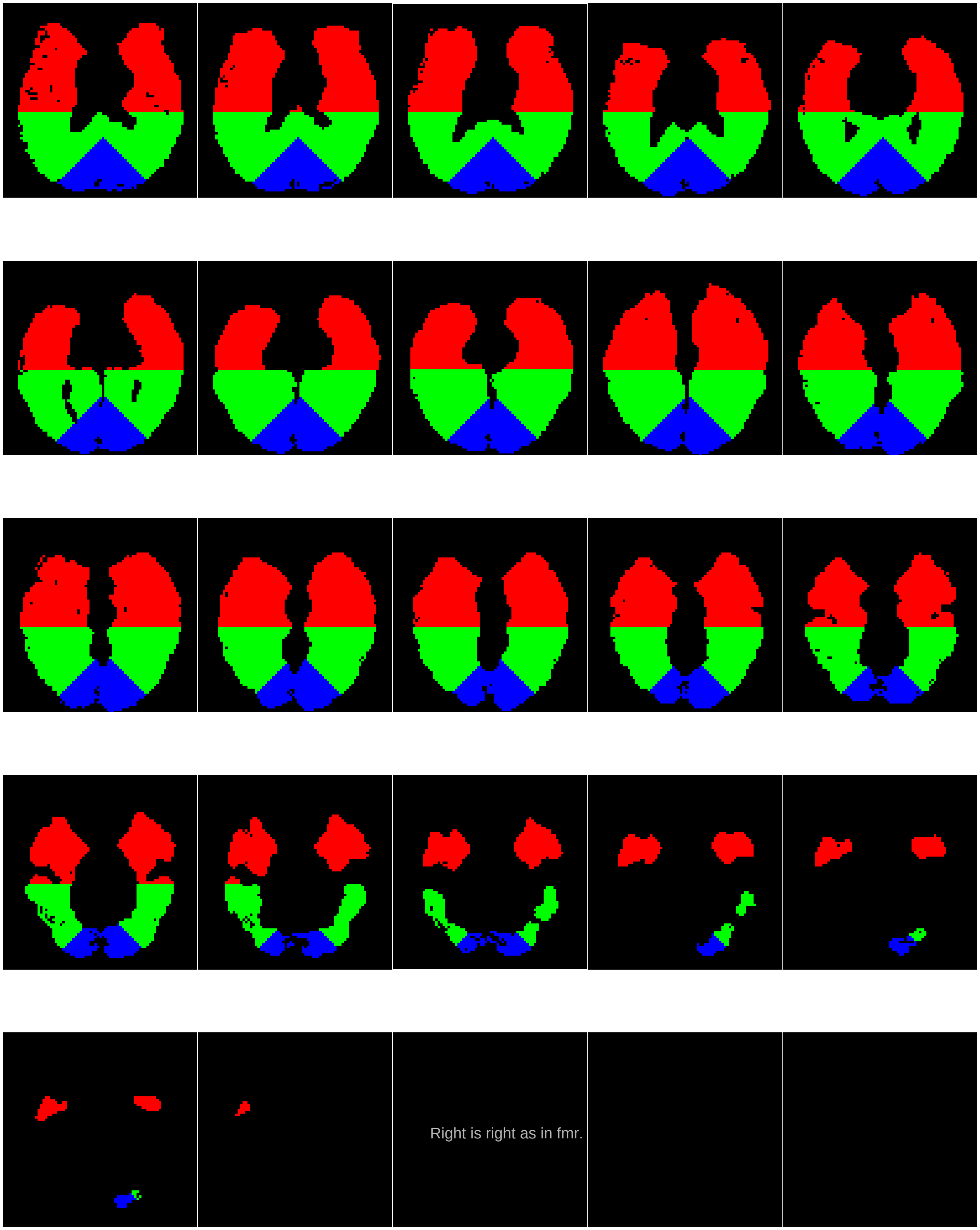

subject 4
session 2

manually defined cortex mask

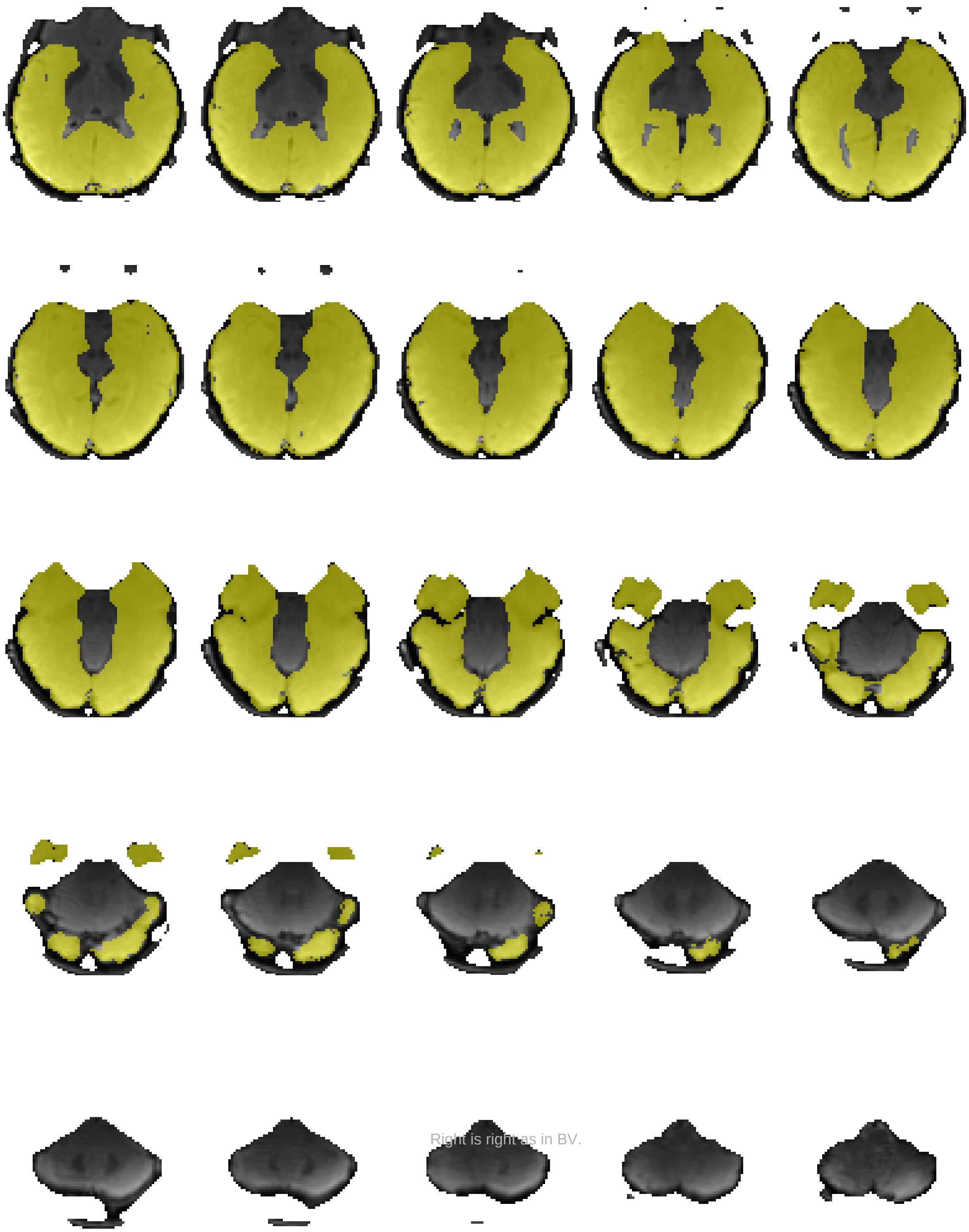

subject 4
session 2

global segmentation: aIT (red), pIT (green), EVA (blue), subcortical (white)

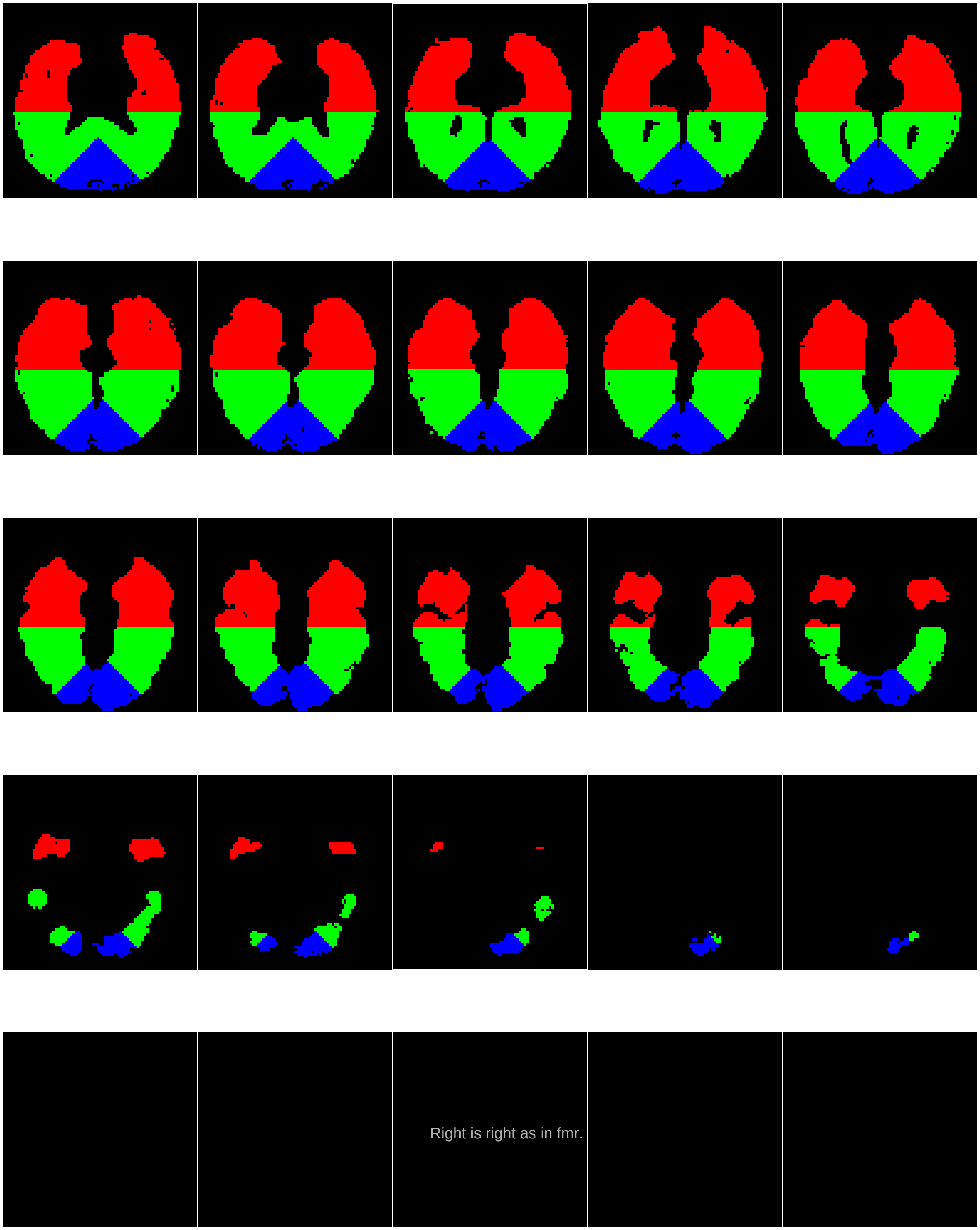

2. Regions of interest

ROI1,1,9,3

subject 1
session 1
EVAvisResp
224 voxels

ROI for EVAvisResp (224)

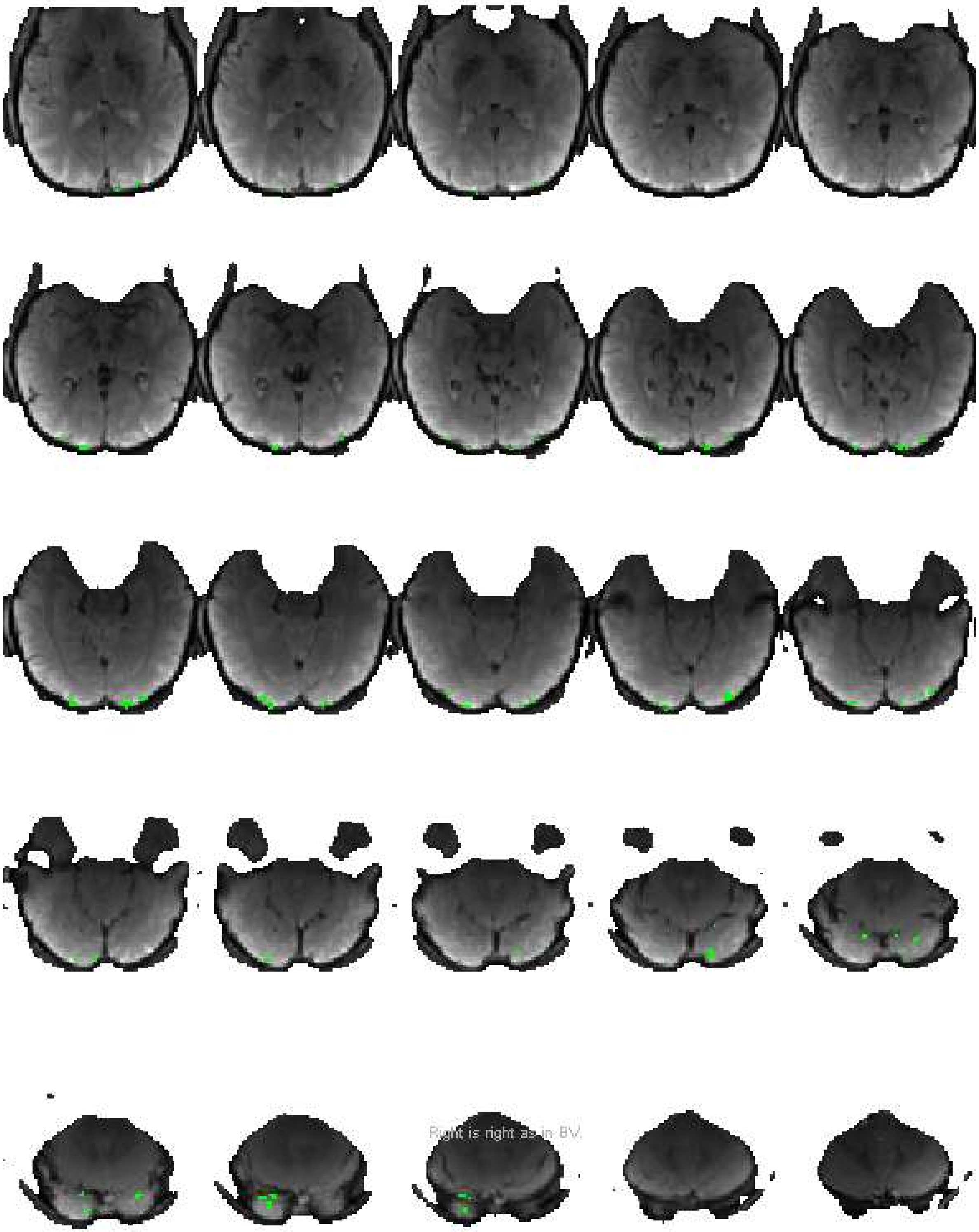

ROI1,2,9,3

subject 1
session 2
EVAvisResp
224 voxels

ROI for EVAvisResp (224)

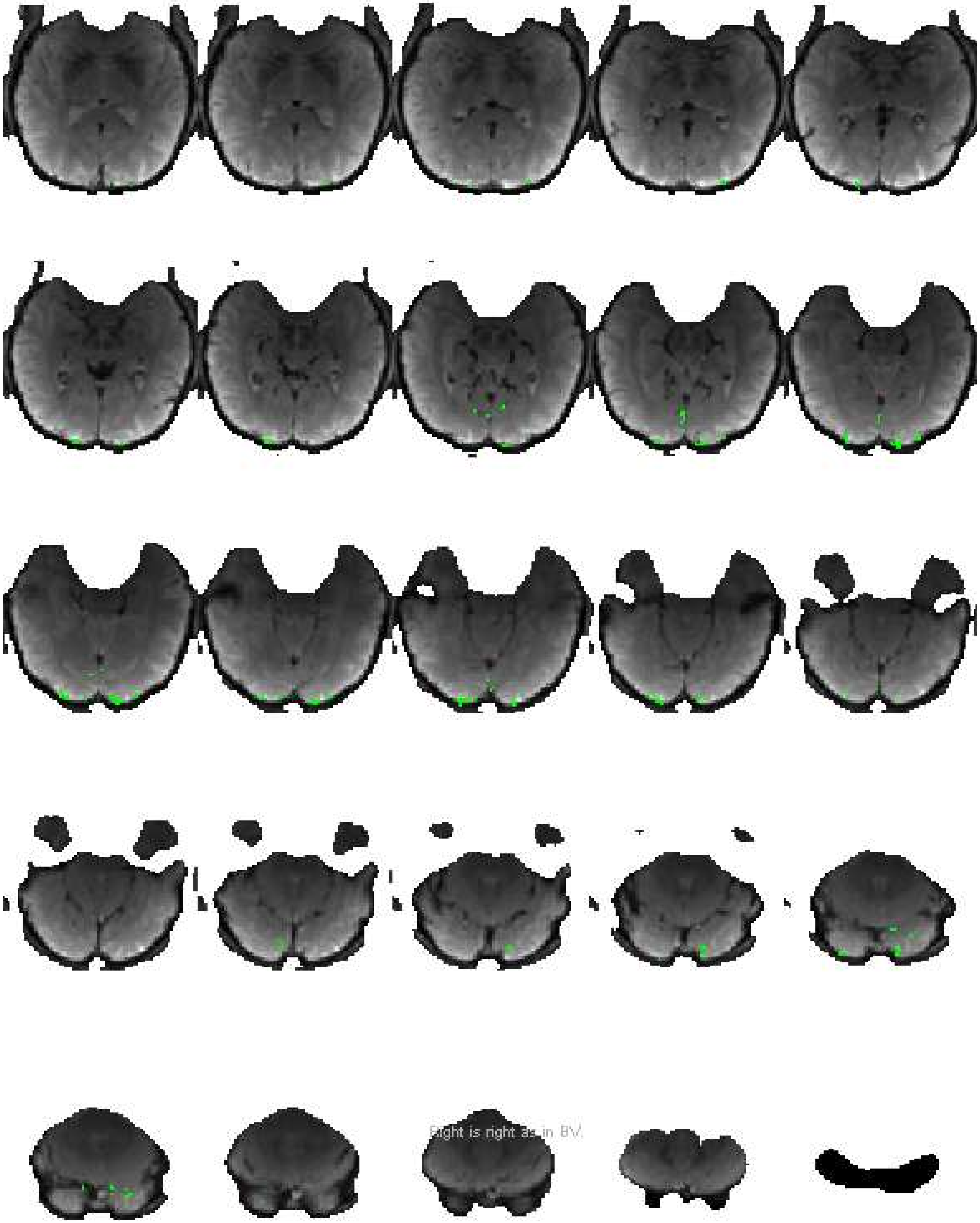

ROI2,1,9,3

subject 2
session 1
EVAvisResp
224 voxels

ROI for EVAvisResp (224)

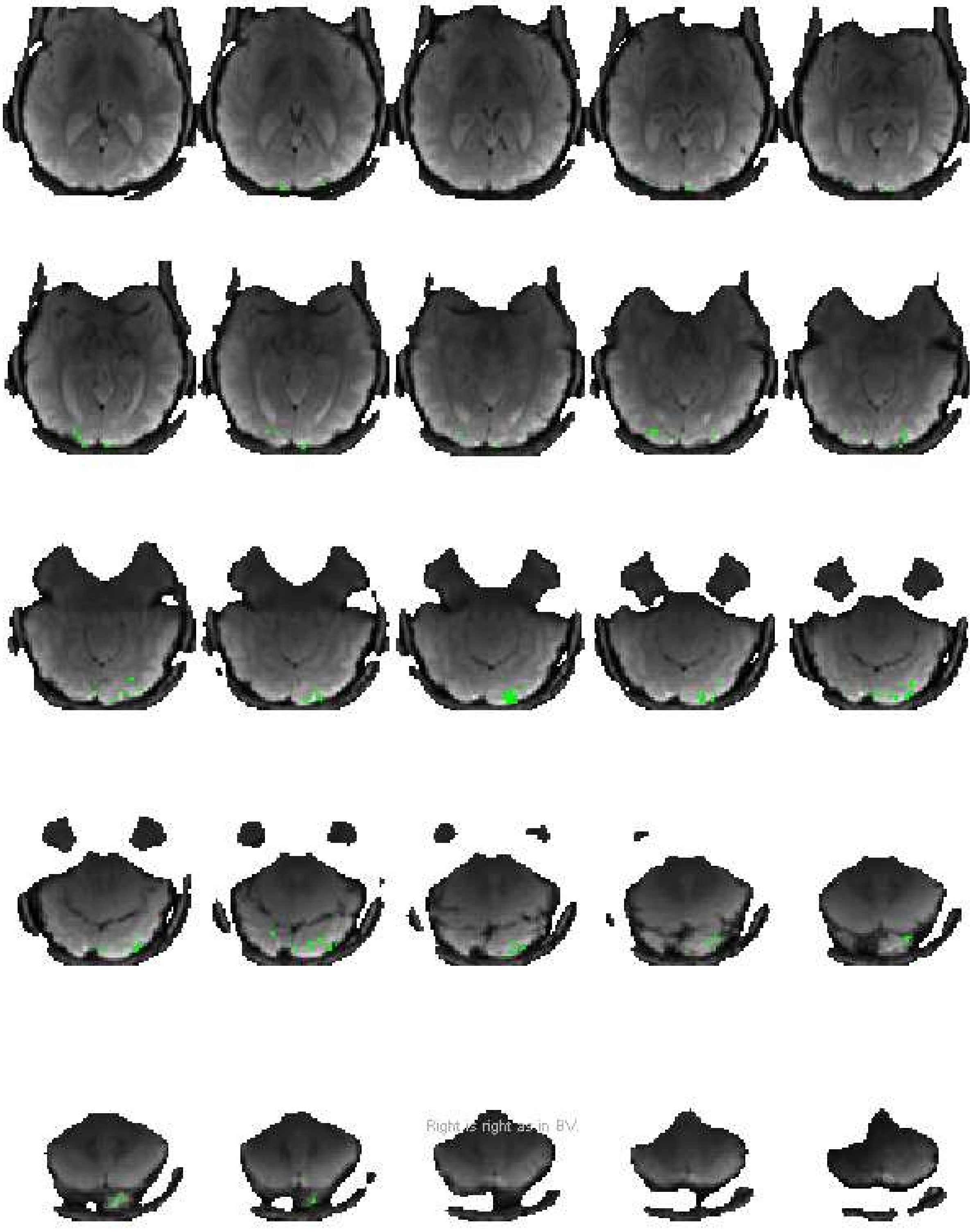

ROI2,3,9,3

subject 2
session 3
EVAvisResp
224 voxels

ROI for EVAvisResp (224)

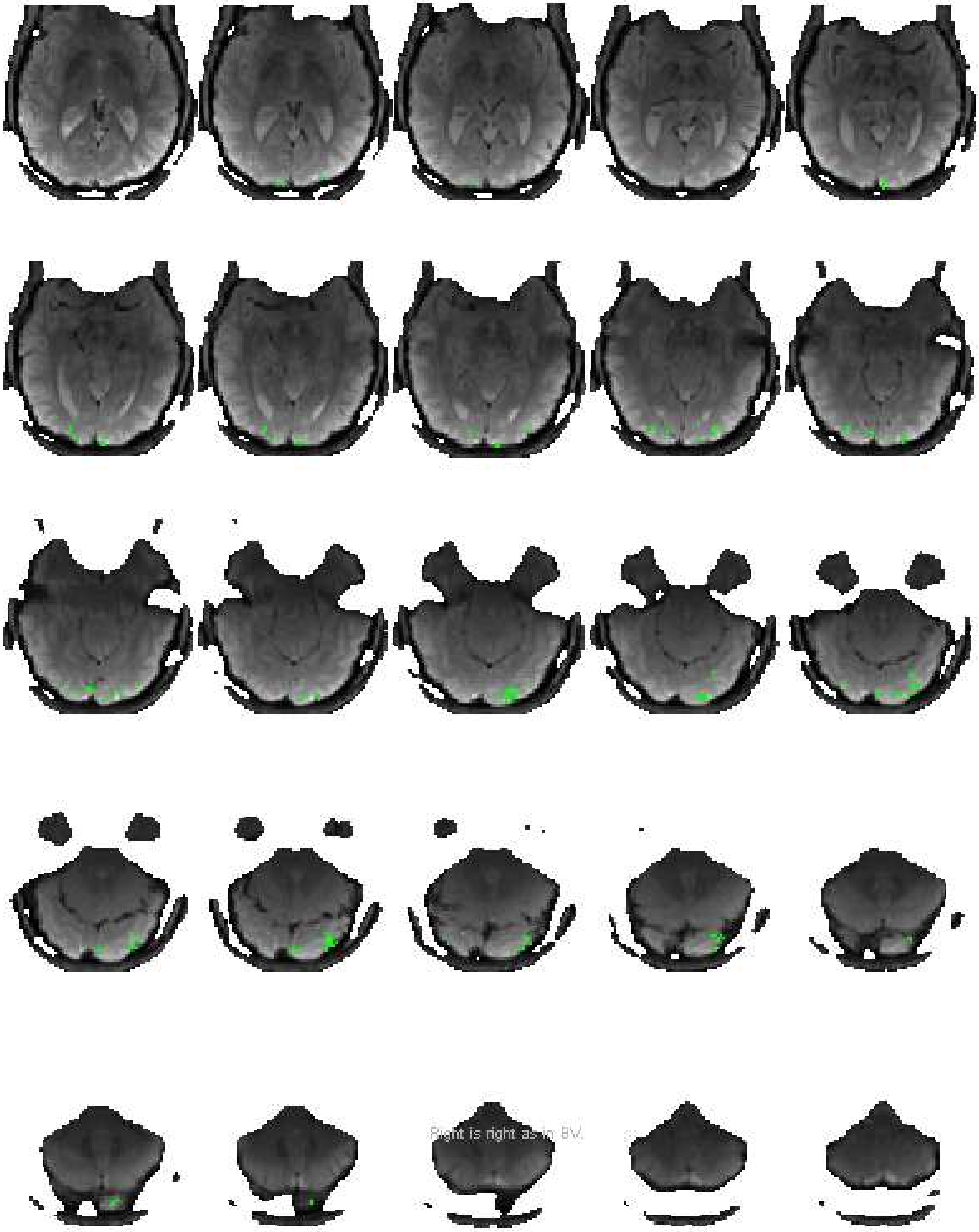

ROI3,1,9,3

subject 3
session 1
EVAvisResp
224 voxels

ROI for EVAvisResp (224)

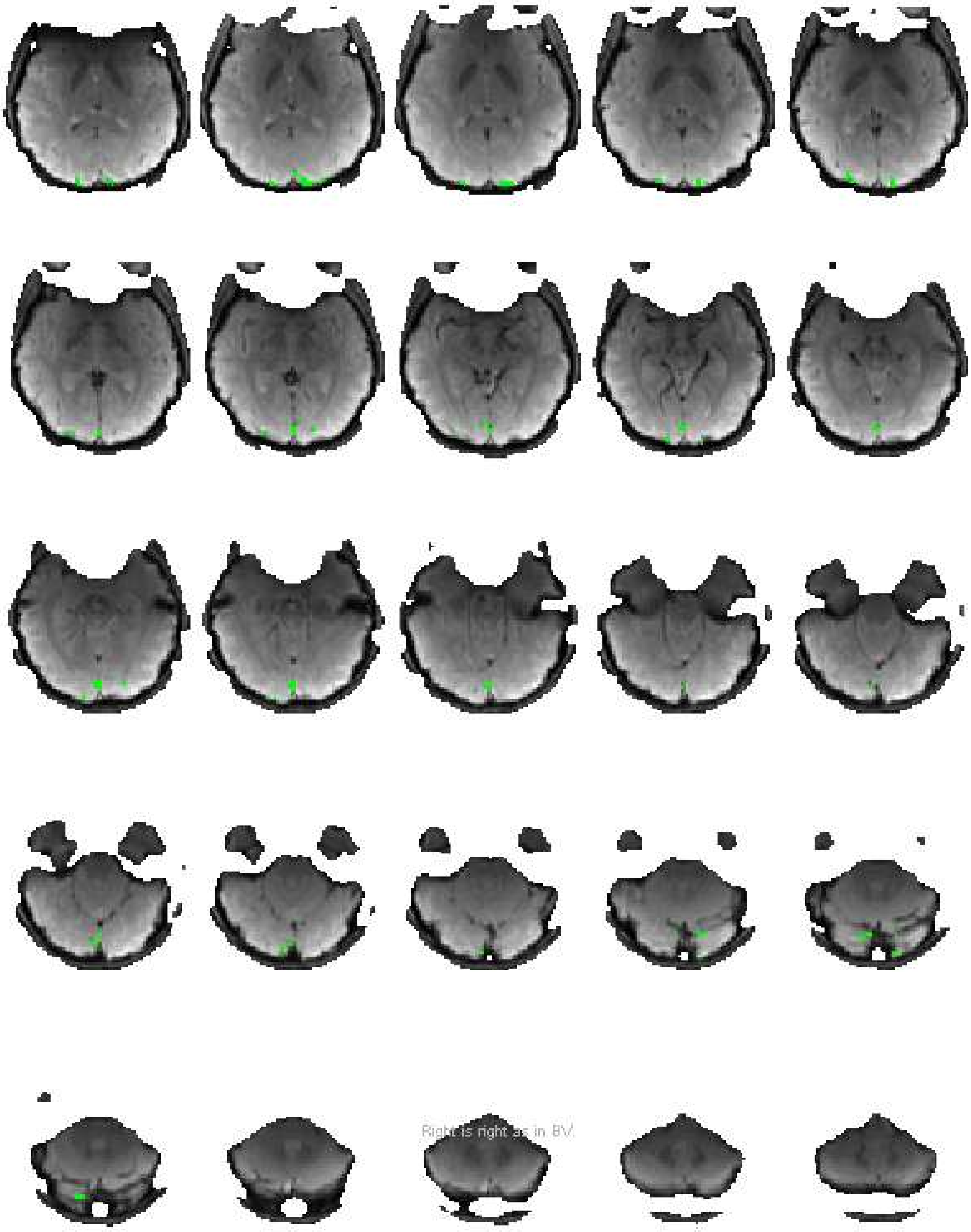

ROI3,2,9,3

subject 3
session 2
EVAvisResp
224 voxels

ROI for EVAvisResp (224)

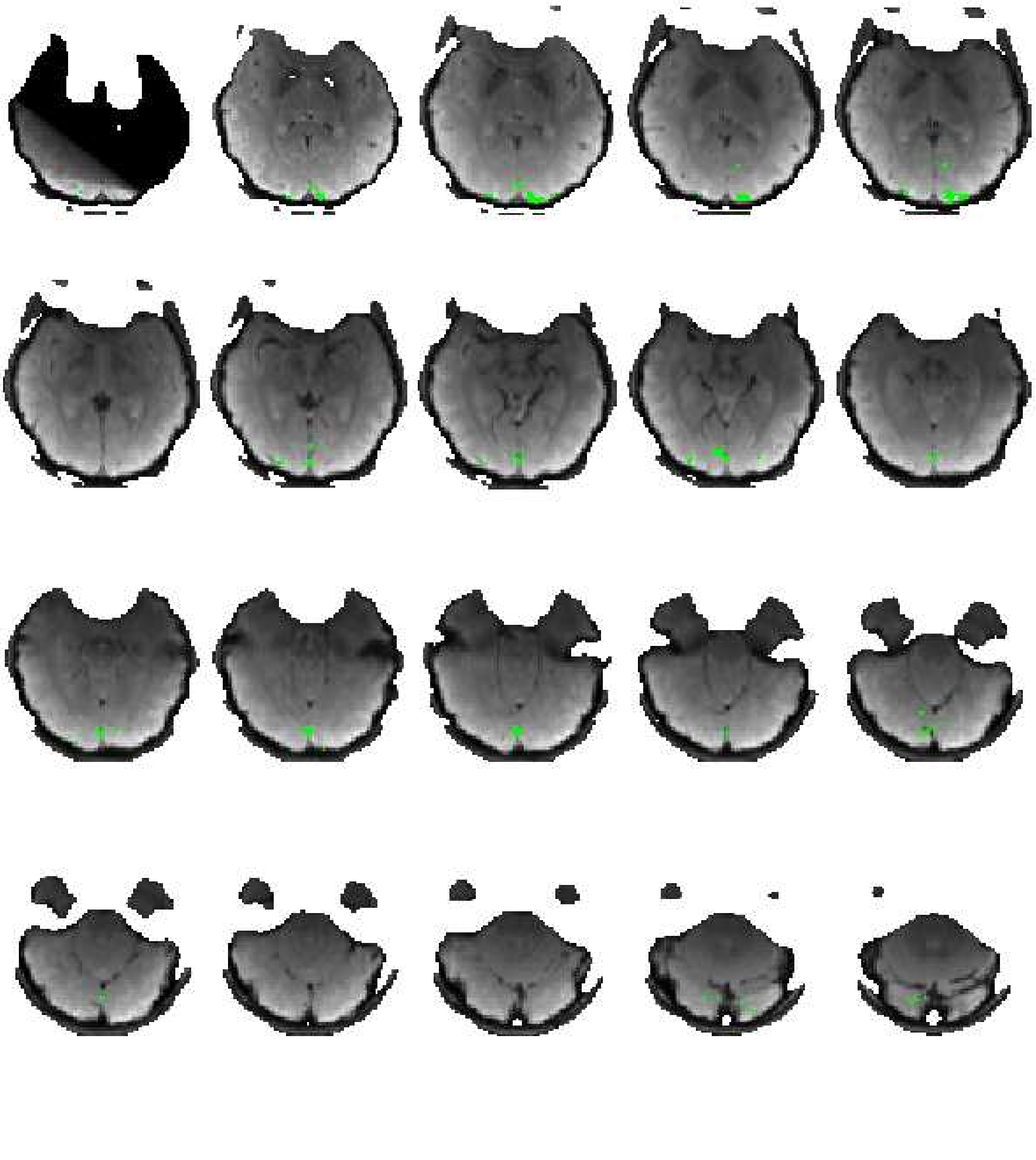

ROI4,1,9,3

subject 4
session 1
EVAvisResp
224 voxels

ROI for EVAvisResp (224)

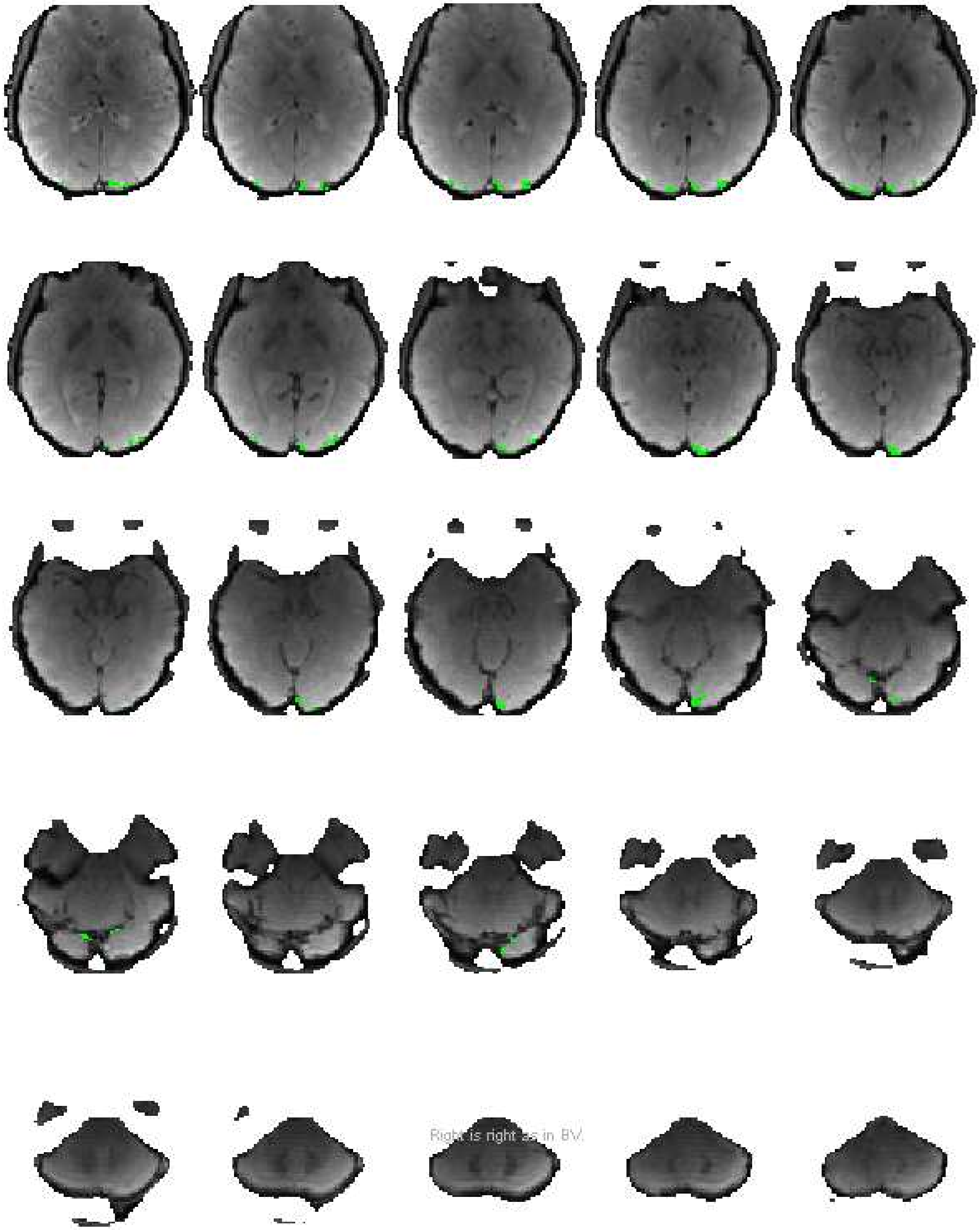

ROI4,2,9,3

subject 4
session 2
EVAvisResp
224 voxels

ROI for EVAvisResp (224)

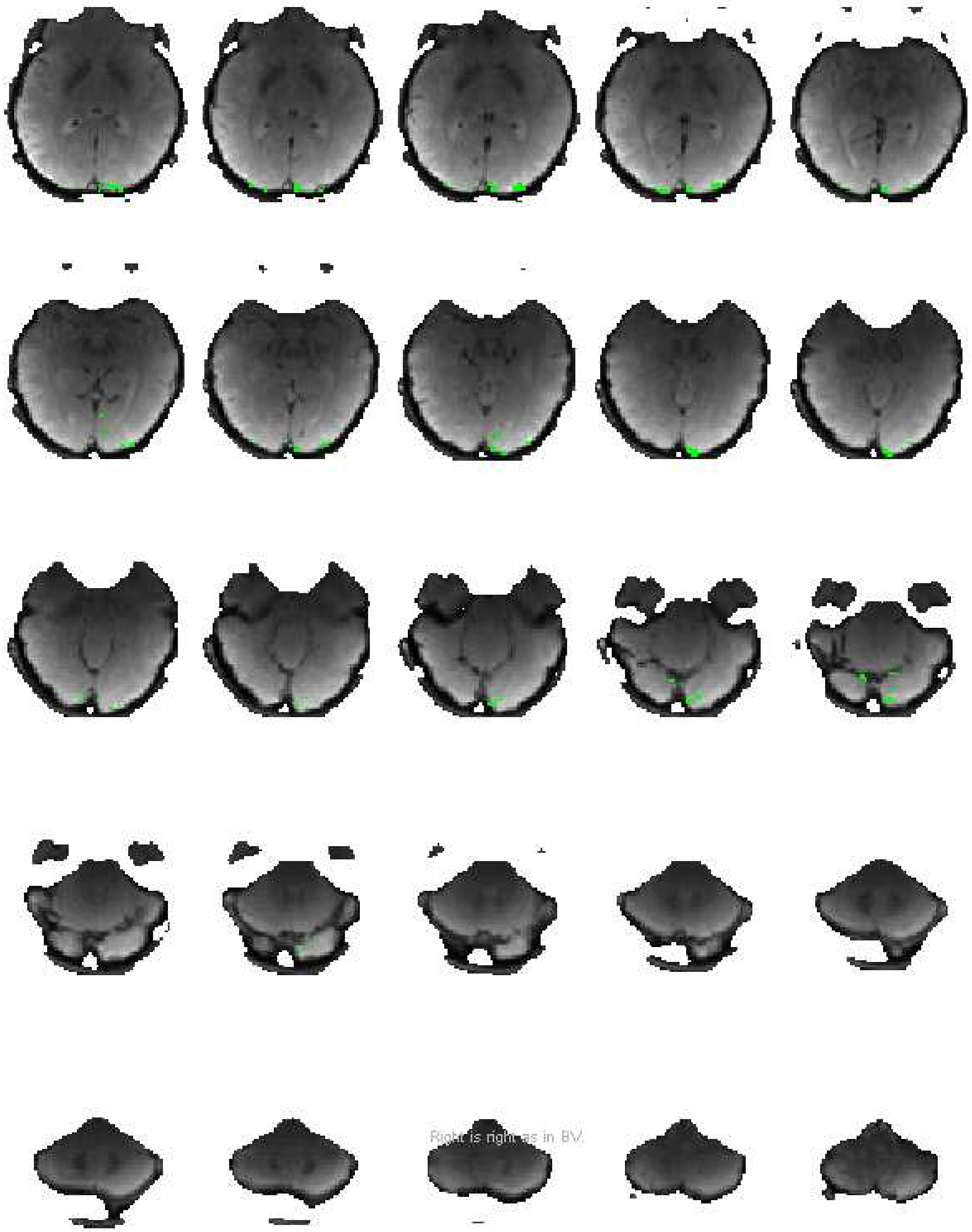

ROI1,1,8,2

subject 1
session 1
hIT without (FFA, PPA)
316 voxels

ROI for hIT without (FFA, PPA) (316)

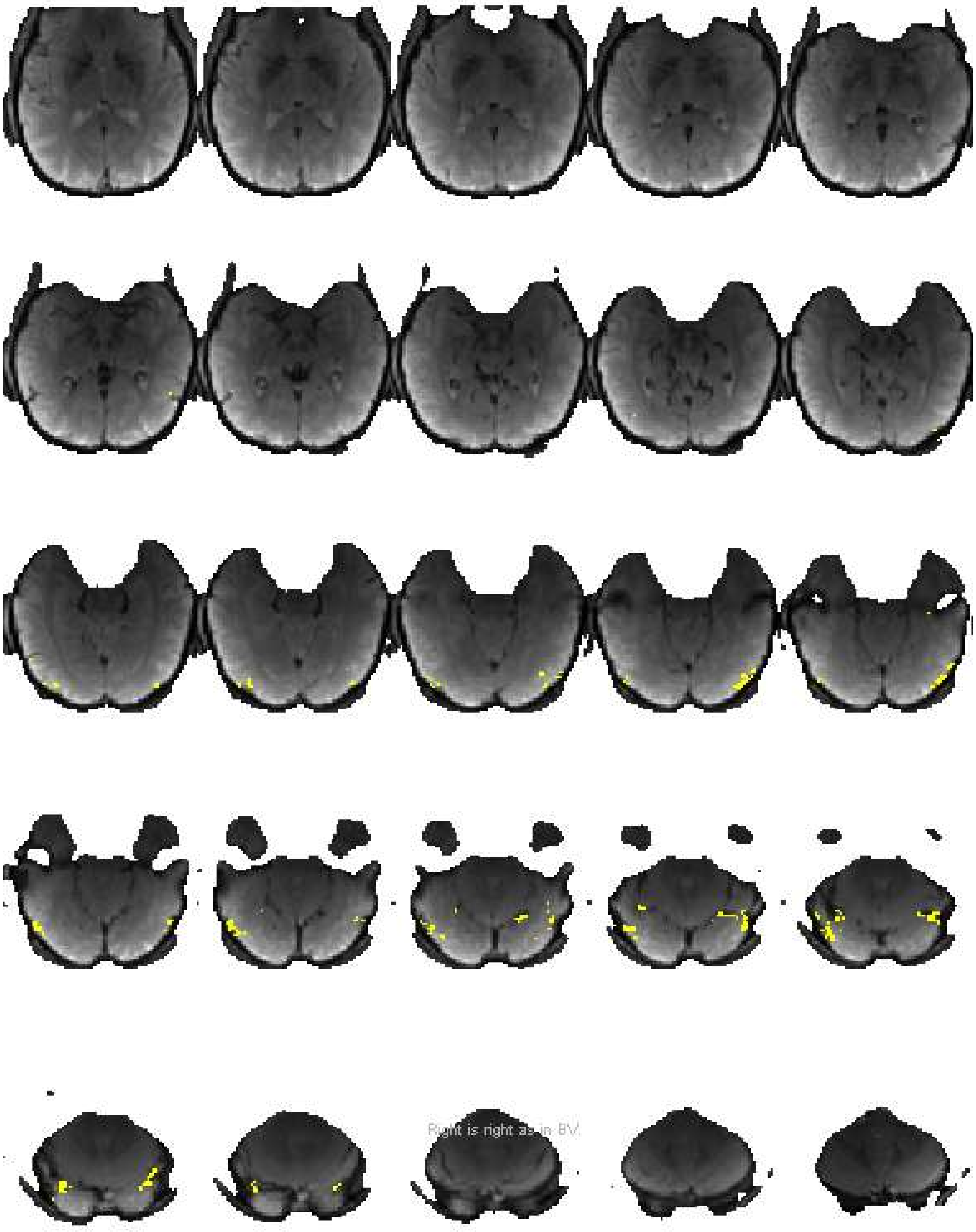

ROI1,2,8,2

subject 1
session 2
hIT without (FFA, PPA)
316 voxels

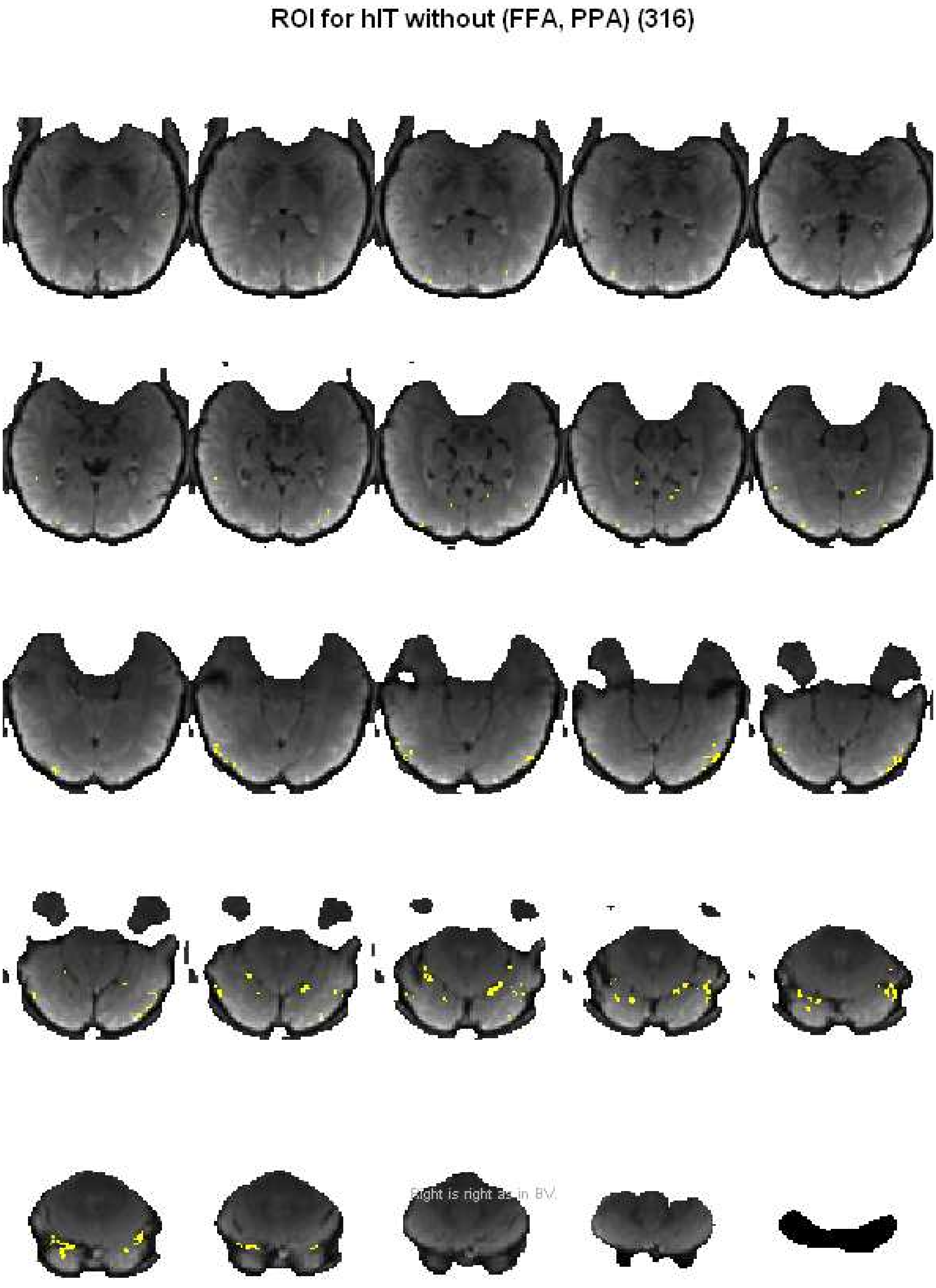

ROI2,1,8,2

subject 2
session 1
hIT without (FFA, PPA)
316 voxels

ROI for hIT without (FFA, PPA) (316)

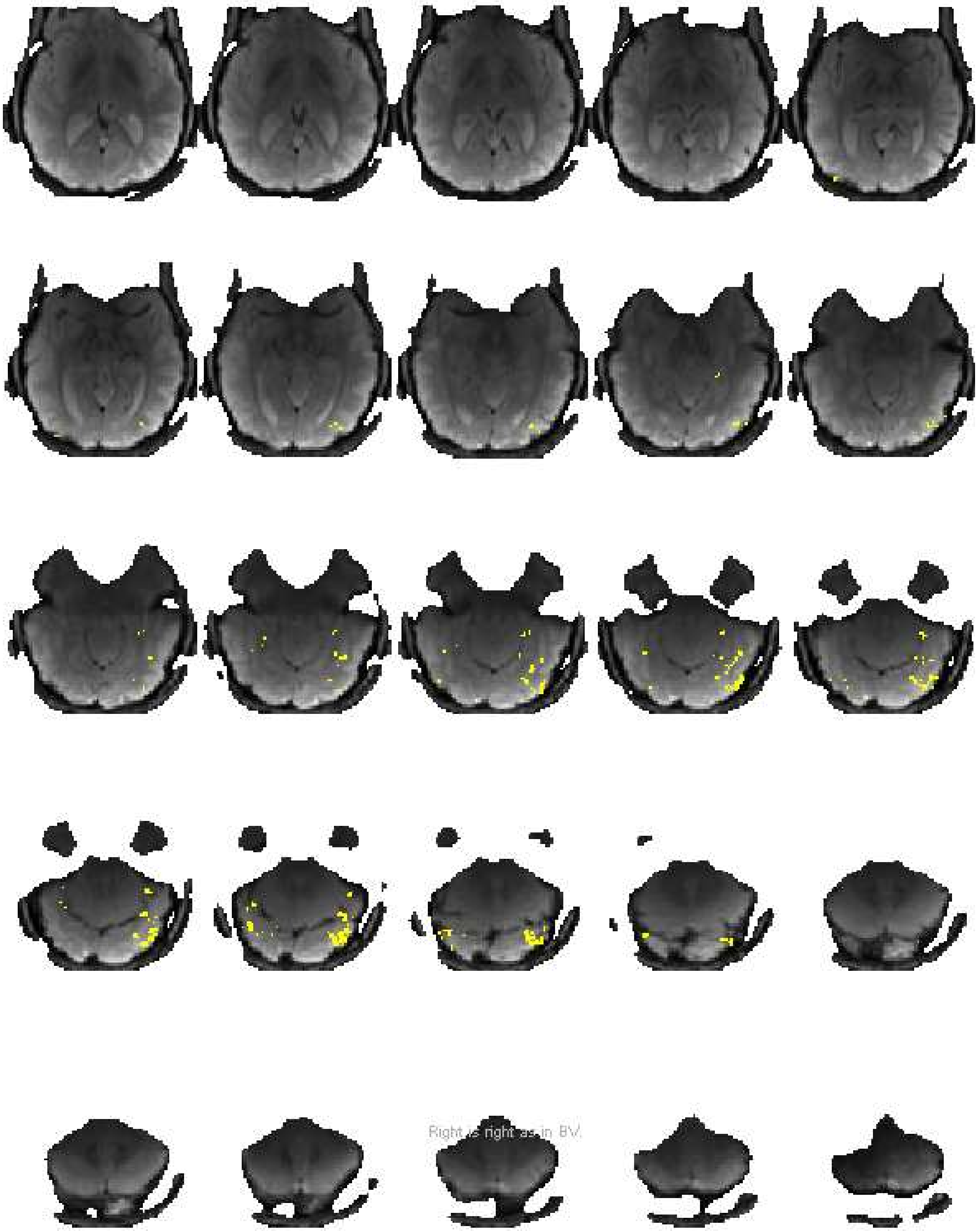

ROI2,3,8,2

subject 2
session 3
hIT without (FFA, PPA)
316 voxels

ROI for hIT without (FFA, PPA) (316)

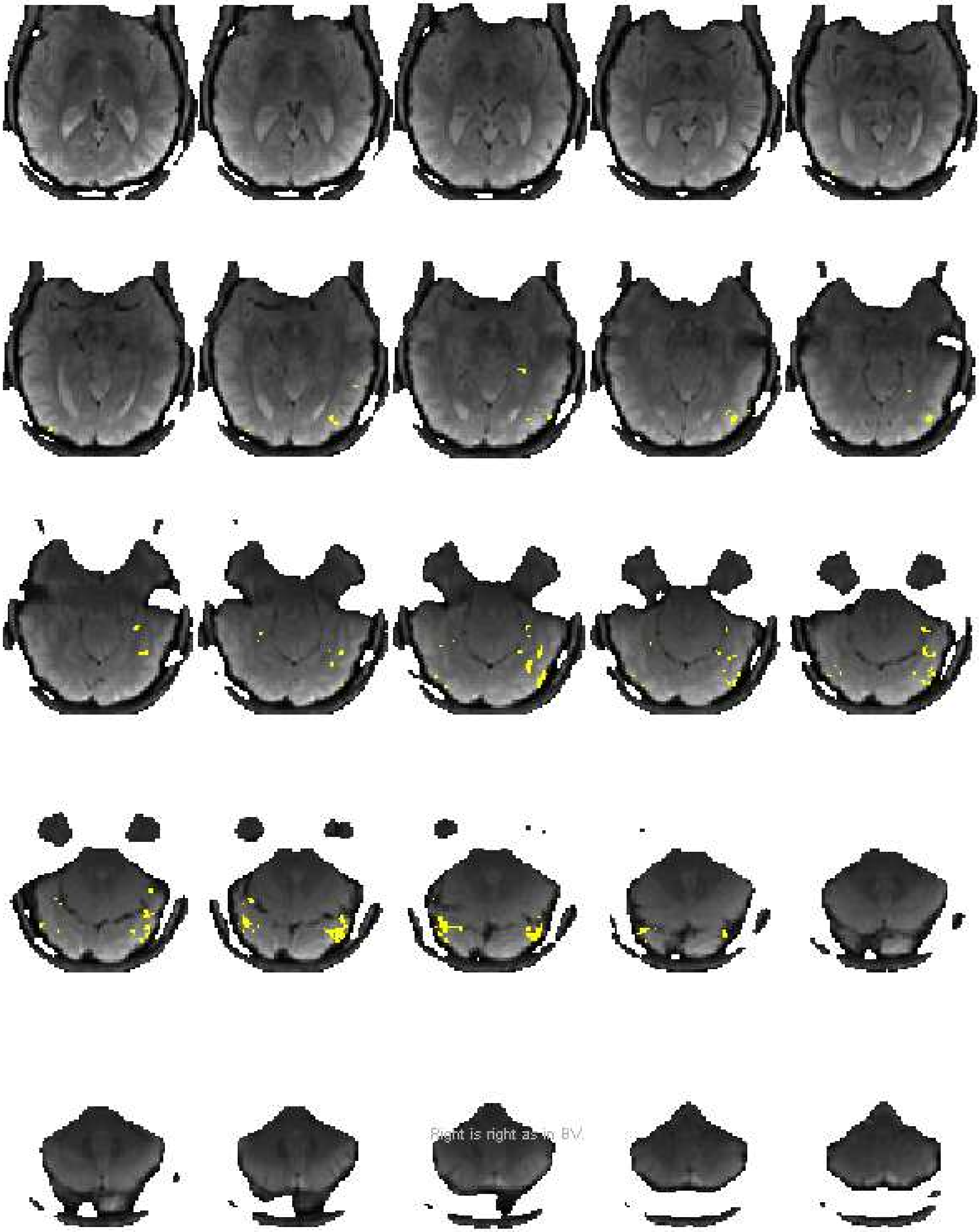

ROI3,1,8,2

subject 3
session 1
hIT without (FFA, PPA)
316 voxels

ROI for hIT without (FFA, PPA) (316)

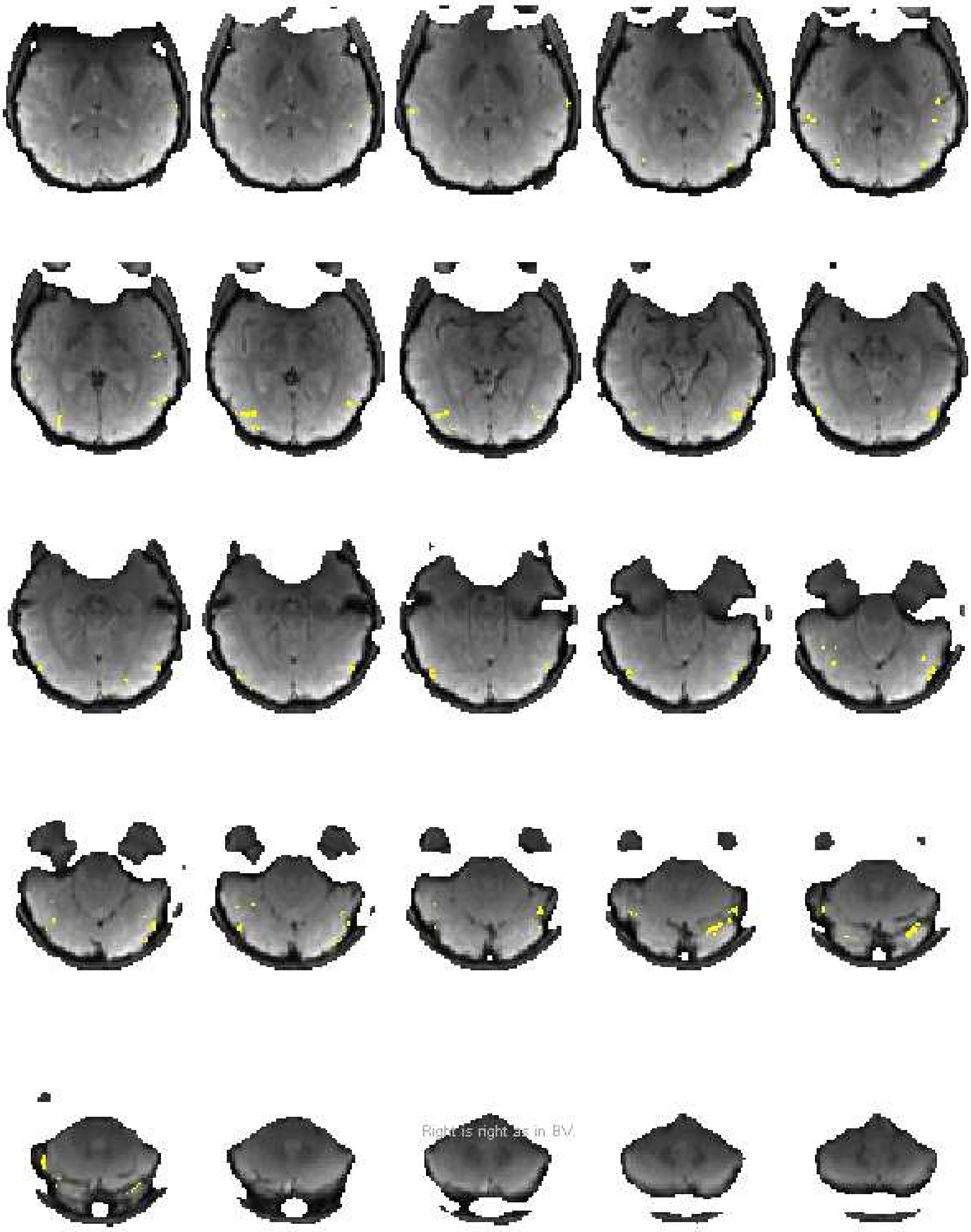

ROI3,2,8,2

subject 3
session 2
hIT without (FFA, PPA)
316 voxels

ROI for hIT without (FFA, PPA) (316)

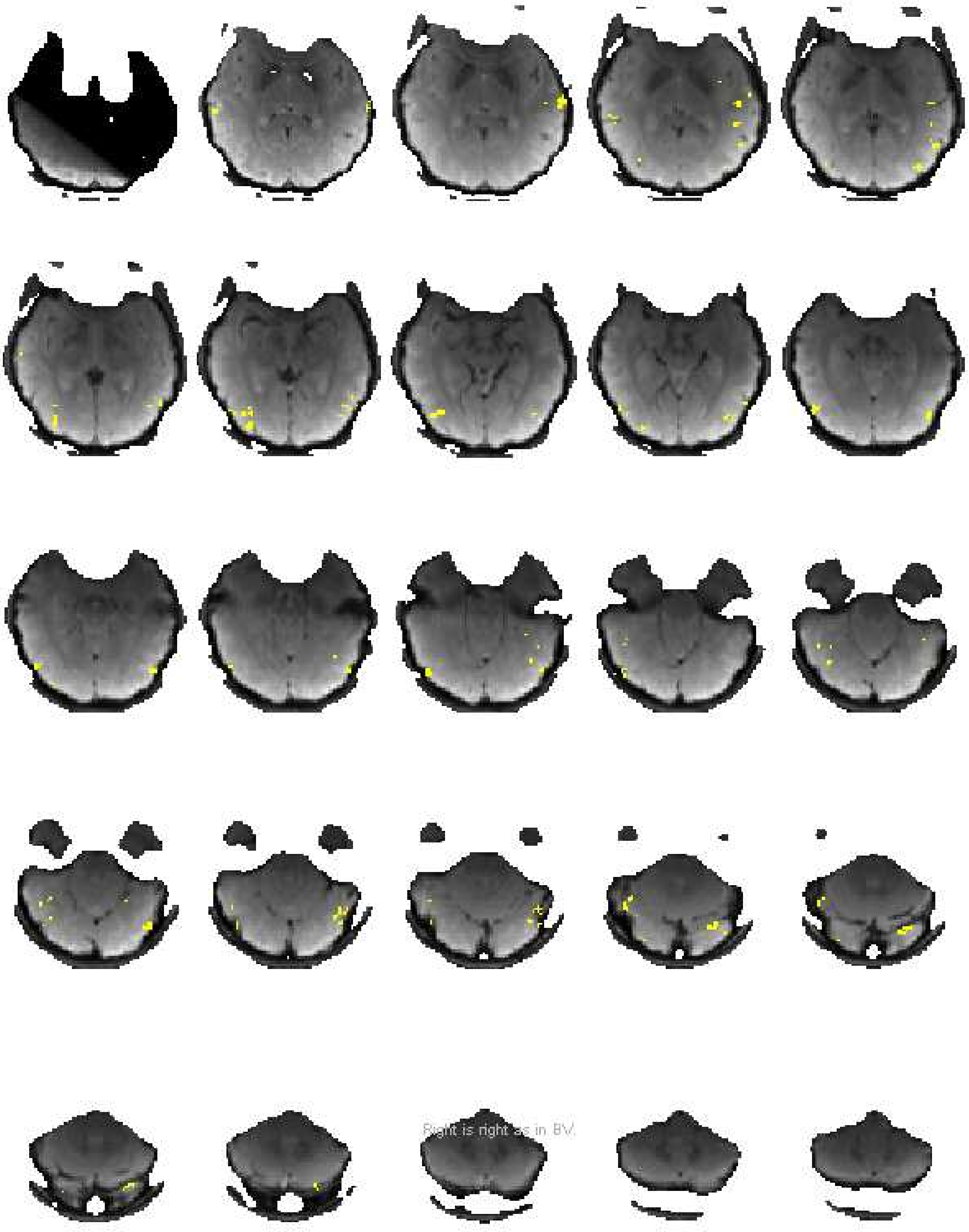

ROI4,1,8,2

subject 4
session 1
hIT without (FFA, PPA)
316 voxels

ROI for hIT without (FFA, PPA) (316)

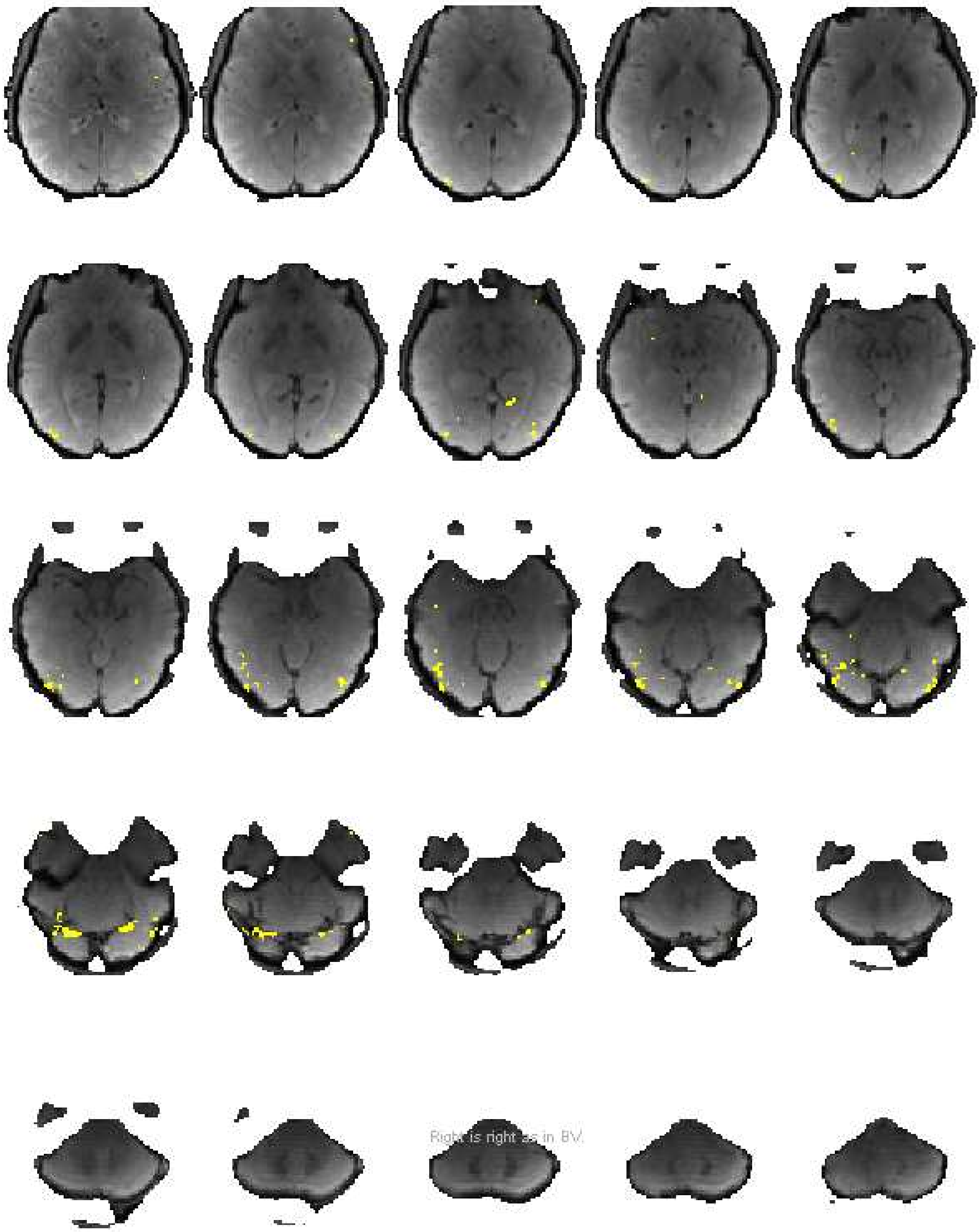

ROI4,2,8,2

subject 4
session 2
hIT without (FFA, PPA)
316 voxels

ROI for hIT without (FFA, PPA) (316)

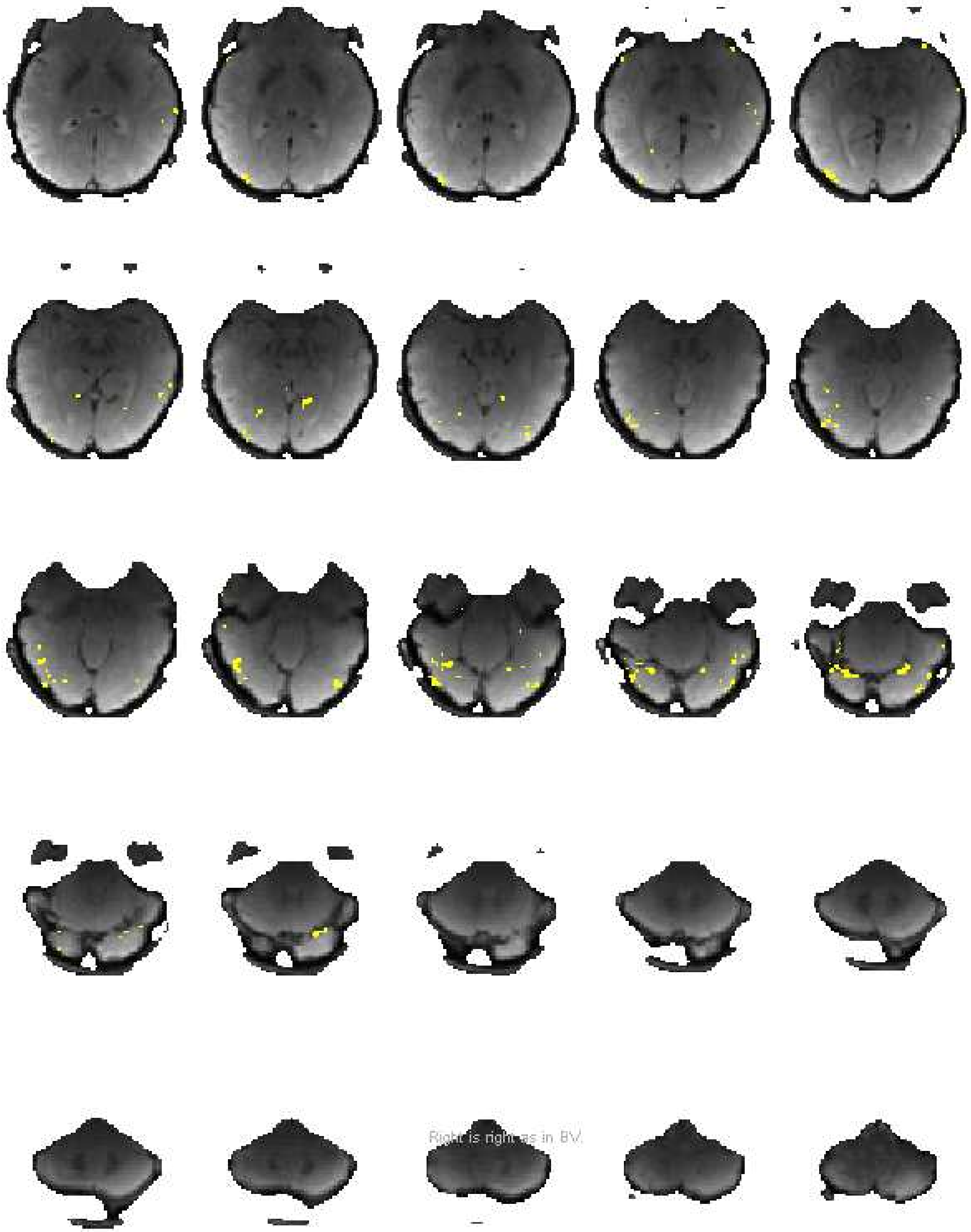

ROI1,1,4,4

subject 1
session 1
FFA_L
128 voxels

ROI for FFA_L (128)

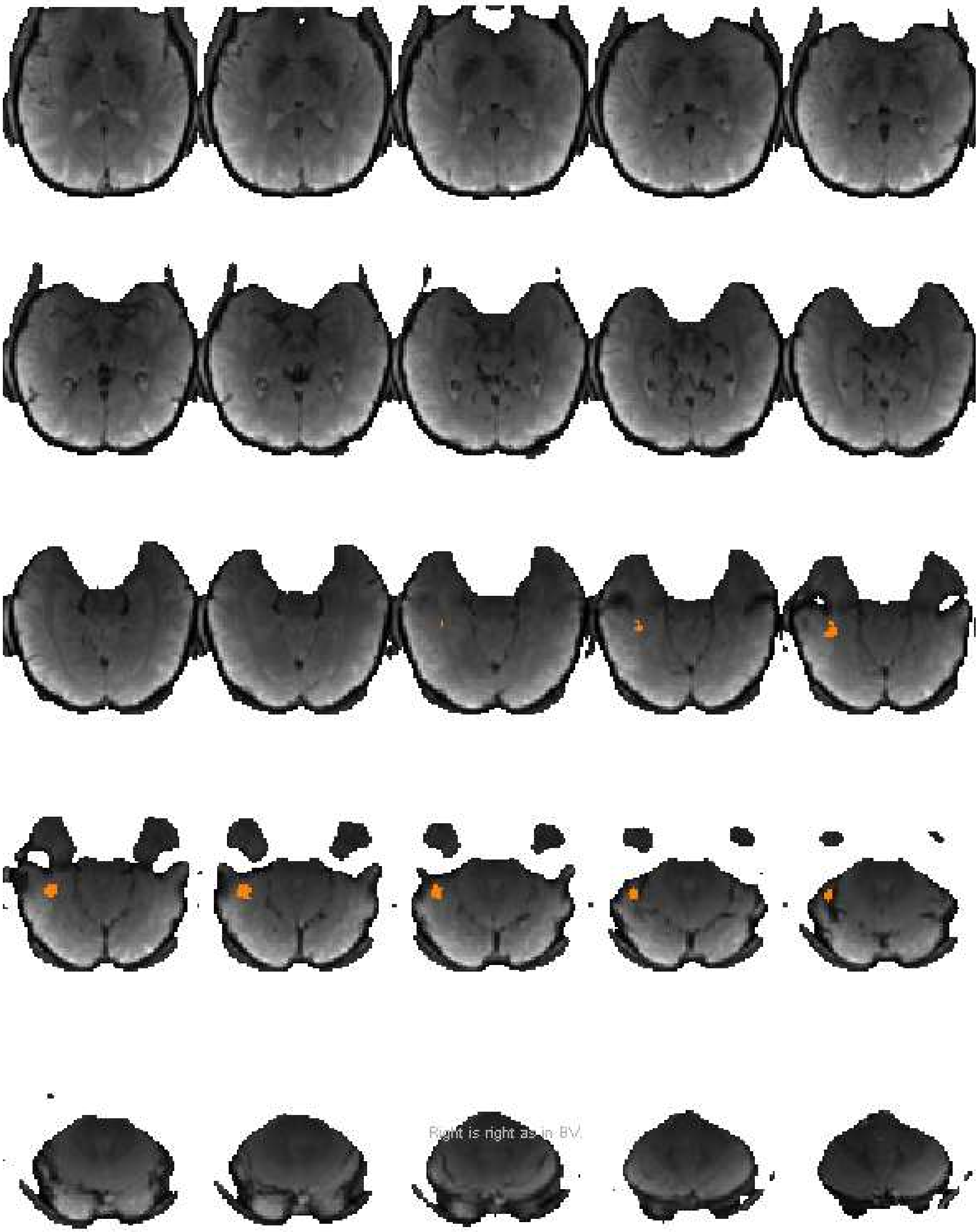

ROI1,2,4,4

subject 1
session 2
FFA_L
128 voxels

ROI for FFA_L (128)

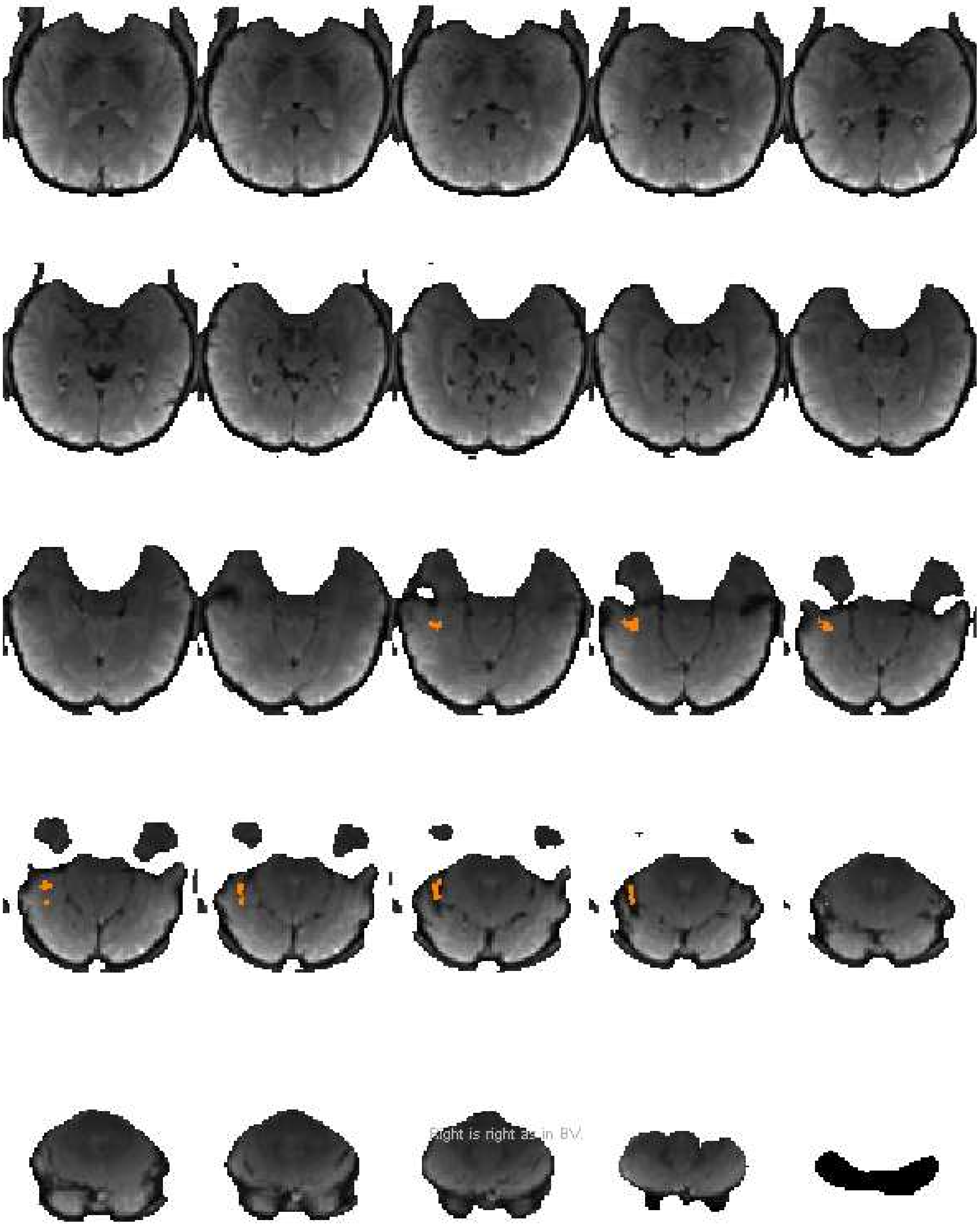

ROI2,1,4,4

subject 2
session 1
FFA_L
128 voxels

ROI for FFA_L (128)

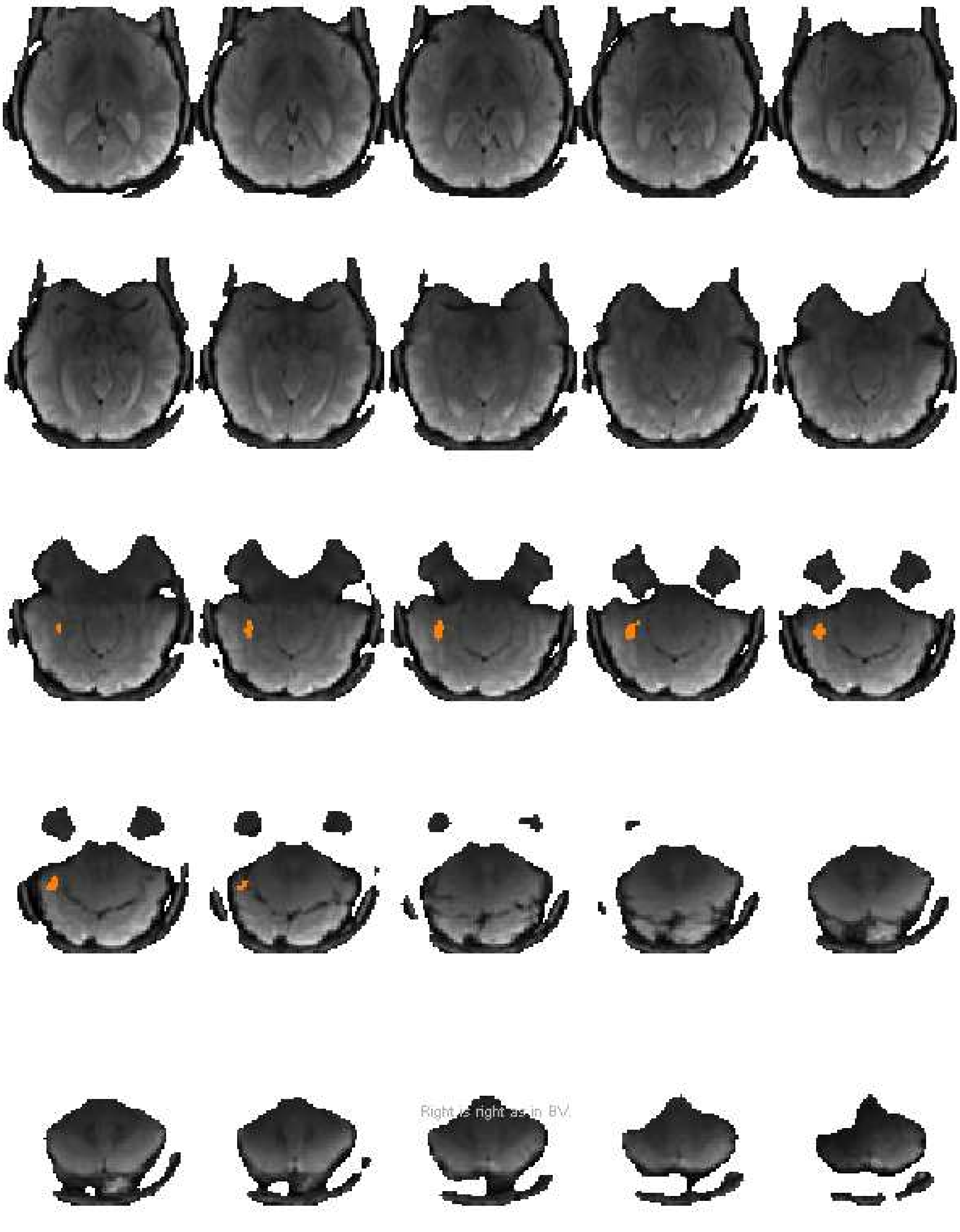

ROI2,3,4,4

subject 2
session 3
FFA_L
128 voxels

ROI for FFA_L (128)

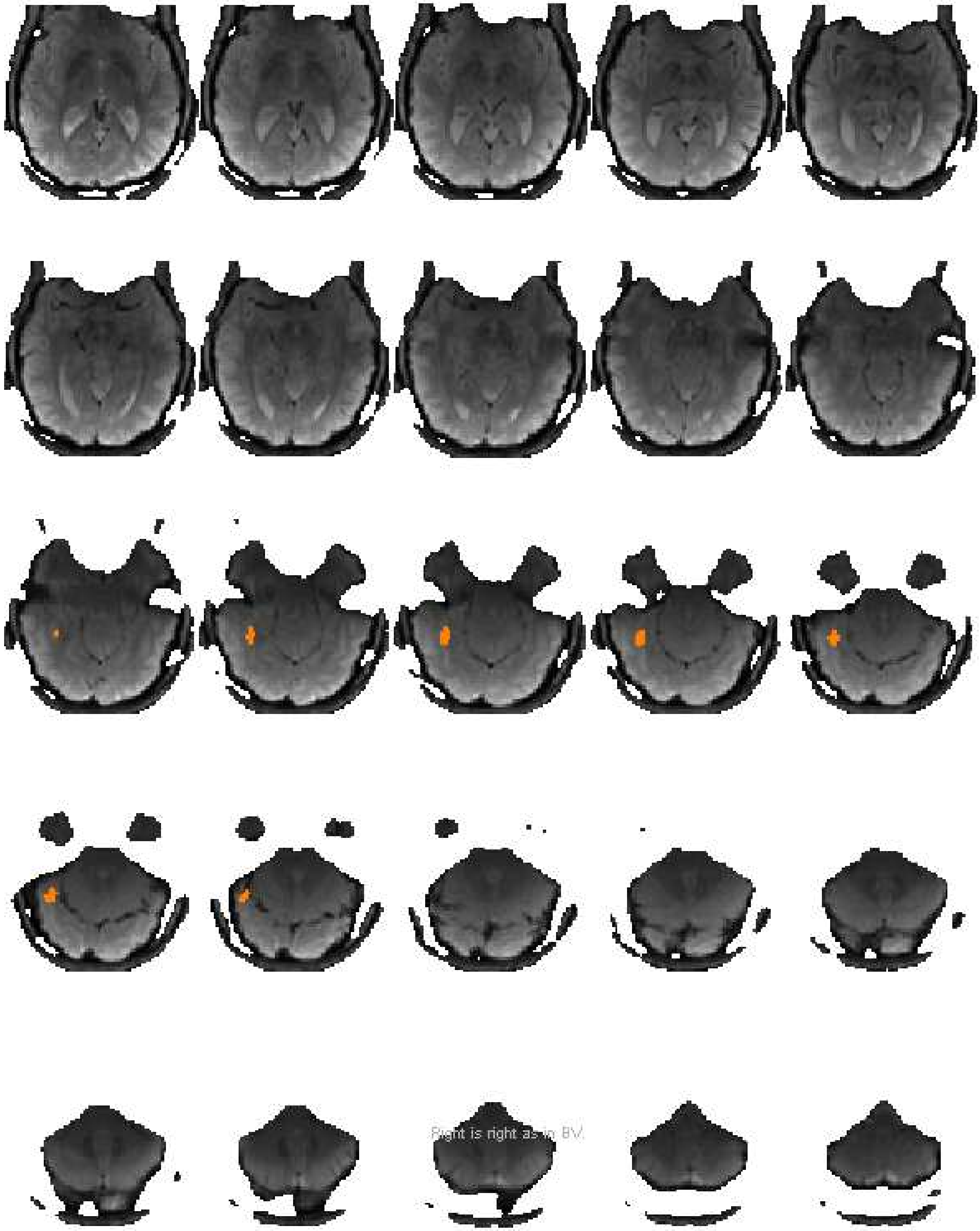

ROI3,1,4,4

subject 3
session 1
FFA_L
128 voxels

ROI for FFA_L (128)

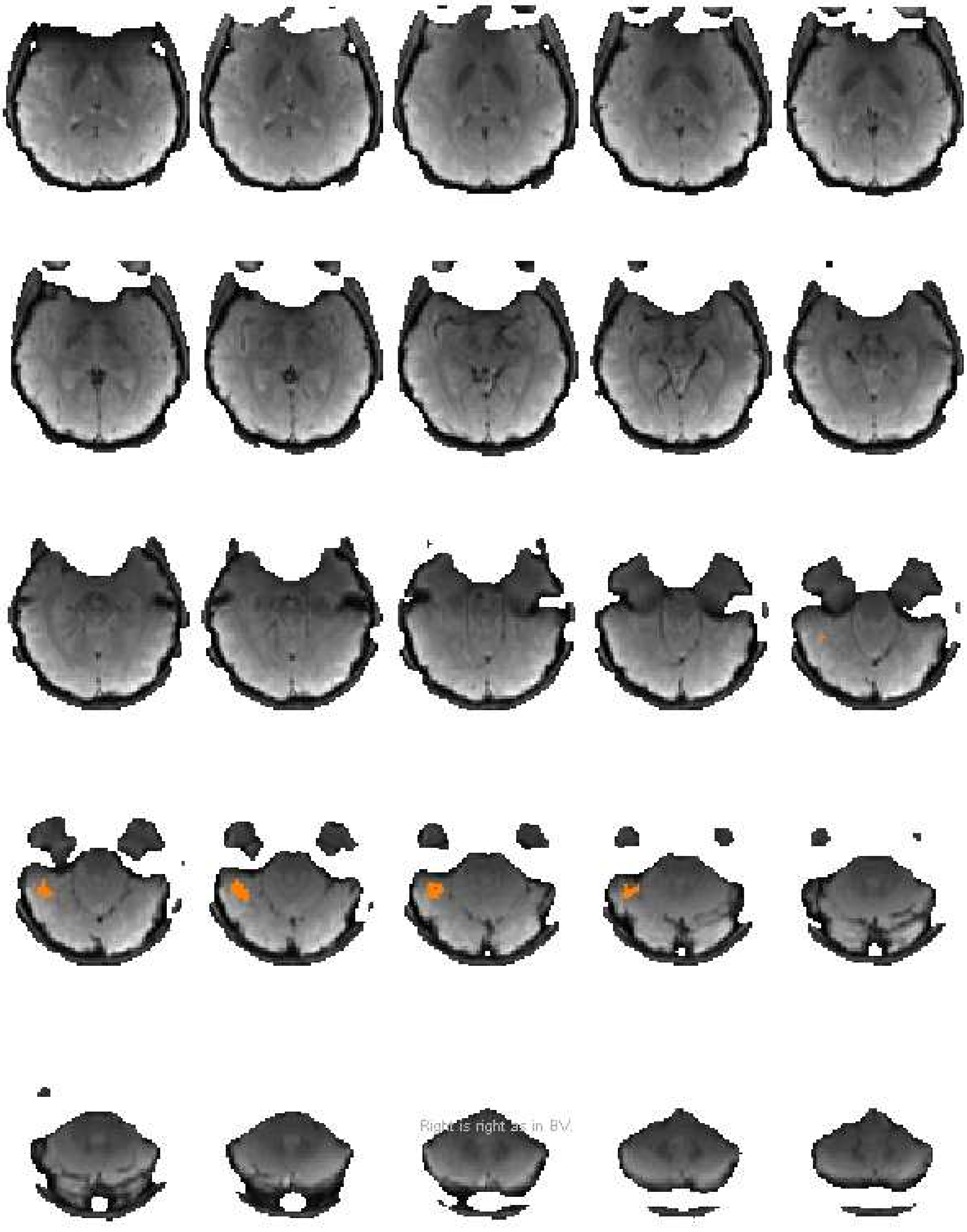

ROI3,2,4,4

subject 3
session 2
FFA_L
128 voxels

ROI for FFA_L (128)

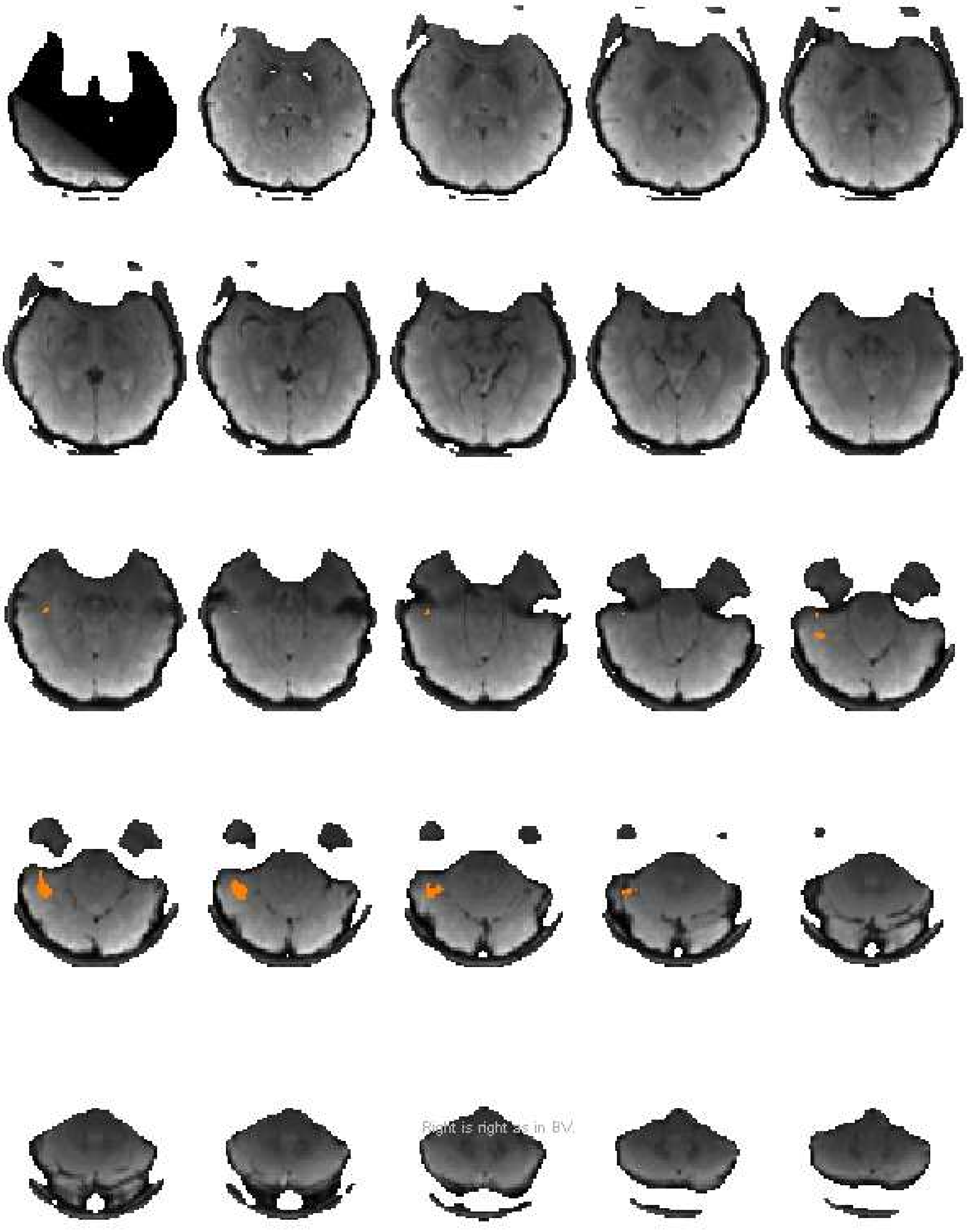

ROI4,1,4,4

subject 4
session 1
FFA_L
128 voxels

ROI for FFA_L (128)

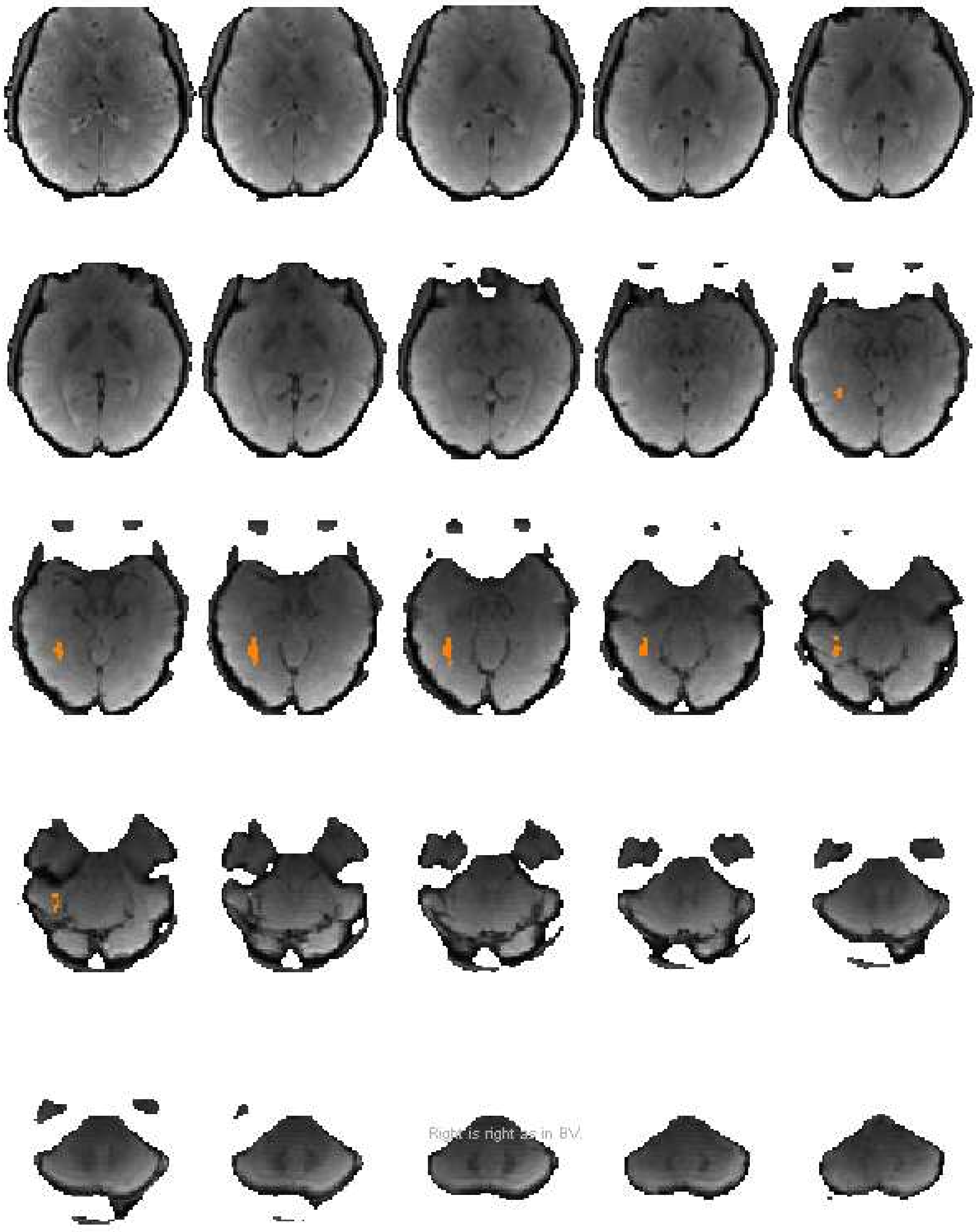

subject 4
session 2
FFA_L
128 voxels

ROI for FFA_L (128)

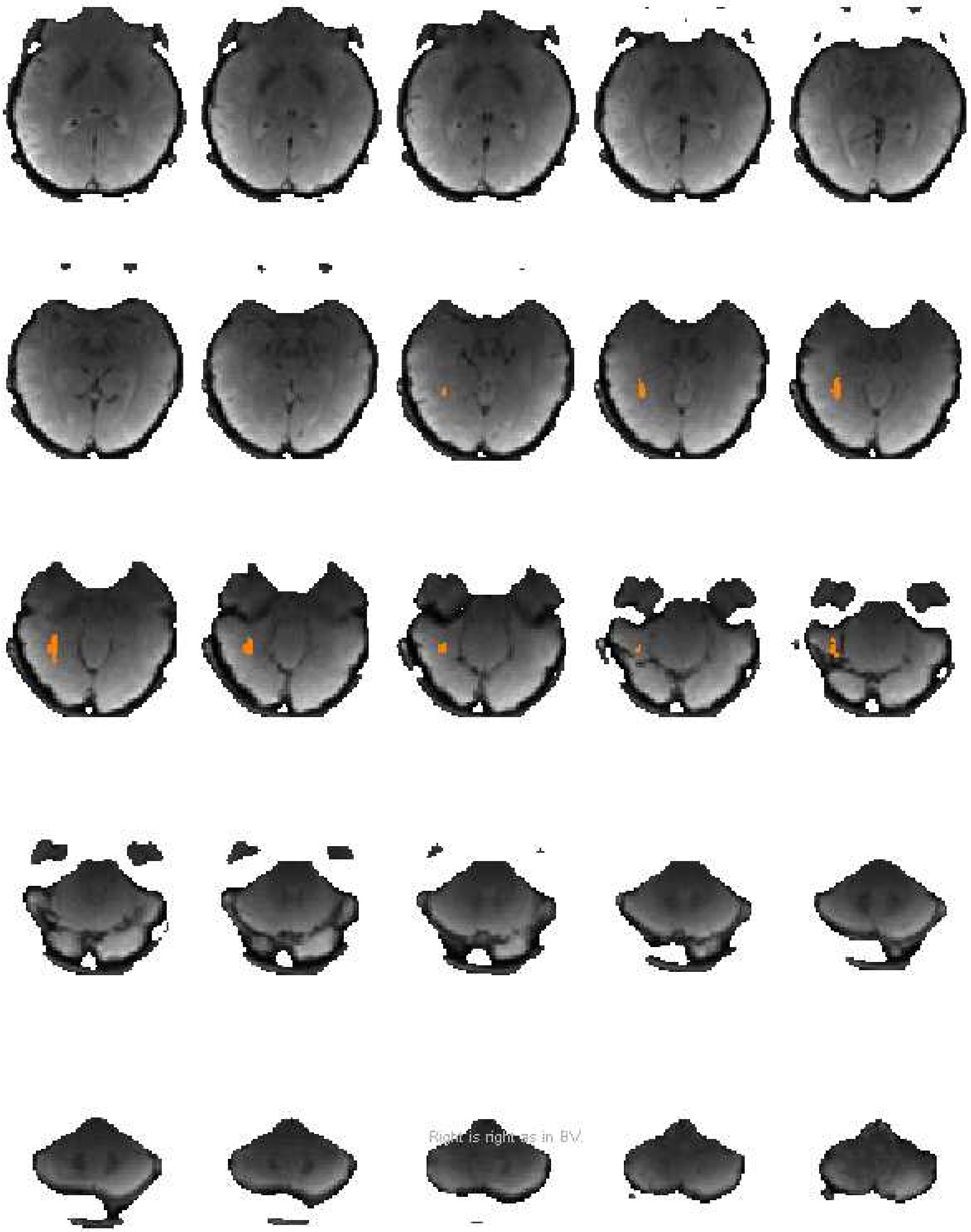

ROI1,1,5,4

subject 1
session 1
FFA_R
128 voxels

ROI for FFA_R (128)

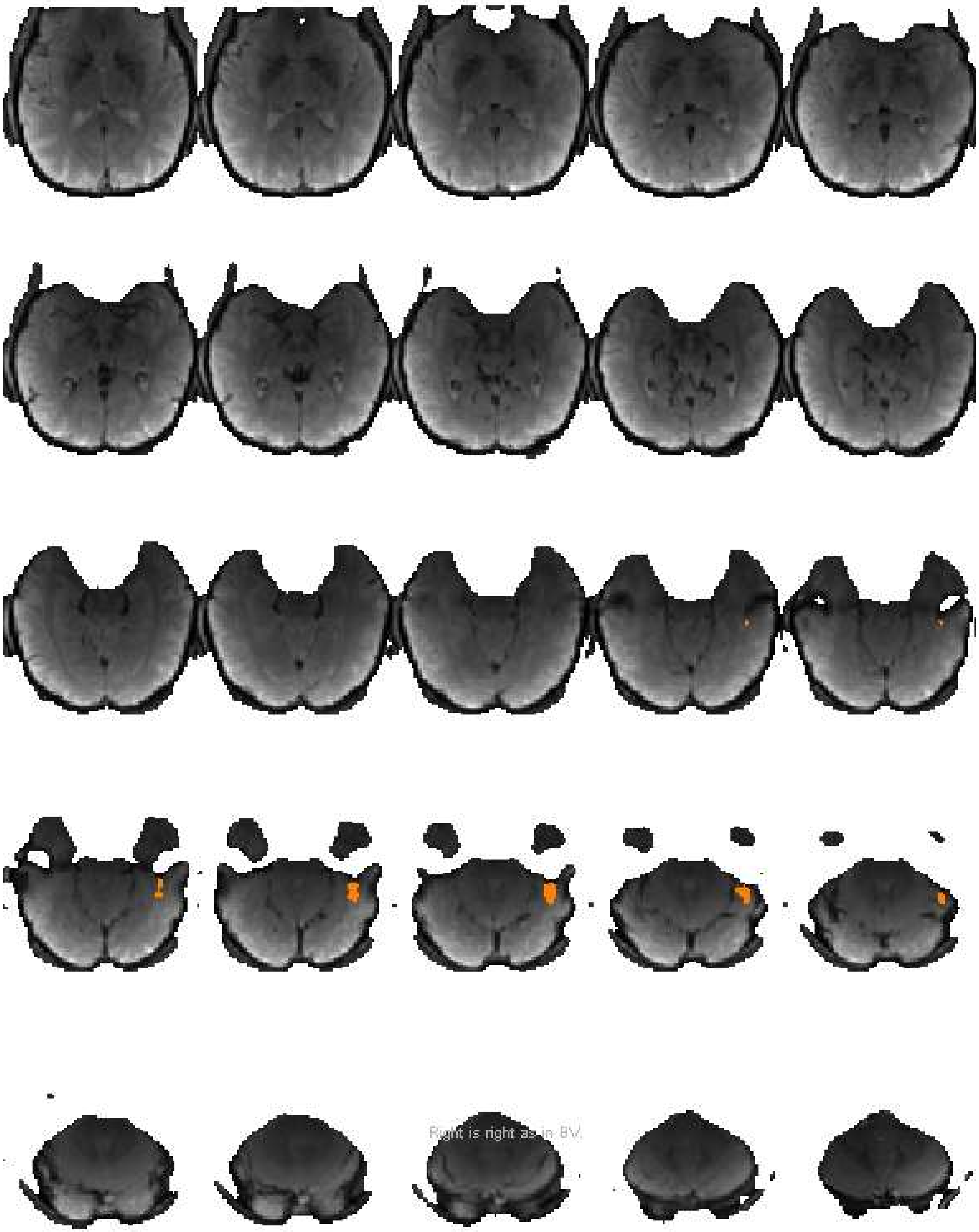

ROI1,2,5,4

subject 1
session 2
FFA_R
128 voxels

ROI for FFA_R (128)

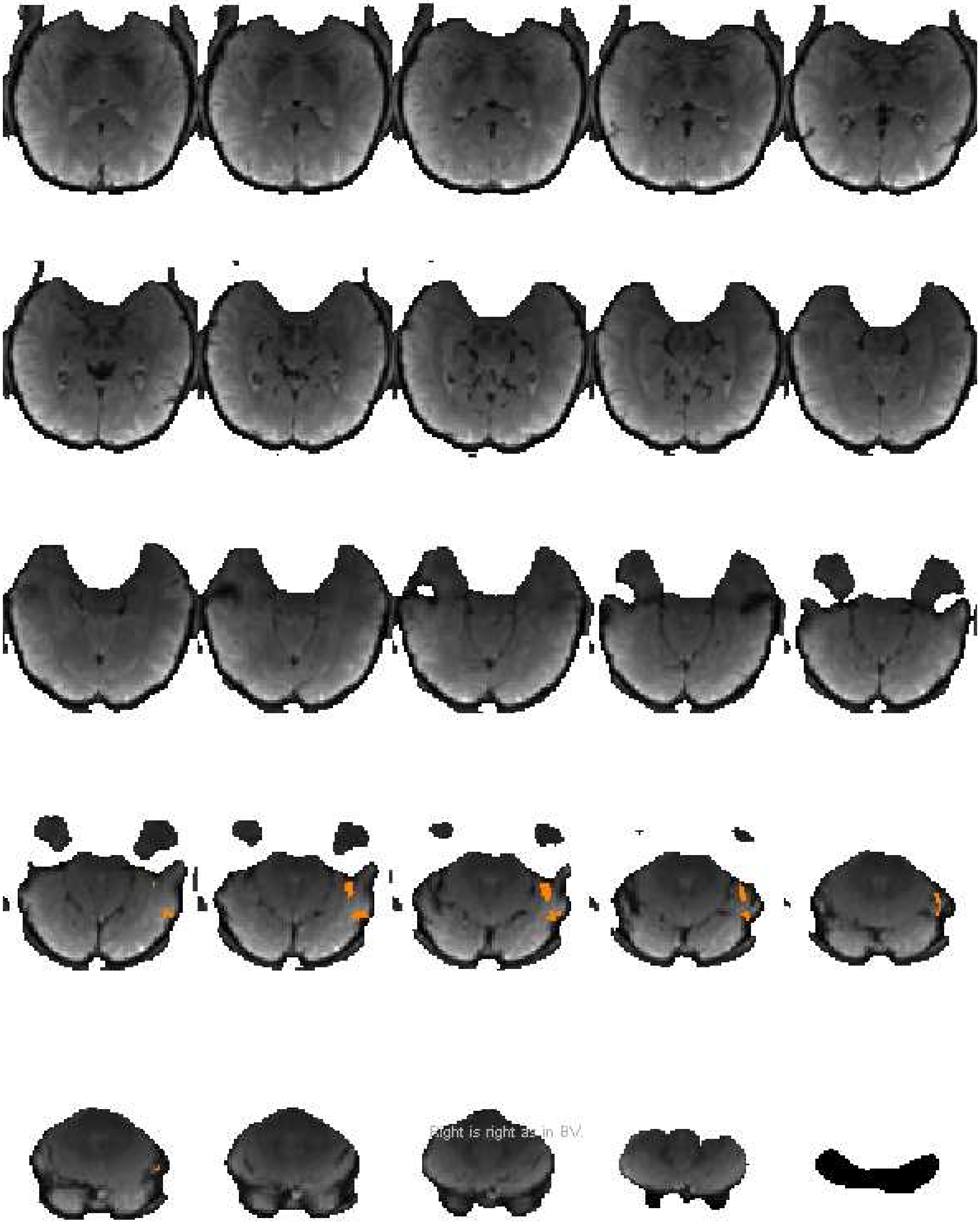

ROI2,1,5,4

subject 2
session 1
FFA_R
128 voxels

ROI for FFA_R (128)

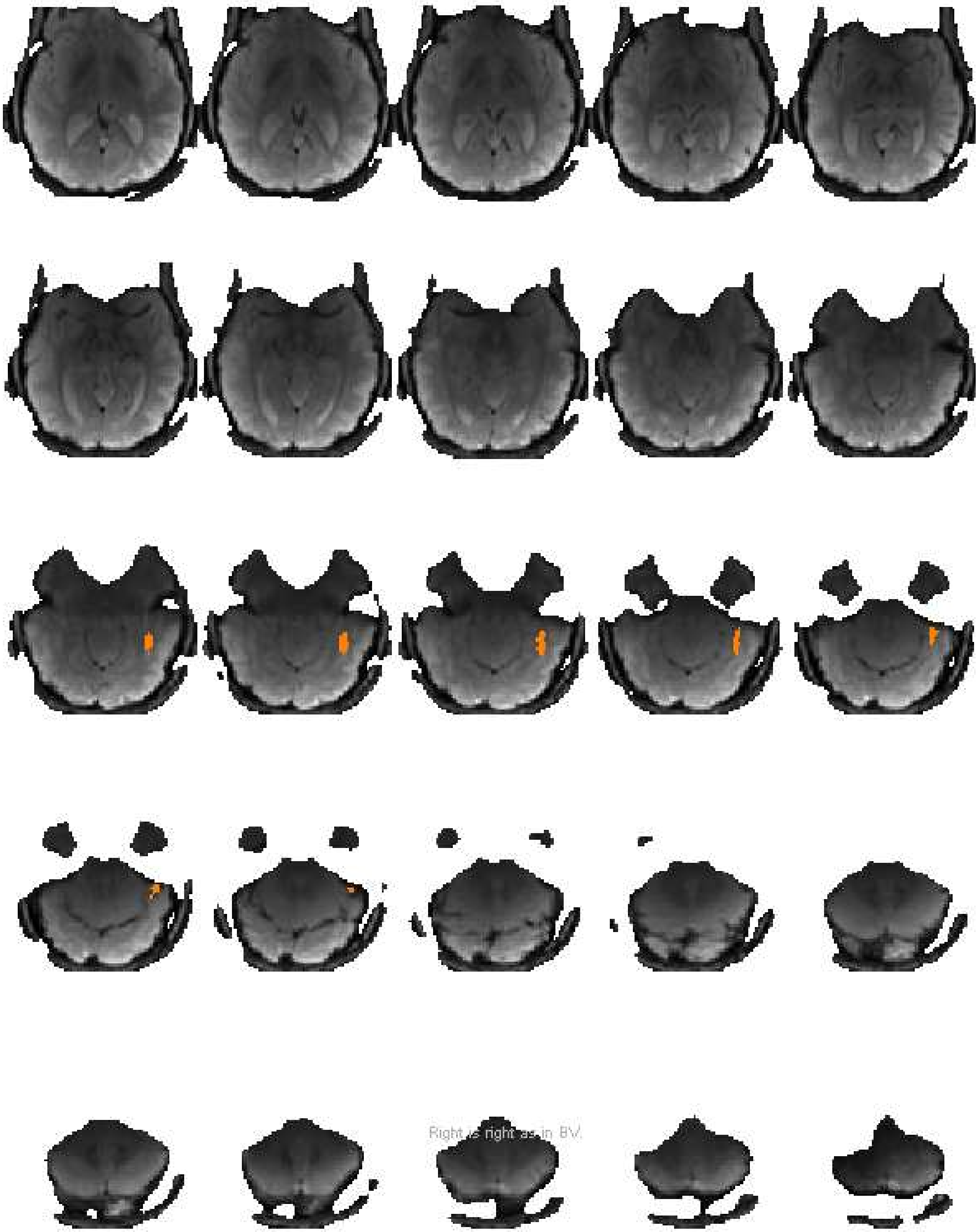

ROI2,3,5,4

subject 2
session 3
FFA_R
128 voxels

ROI for FFA_R (128)

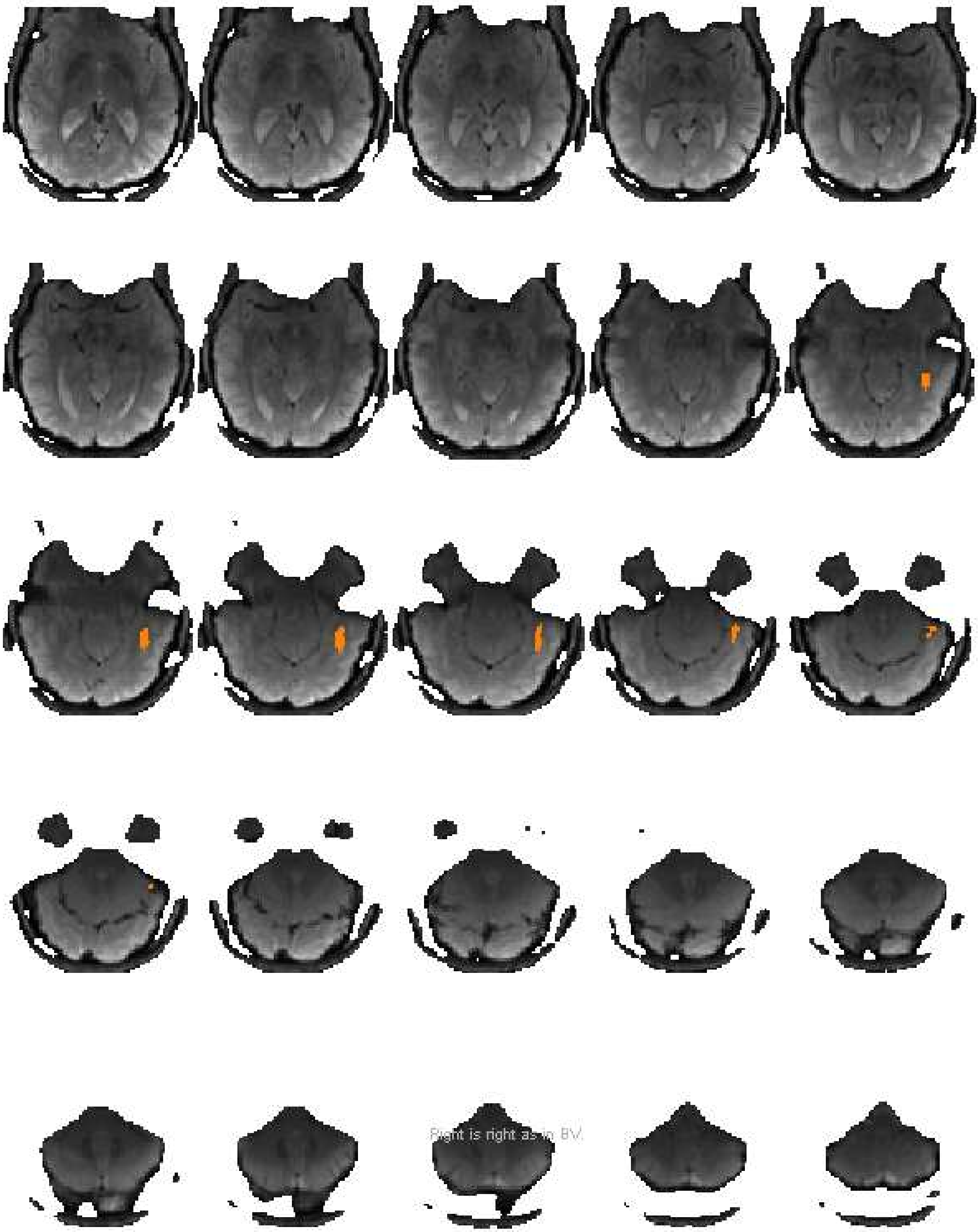

ROI3,1,5,4

subject 3
session 1
FFA_R
128 voxels

ROI for FFA_R (128)

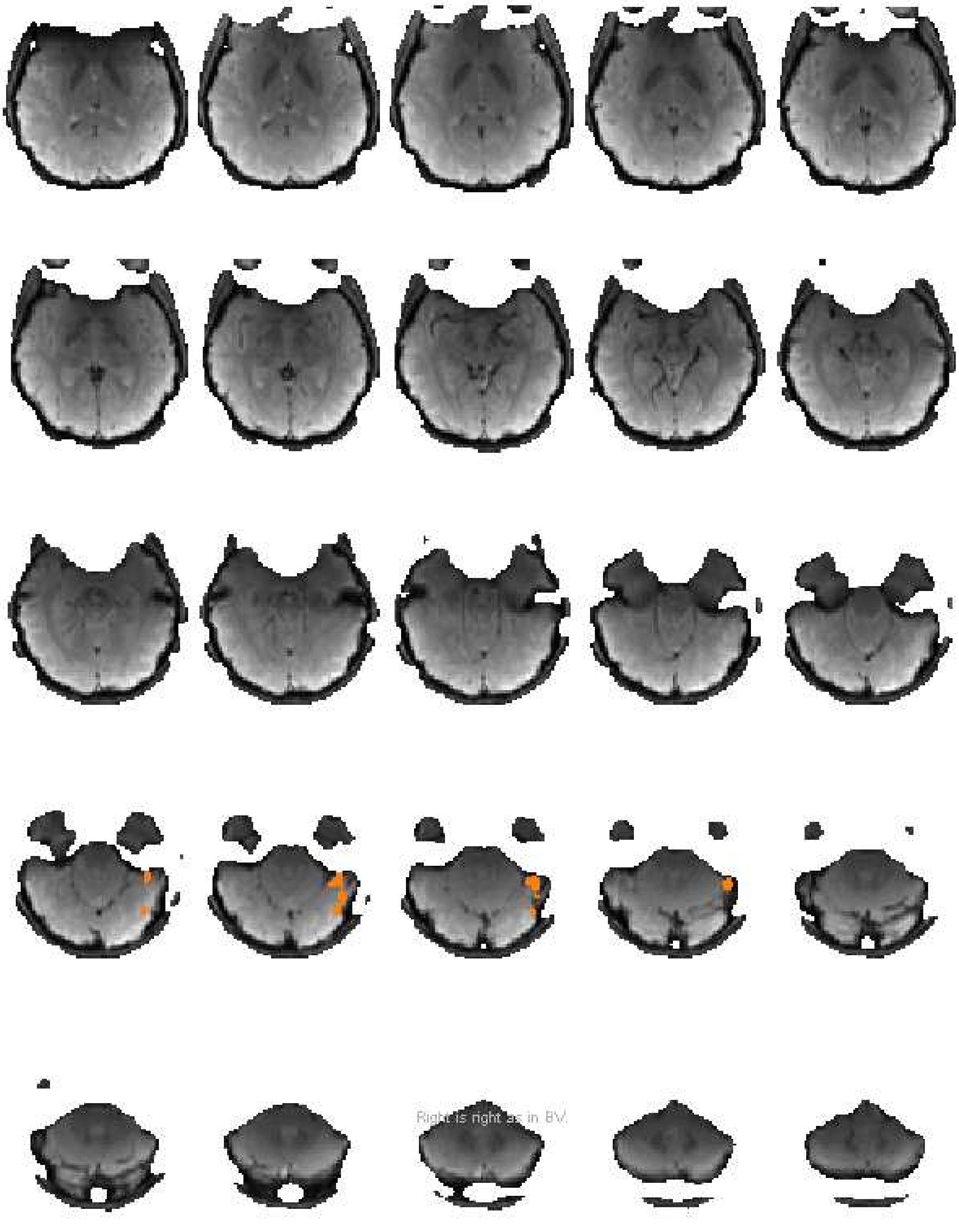

ROI3,2,5,4

subject 3
session 2
FFA_R
128 voxels

ROI for FFA_R (128)

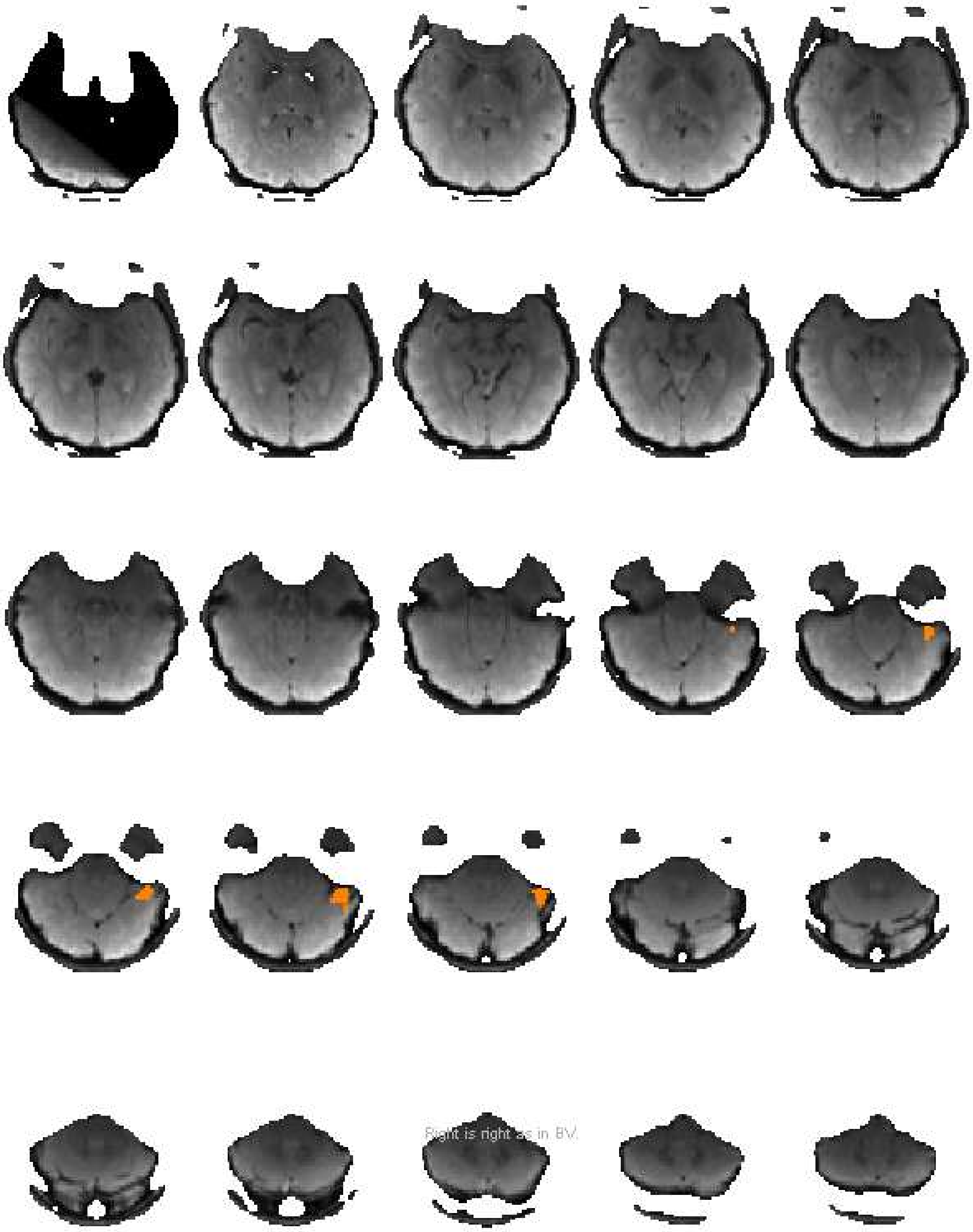

ROI4,1,5,4

subject 4
session 1
FFA_R
128 voxels

ROI for FFA_R (128)

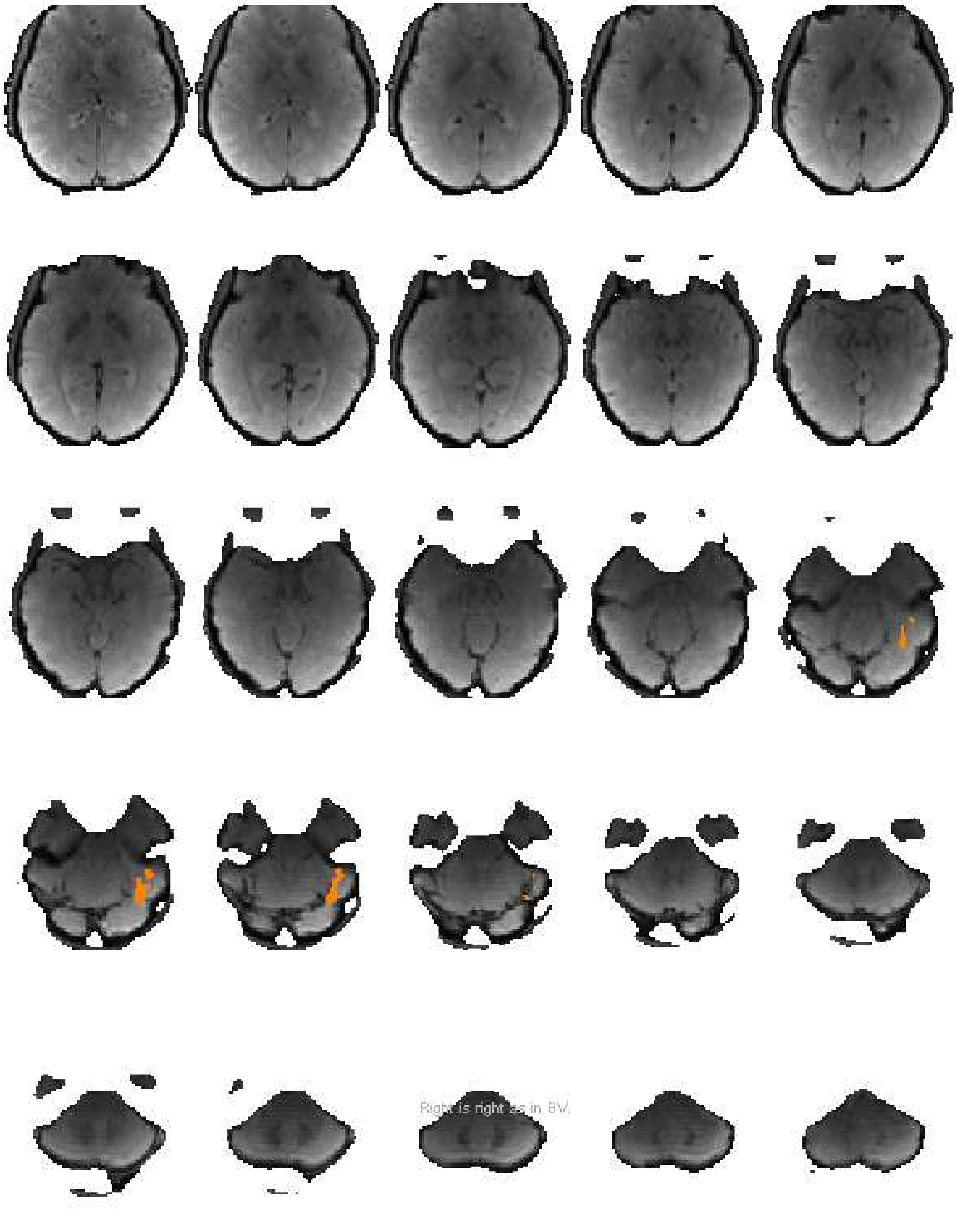

ROI4,2,5,4

subject 4
session 2
FFA_R
128 voxels

ROI for FFA_R (128)

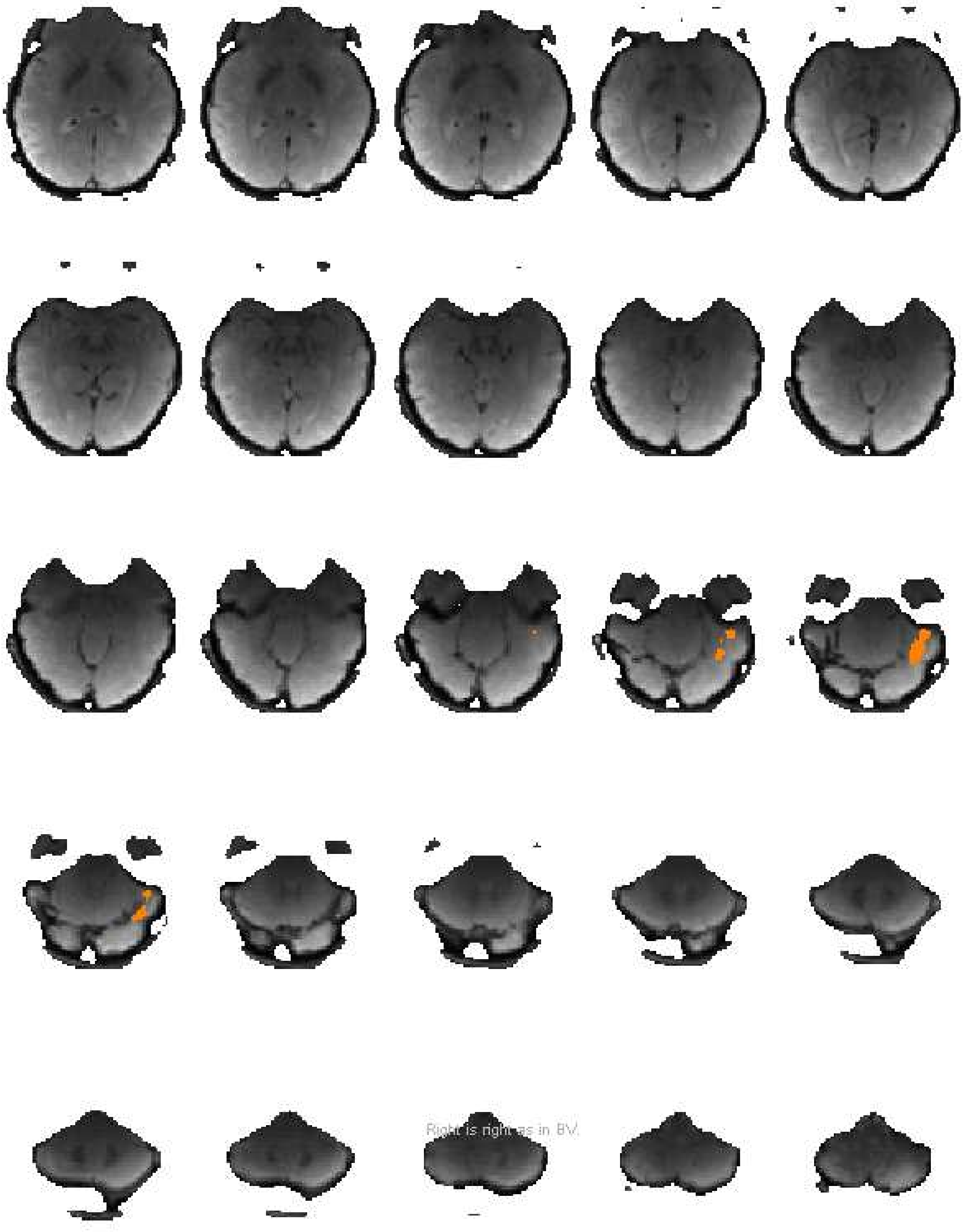

ROI1,1,6,4

subject 1
session 1
PPA_L
128 voxels

ROI for PPA_L (128)

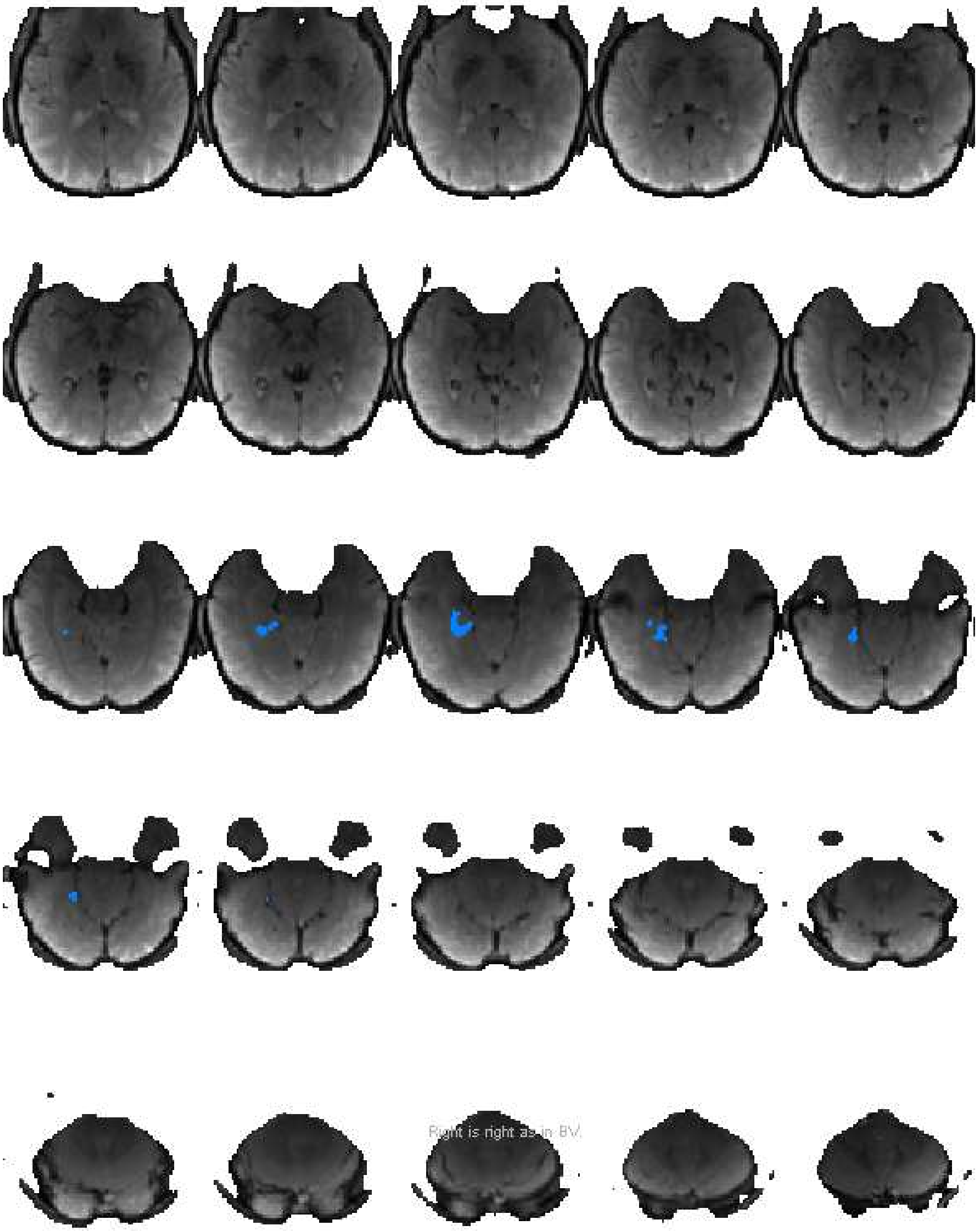

ROI1,2,6,4

subject 1
session 2
PPA_L
128 voxels

ROI for PPA_L (128)

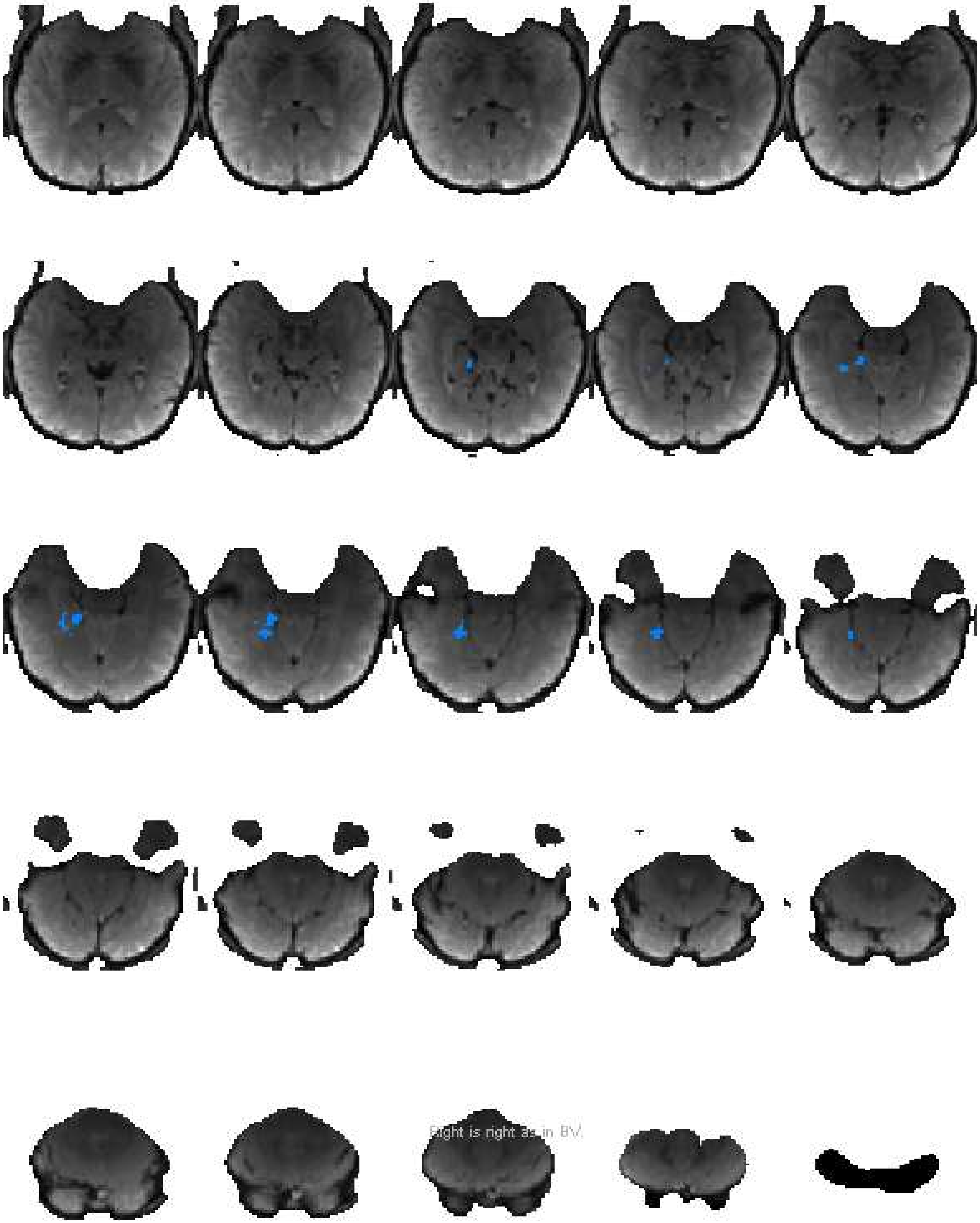

ROI2,1,6,4

subject 2
session 1
PPA_L
128 voxels

ROI for PPA_L (128)

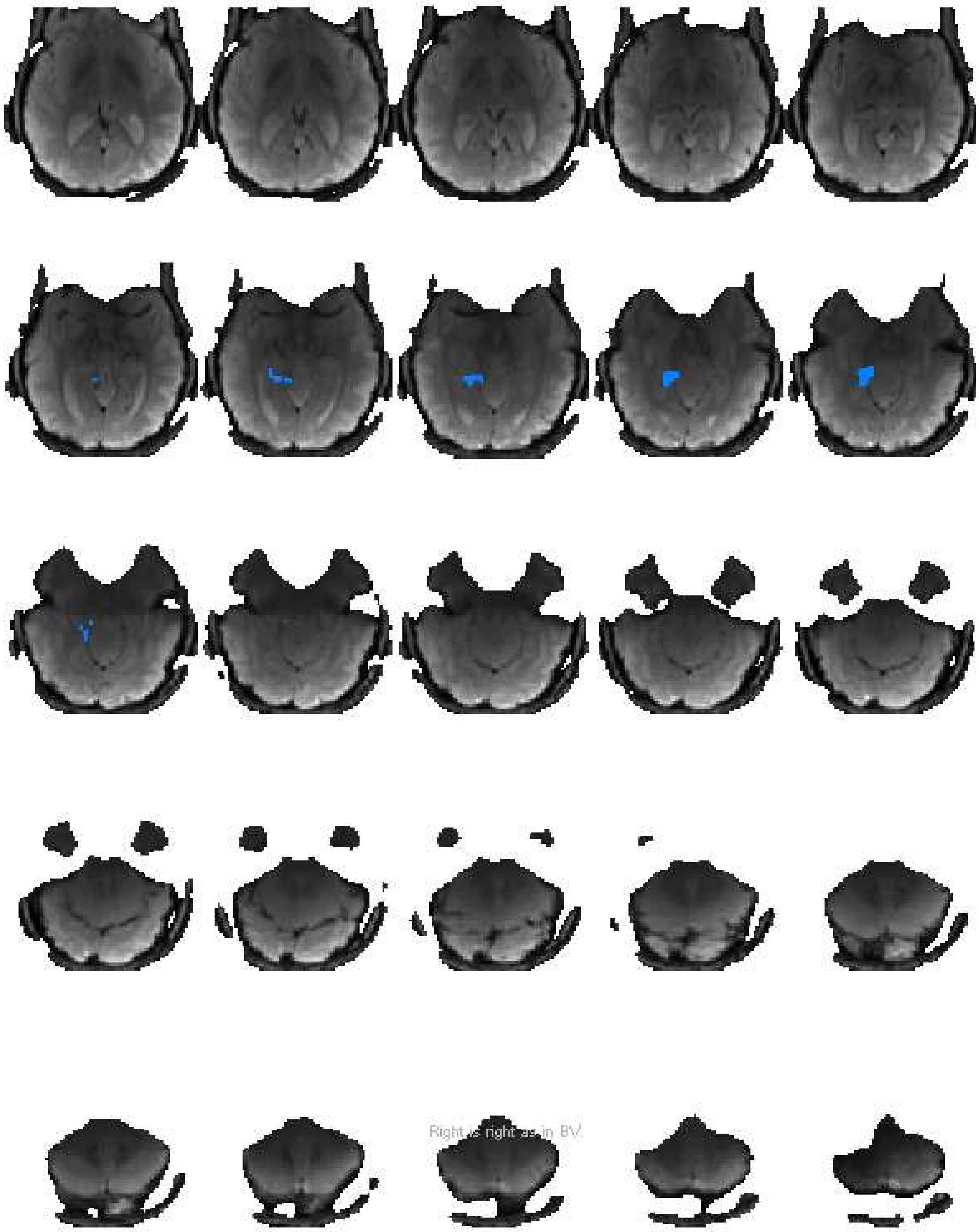

ROI2,3,6,4

subject 2

session 3

PPA_L

128 voxels

ROI for PPA_L (128)

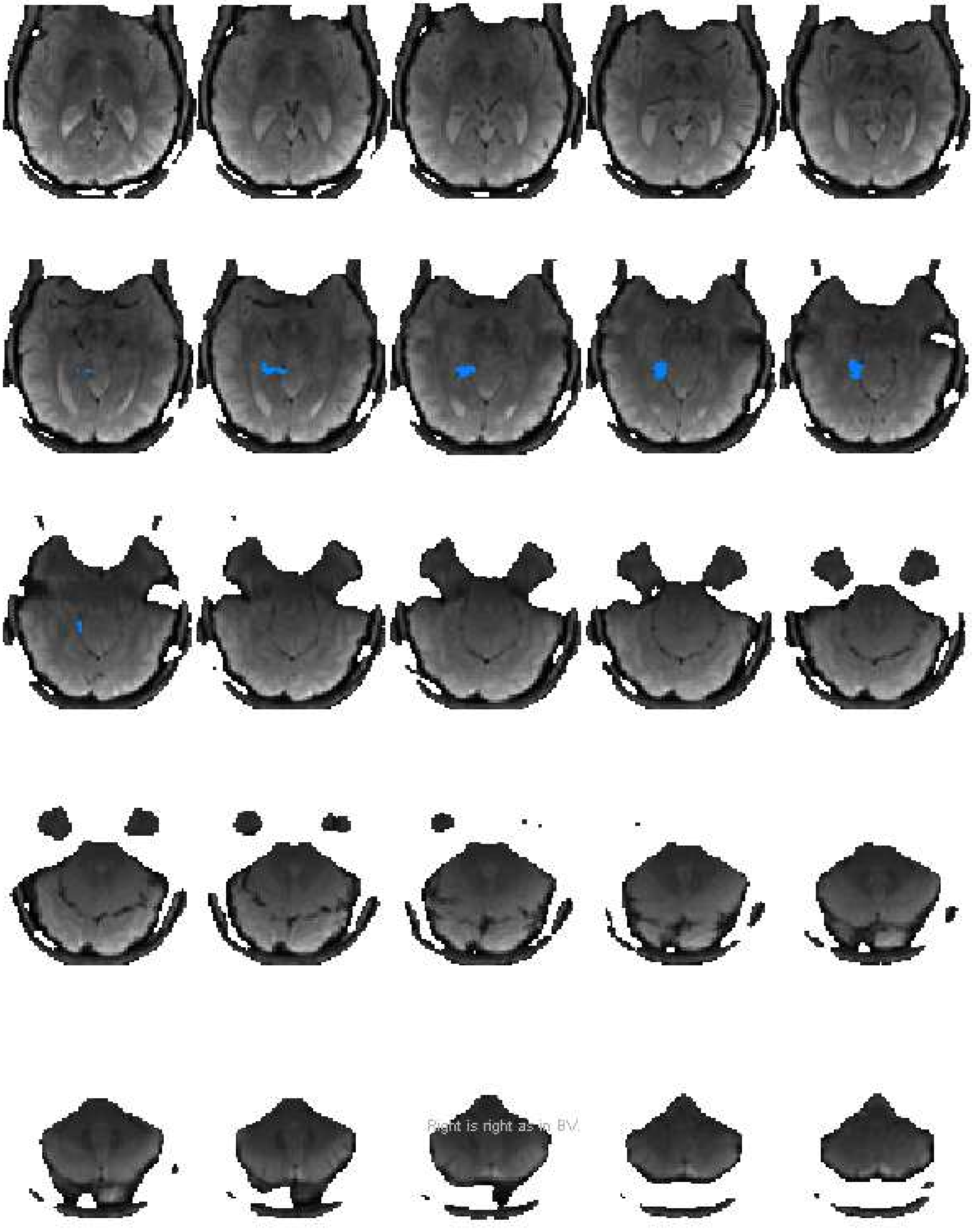

ROI3,1,6,4

subject 3
session 1
PPA$_L$
128 voxels

ROI for PPA$_L$ (128)

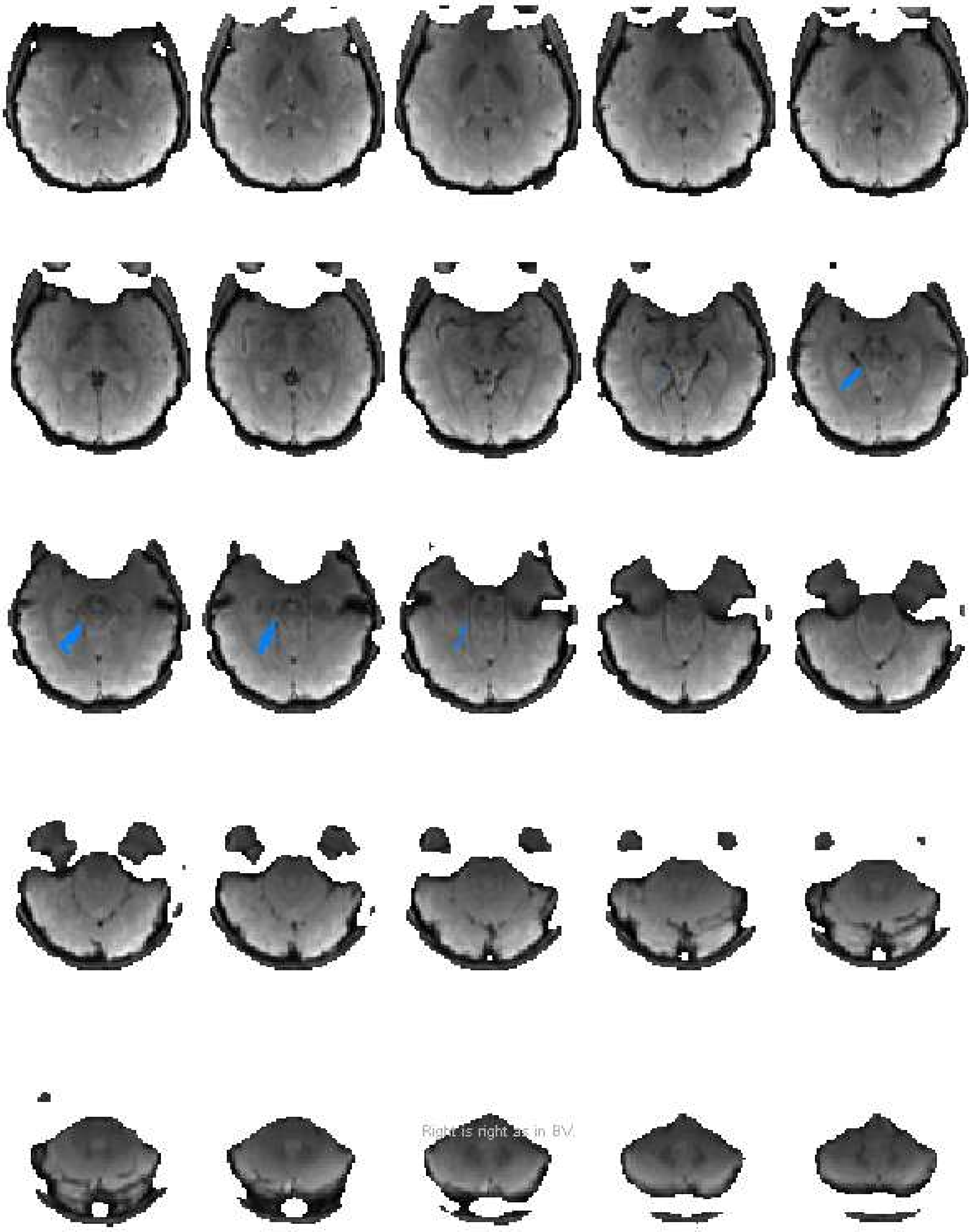

ROI3,2,6,4

subject 3
session 2
PPA_L
128 voxels

ROI for PPA_L (128)

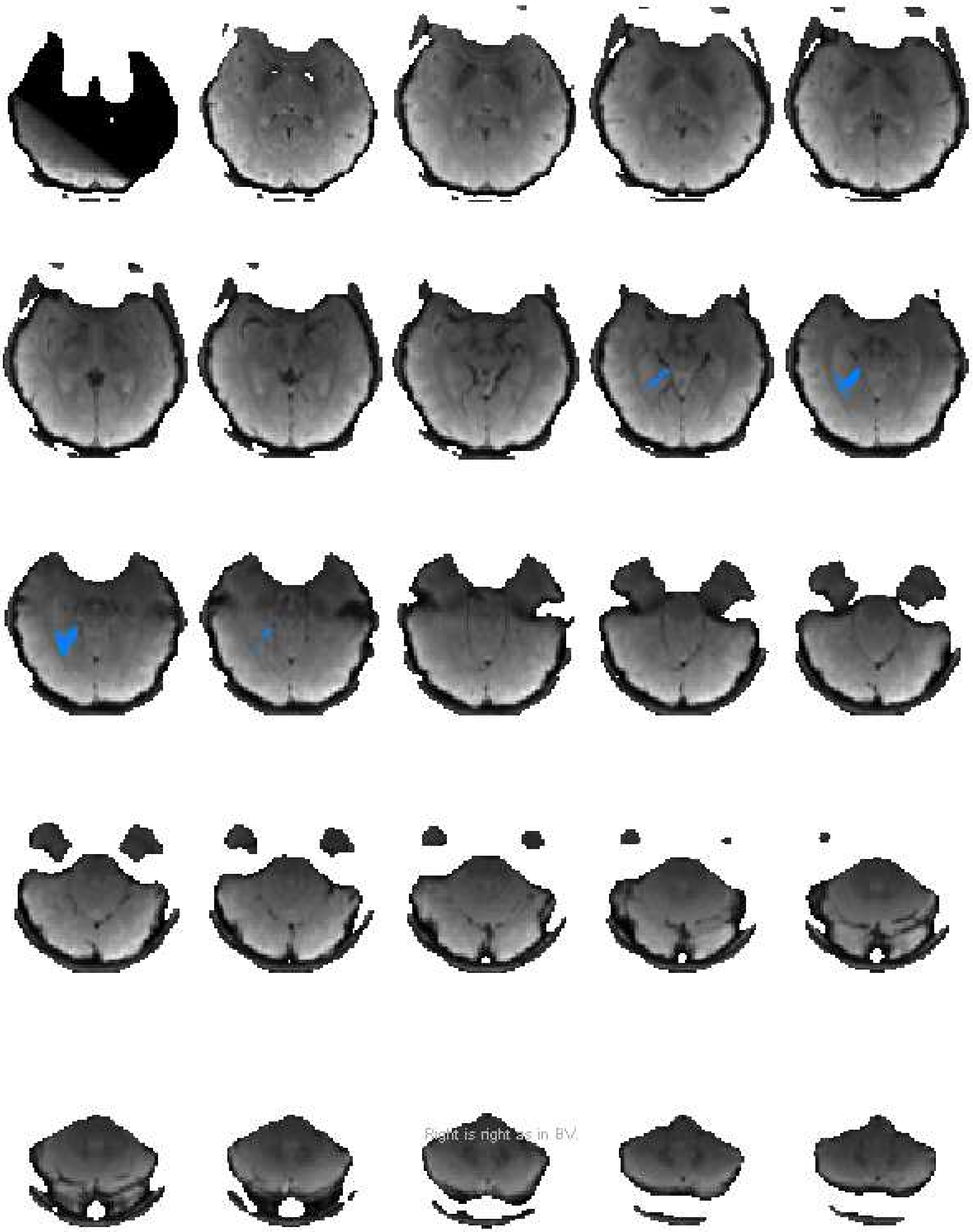

ROI4,1,6,4

subject 4
session 1
PPA_L
128 voxels

ROI for PPA_L (128)

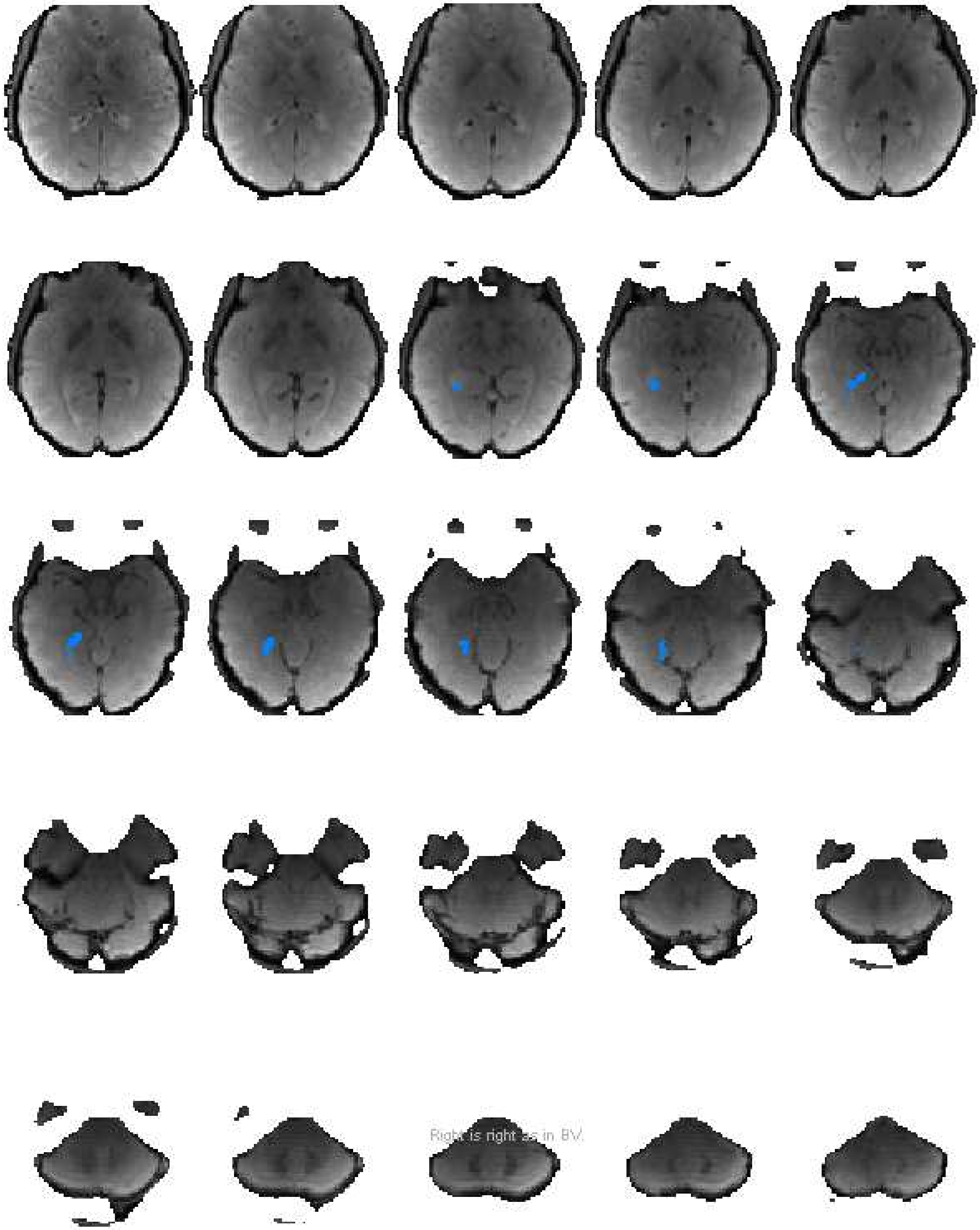

ROI4,2,6,4

subject 4

session 2

PPA_L

128 voxels

ROI for PPA_L (128)

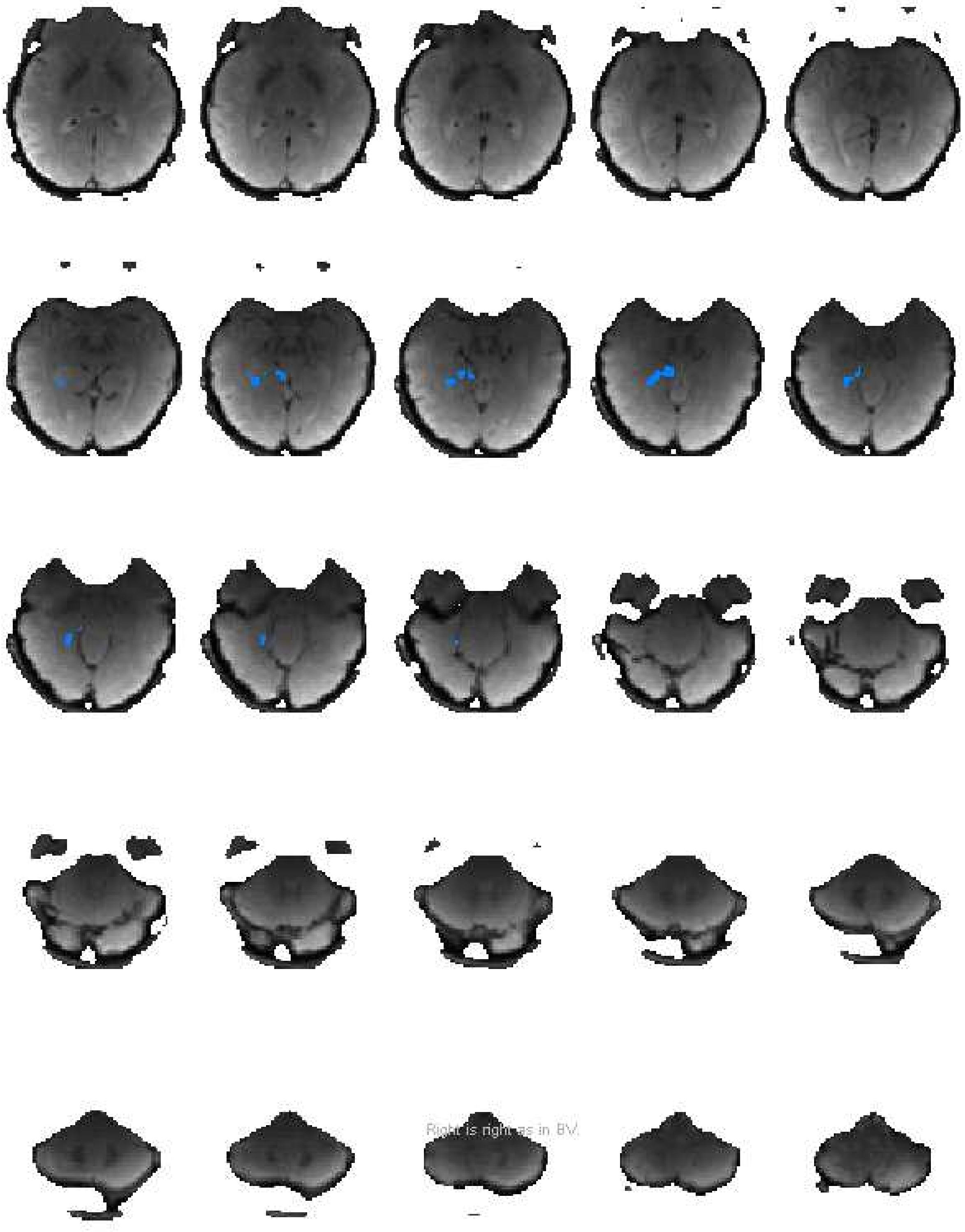

ROI1,1,7,4

subject 1
session 1
PPA_R
128 voxels

ROI for PPA_R (128)

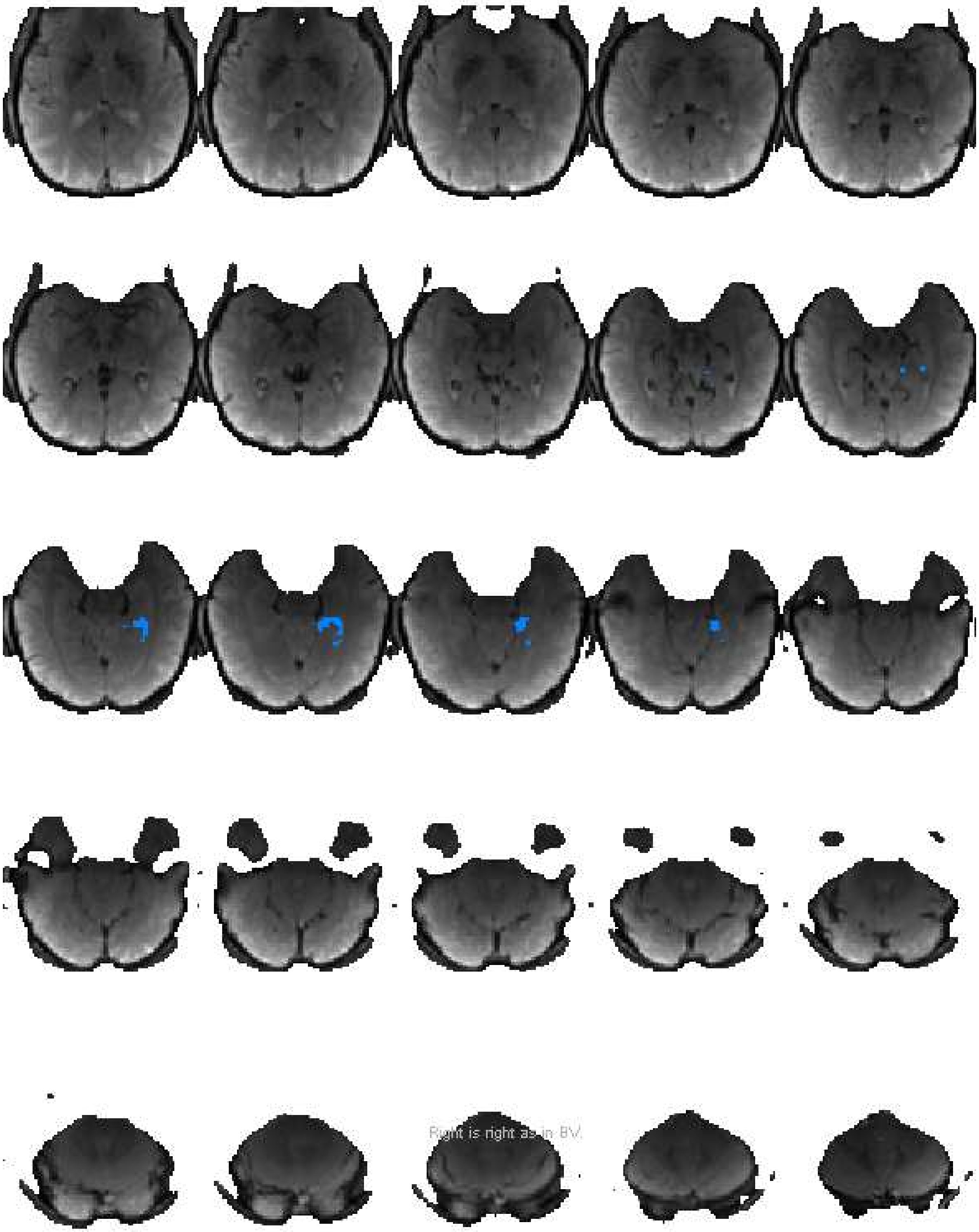

ROI1,2,7,4

subject 1
session 2
PPA_R
128 voxels

ROI for PPA_R (128)

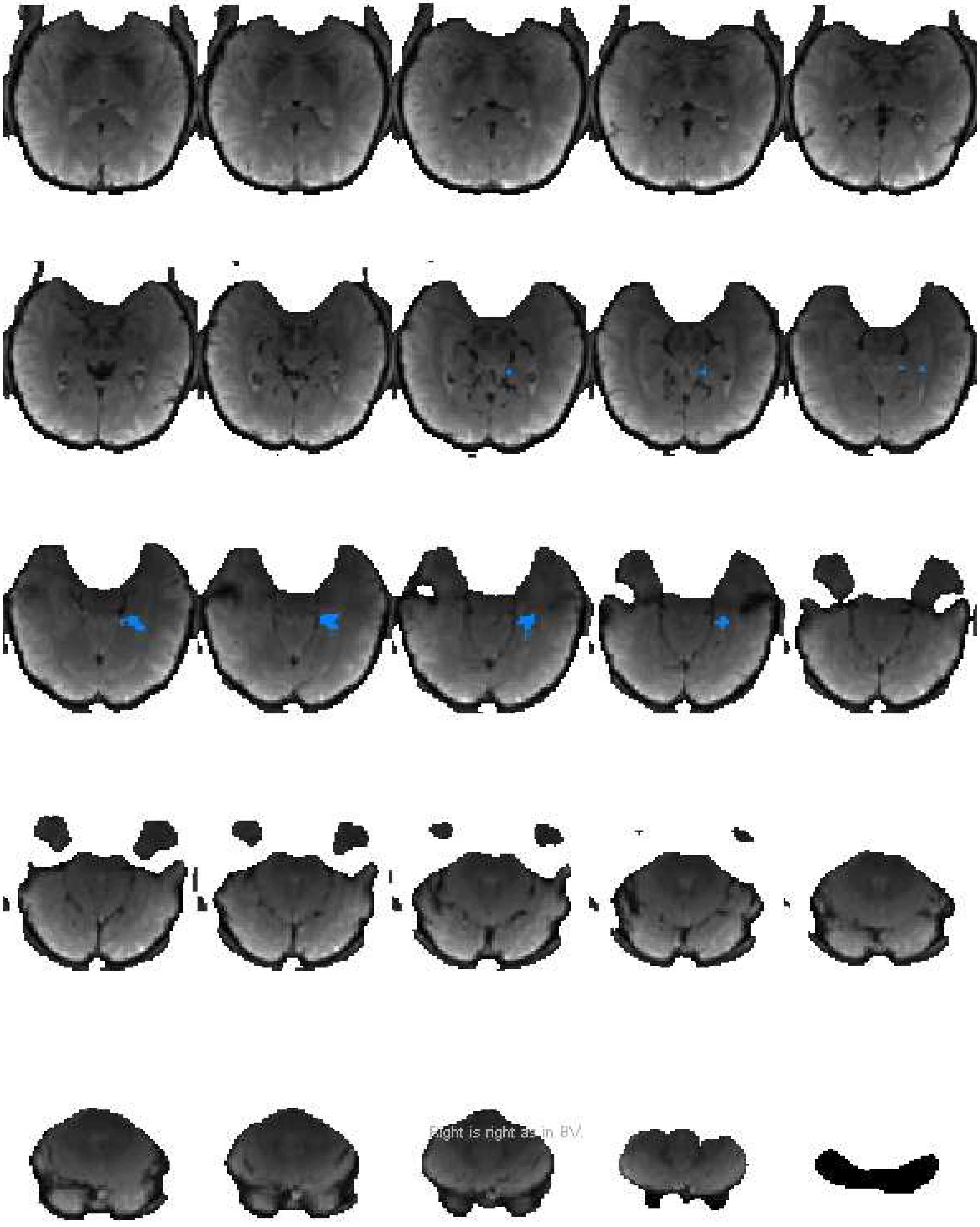

ROI2,1,7,4

subject 2
session 1
PPA_R
128 voxels

ROI for PPA_R (128)

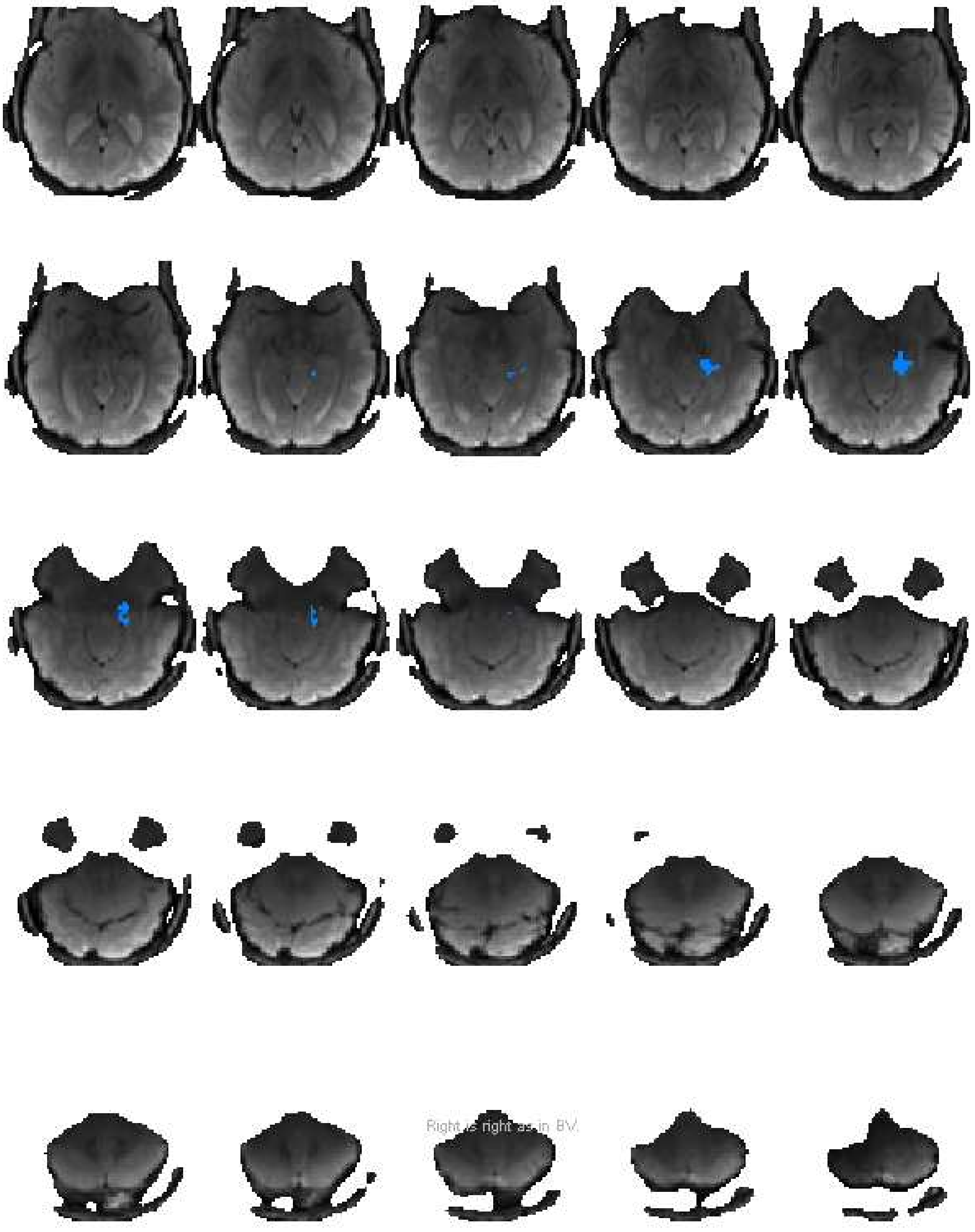

ROI2,3,7,4

subject 2
session 3
PPA_R
128 voxels

ROI for PPA_R (128)

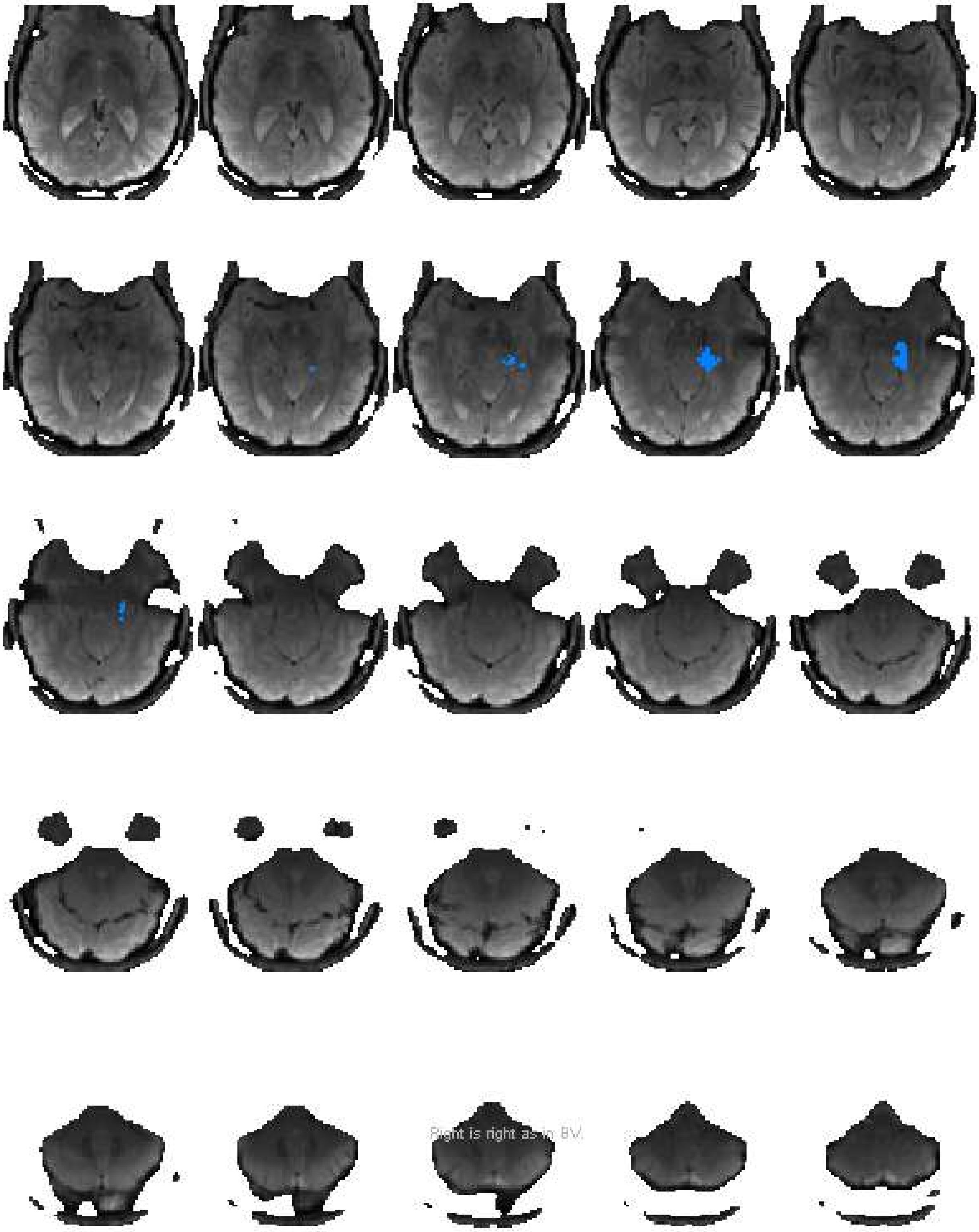

ROI3,1,7,4

subject 3
session 1
PPA_R
128 voxels

ROI for PPA_R (128)

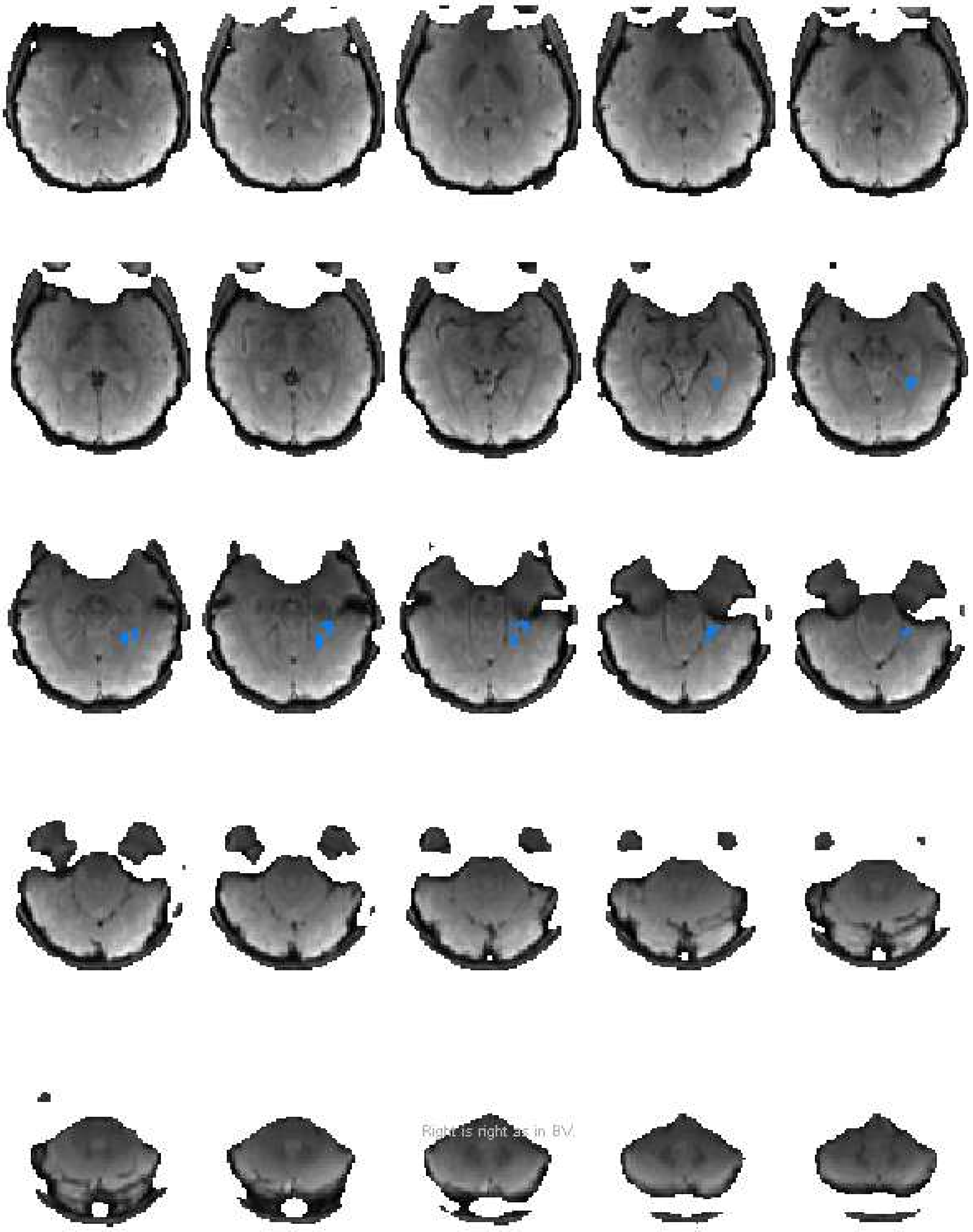

ROI3,2,7,4

subject 3
session 2
PPA_R
128 voxels

ROI for PPA_R (128)

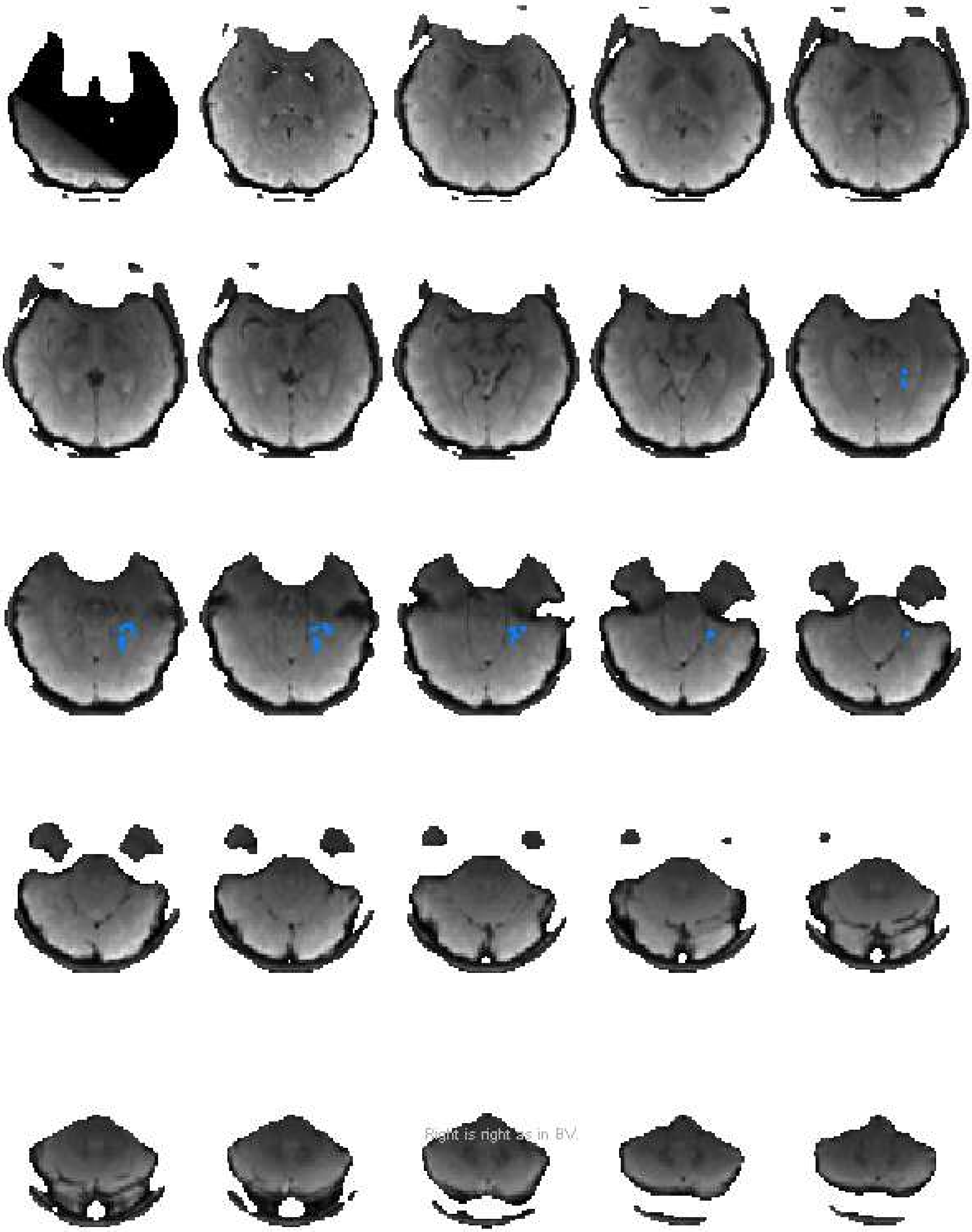

ROI4,1,7,4

subject 4
session 1
PPA_R
128 voxels

ROI for PPA_R (128)

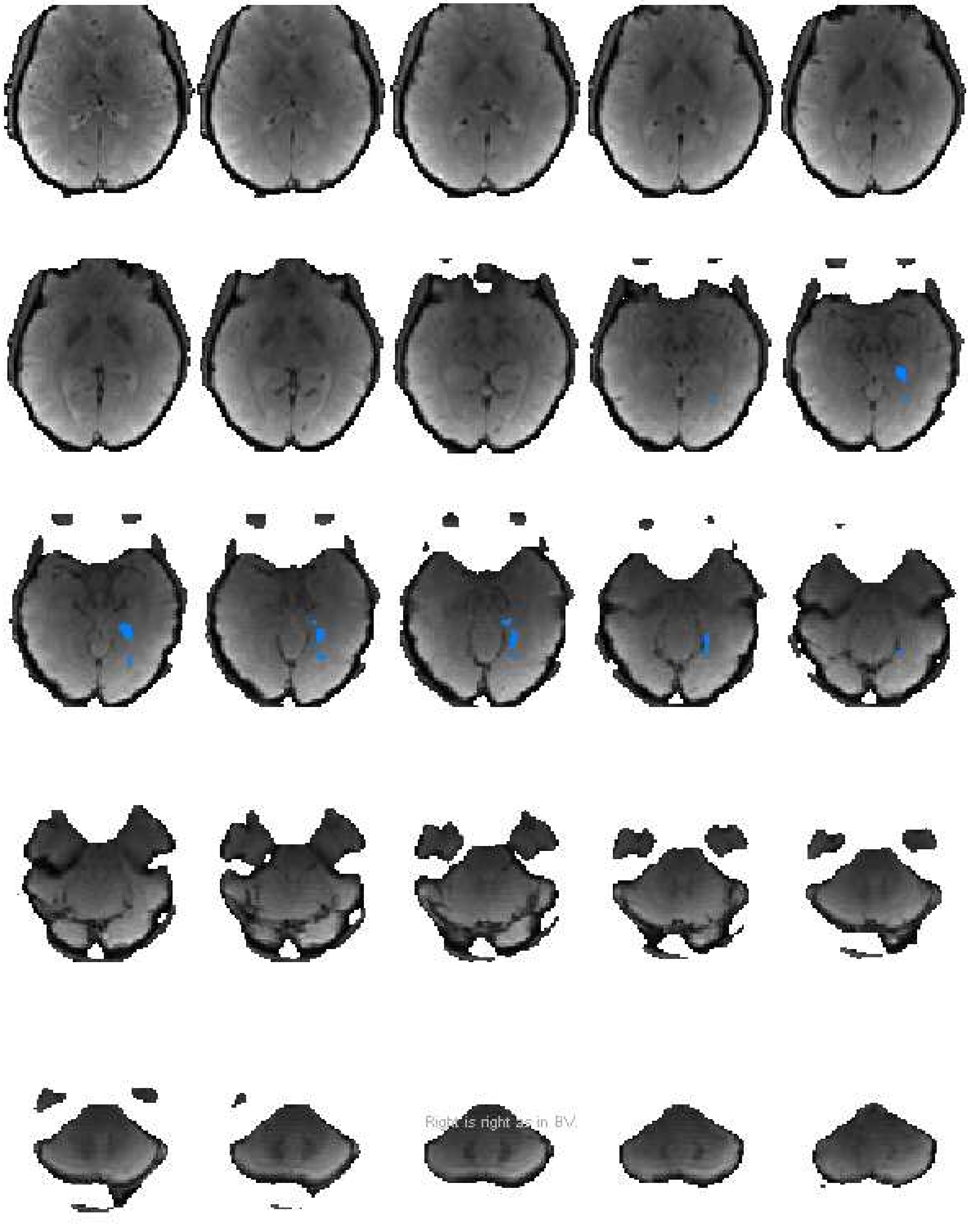

ROI4,2,7,4

subject 4
session 2
PPA_R
128 voxels

ROI for PPA_R (128)

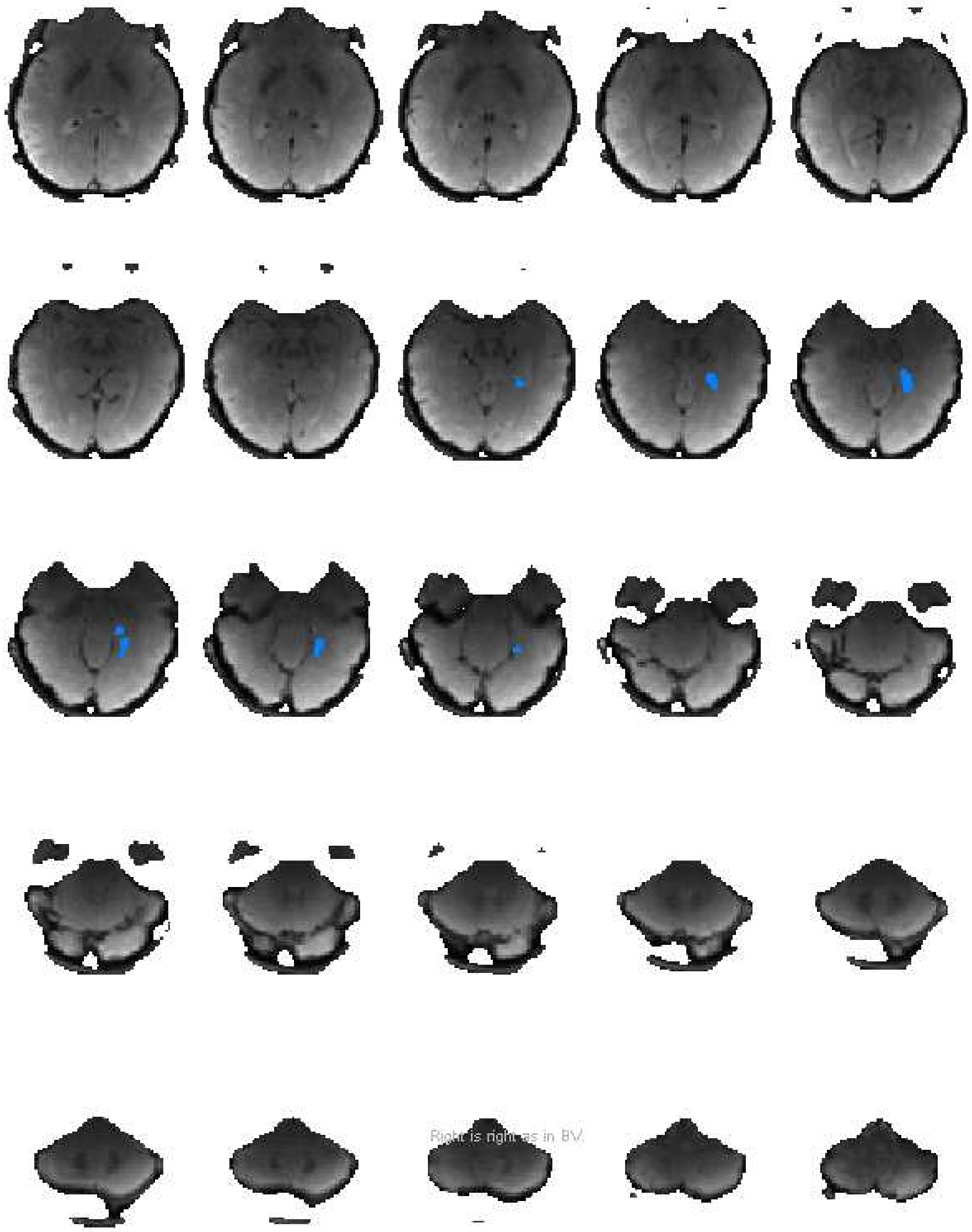

Supplement: S8 Fig — (PDF) [file pone.0223660.s008.pdf]
